# Ethiopian indigenous goats offer insights into past and recent demographic dynamics and local adaptation in sub‐Saharan African goats

**DOI:** 10.1111/eva.13118

**Published:** 2021-06-15

**Authors:** Getinet M. Tarekegn, Negar Khayatzadeh, Bin Liu, Sarah Osama, Aynalem Haile, Barbara Rischkowsky, Wenguang Zhang, Kassahun Tesfaye, Tadelle Dessie, Okeyo A. Mwai, Appolinaire Djikeng, Joram M. Mwacharo

**Affiliations:** ^1^ Department of Animal Production and Technology School of Animal Sciences and Veterinary Medicine Bahir Dar University Bahir Dar Ethiopia; ^2^ Department of Animal Breeding and Genetics Swedish University of Agricultural Sciences (SLU) Uppsala Sweden; ^3^ Department of Sustainable Agricultural Systems Division of Livestock Sciences University of Natural Resources and Life Sciences Vienna Austria; ^4^ Inner Mongolia Agricultural University Hohhot China; ^5^ The University of Queensland Saint Lucia QLD Australia; ^6^ Small Ruminant Genomics International Centre for Agricultural Research in the Dry Areas (ICARDA) Addis Ababa Ethiopia; ^7^ Department of Microbial, Cellular and Molecular Biology Addis Ababa University Addis Ababa Ethiopia; ^8^ International Livestock Research Institute (ILRI) Addis Ababa Ethiopia; ^9^ International Livestock Research Institute (ILRI) Nairobi Kenya; ^10^ Animal and Veterinary Sciences Group, SRUC and Centre for Tropical Livestock Genetics and Health (CTLGH) The Roslin Institute Easter Bush Midlothian UK

**Keywords:** autozygosity, diversity, effective population size, genome dynamics, LD decay, runs of homozygosity, selection signatures

## Abstract

Knowledge on how adaptive evolution and human socio‐cultural and economic interests shaped livestock genomes particularly in sub‐Saharan Africa remains limited. Ethiopia is in a geographic region that has been critical in the history of African agriculture with ancient and diverse human ethnicity and bio‐climatic conditions. Using 52K genome‐wide data analysed in 646 individuals from 13 Ethiopian indigenous goat populations, we observed high levels of genetic variation. Although runs of homozygosity (ROH) were ubiquitous genome‐wide, there were clear differences in patterns of ROH length and abundance and in effective population sizes illustrating differences in genome homozygosity, evolutionary history, and management. Phylogenetic analysis incorporating patterns of genetic differentiation and gene flow with ancestry modelling highlighted past and recent intermixing and possible two deep ancient genetic ancestries that could have been brought by humans with the first introduction of goats in Africa. We observed four strong selection signatures that were specific to Arsi‐Bale and Nubian goats. These signatures overlapped genomic regions with genes associated with morphological, adaptation, reproduction and production traits due possibly to selection under environmental constraints and/or human preferences. The regions also overlapped uncharacterized genes, calling for a comprehensive annotation of the goat genome. Our results provide insights into mechanisms leading to genome variation and differentiation in sub‐Saharan Africa indigenous goats.

## 1 INTRODUCTION

Threats to biodiversity, and projected future food demands and climatic conditions, underscore the urgent need to characterize, monitor and maintain agricultural biodiversity. Data on molecular changes have been critical in understanding genome architecture, maintaining biodiversity and fitness and exploring impacts of genome evolution. This has been made possible through the investigation of genetic diversity and structure, demographic dynamics (Bosse et al., 2012) and assessment of autozygosity under varying degrees of reproductive isolation and inbreeding (Ceballos, Joshi, Clark, Ramsay, & Wilson, 2018; Kirin et al., 2010; Peripolli et al., 2016). As livestock dispersed from their centres of domestication, they encountered diverse environments with unique climatic, anthropological and biophysical limits. These animals responded to the niche‐specific environmental pressures through behavioural and biological adjustments. The former provided short‐term buffer against many of the stressors, and the latter ensured long‐term survival through physiological and/or genetic adaptation.

Among domestic ungulates, goats have the longest socio‐cultural and economic co‐existence with humans. They were domesticated around 11,000 years ago in the Fertile Crescent of Southwest Asia and adjacent areas (Zeder & Hesse, 2000). Since their domestication, goats have been contributing to human cultural and socio‐economic transformations that have shaped ancient and modern human civilizations and the goat's genomes. Their rusticity and resilience have allowed them to cope and adapt to a wide range of environments. However, the evolutionary history of goats is complex with two contrasting hypotheses explaining their domestication and little geographic pattern in their maternal diversity. Luikart et al. (2001) observed at least six distinct mtDNA haplogroups with little geographic partitioning. They interpreted this to be the consequence of multiple domestication events and ease of translocation through commercial activities. Naderi et al. (2008) on the other hand suggested that such diversity of mtDNA haplogroups was compatible with a single domestication event, followed by a phase of human management of wild or semi‐domesticated goats comprising multiple mtDNA lineages, prior to their global dispersion. Evidence from the analysis of ancient goat genomes suggests the domestication of multiple divergent wild goats in a dispersed manner resulting in distinct Neolithic populations that contributed disproportionately to modern goat genomes (Daly et al., 2018). The initial diffusion of goats to Africa is also complex. Archaeological evidence suggests at least three entry points into the continent (Gifford‐Gonzalez & Hanotte, 2011). At least two mtDNA haplogroups have also been observed in Kenyan (Kibegwa, Githui, Jung'a, Badamana, & Nyamu, 2015), Ethiopian (Tarekegn et al., 2018), Sudanese (Sanhory, Giha, Ebrahim, Eldin, & Gornas, 2014) and Egyptian (Naderi et al., 2008) indigenous goats, suggesting the influence of at least two independent maternal lineages.

Palaeogenomic evidence has shown that the north‐eastern and Horn of Africa region were at the centre of a vast web of maritime and terrestrial routes of ancient and modern trade and thus a major entry point of domesticates into the continent (Fuller & Boivin, 2009; Marshall, 2000). The Horn of Africa landscape exhibit considerable changes in elevation within short geographic distances due to complex volcano‐tectonic activities over the past millennia (Mohr, 1971). In particular, the Ethiopian highlands, which range from 125 m below sea level in the Afar depression to altitudes exceeding 4,000 metres above sea level in the Arsi‐Bale mountains, form an extensive uplifted plateau, that is delimited by pronounced escarpments (Umer et al., 2007). The altitudinal gradients result in a wide range of agro‐eco‐climates and production environments. The region is also characterized by human ethnic diversity of ancient origin that has been associated with livestock husbandry. Palaeo‐climatic data have also revealed periods of prolonged and severe droughts in the region with profound impacts (Verschuren, Laird, & Cumming, 2000). These factors make the region particularly attractive for investigating how the genomes of indigenous livestock might have been shaped by past and recent events. Here, we generated 52K SNP genotypes in 13 populations of Ethiopian indigenous goats. The data were used to investigate past and recent demographic dynamics through the analysis of genome‐wide diversity and admixture, autozygosity and evidence for selection.

## 2 MATERIALS AND METHODS

### 2.1 Animals and genotypes

The animals used in this study were provided by farmers and pastoralists who participated in the study by agreeing to have their animals sampled. The sampling was done following standard guidelines and procedures of the Ministry of Livestock and Fisheries of the Federal Democratic Republic of Ethiopia. No ethical approvals were required at the time.

In total, 646 unrelated goats from 13 populations (FARM‐Africa 1996; Table 1) were sampled by collecting whole blood from two mature unrelated animals per flock via jugular venipuncture into EDTA vacutainers. Prior to sampling, and in the absence of written pedigree records, flock owners were interviewed in detail on the extent of genetic relationships between their animals. Genomic DNA was recovered with the salting out procedure (Shinde, Gujar, Patil, Satpute, & Kashid, 2008). Genotyping was performed with the Illumina® Caprine SNP 52K panel featuring 53,347 SNPs (single nucleotide polymorphisms). Using PLINK v1.9 (Purcell, Neale, Todd‐Brown, Thomas, & Ferreira, 2007), SNPs with call rates <95%, minor allele frequency (MAF) <0.01, unknown and redundant genome coordinates, nonautosomal location and those not in Hardy–Weinberg equilibrium (HWE; *p* < .001), and samples with >5% missing genotypes, were filtered from the data set. This left 44,723 SNPs and 628 goats for analysis. A subset of these samples had been analysed for mtDNA D‐loop variation (Tarekegn et al., 2018).

**Table 1:** Genetic diversity and variation statistics for 13 Ethiopian indigenous goat populations (mean ± *SD*)

Population	Abbreviation	Regions	*N*	H_O_	H_E_	P_N_	D_ST_	*F*_HOM_	*F*_ROH_
Abergelle	Abe	Lowland	52	0.369 ± 0.131	0.373 ± 0.119	0.982	0.295 ± 0.008	0.010 ± 0.045	0.023 ± 0.039
Gondar	Gon	Central highland	54	0.376 ± 0.131	0.373 ± 0.118	0.984	0.292 ± 0.011	−0.007 ± 0.019	0.006 ± 0.014
Ambo	Amb	Central highland	71	0.370 ± 0.125	0.375 ± 0.117	0.988	0.296 ± 0.014	0.011 ± 0.050	0.029 ± 0.044
Western Highland/Agew	WeH	Highland	45	0.371 ± 0.134	0.372 ± 0.120	0.988	0.292 ± 0.008	0.001 ± 0.027	0.019 ± 0.022
Western Lowland/Gumez	WeL	Lowland	41	0.370 ± 0.138	0.370 ± 0.122	0.987	0.291 ± 0.019	0.000 ± 0.062	0.021 ± 0.044
Keffa	Kaf	Middle land	36	0.348 ± 0.143	0.361 ± 0.130	0.978	0.289 ± 0.018	0.035 ± 0.105	0.057 ± 0.099
Woyto‐Guji	WoG	Lowland	51	0.375 ± 0.129	0.375 ± 0.117	0.985	0.295 ± 0.009	0.002 ± 0.028	0.011 ± 0.015
Arsi‐Bale	ArB	Highland	46	0.365 ± 0.130	0.374 ± 0.118	0.991	0.297 ± 0.009	0.022 ± 0.056	0.044 ± 0.051
Afar	Afa	Lowland	49	0.382 ± 0.124	0.386 ± 0.110	0.996	0.304 ± 0.009	0.009 ± 0.061	0.028 ± 0.070
Hararghe Highland	HaH	Highland	44	0.379 ± 0.124	0.385 ± 0.109	0.997	0.304 ± 0.015	0.014 ± 0.062	0.043 ± 0.072
Short‐Eared‐Somali	SeS	Lowland	41	0.379 ± 0.127	0.381 ± 0.111	0.995	0.300 ± 0.007	0.006 ± 0.045	0.028 ± 0.051
Long‐Eared‐Somali	LeS	Lowland	47	0.375 ± 0.132	0.374 ± 0.118	0.992	0.294 ± 0.012	−0.002 ± 0.030	0.016 ± 0.024
Nubian	Nub	Lowland	51	0.369 ± 0.115	0.391 ± 0.106	0.994	0.315 ± 0.019	0.054 ± 0.118	0.087 ± 0.116

Note

*N*, H_O_, H_E_, P_N_ and D_ST_, *F*_HOM_ and *F*_ROH_ refer to sample size, observed and expected heterozygosity, proportion of polymorphic SNPs, average pairwise genetic distance, inbreeding coefficients based on excess of homozygosity and runs of homozygosity, respectively.

### 2.2 Data analysis

To estimate genetic diversity, observed (*H*_O_) and expected (*H*_E_) heterozygosity, proportion of polymorphic SNPs (*P*_N_) and average MAF were calculated with PLINK v1.9. The average pairwise genetic distances between individuals within a population were also calculated with PLINK v1.9. Higher values indicate elevated genetic distances and thus low genetic relationships. The average proportion of alleles shared between two individuals was calculated as D_ST_ using PLINK v1.9 with the “–genome” command line:

$$D_{\text{ST}}=\frac{{\text{IBS}}_{2}+\left(0.5\times {\text{IBS}}_{1}\right)}{N}$$

IBS_1_ and IBS_2_ represent the number of loci which share either one or two alleles that are identical by state in pairwise comparisons between individuals, respectively, and *N* is the number of loci tested. The genetic distance between all possible pairwise combinations of individuals was calculated as D = 1−D_ST_.

Detection of ROH was performed by invoking the “*‐‐homozyg*” option in PLINK v1.9. To account for medium marker density, the following set of conditions were imposed: minimum length of ROH = 1 Mb, number of SNPs present in the ROH = 5, number of missing SNP in the ROH = 1, minimum allowed density of SNPs within a run = 1 SNP/100 Kb, number of heterozygous SNPs in each ROH = 1 and maximum gap between consecutive homozygous SNPs = 1 Mb. Descriptive statistics for the total ROH were also calculated, and Pearson's correlation coefficients between average ROH length and the average number of ROH per population were computed with R (https://www.r‐project.org/). The ROH was classified into four length categories: 2, 4, 8 and 16 Mb.

For each population, runs of homozygosity (*F*_ROH_; Kim et al., 2013) and excess of homozygosity (*F*_HOM_; Wright, 1922) statistics were calculated as estimators of genomic inbreeding. *F*_ROH_ was derived for each individual following McQuillan et al. (2008):

$$F_{\text{ROH}}=\frac{\sum_{K}\left(\text{Length}\left({\text{ROH}}_{K}\right)\right)}{L}$$

where the numerator represents the sum of ROH per animal above a certain criteria length and the denominator (L) is the total length of the genome covered by autosomal markers (McQuillan et al., 2008). The *F*_HOM_ statistic was derived using the formulae:

$$F_{\text{HOM}}=\frac{O_{\text{HOM}}‐E_{\text{HOM}}}{1‐E_{\text{HOM}}}$$

O_HOM_ and E_HOM_ represent the observed and expected homozygosity for each population, respectively.

To determine the extent of linkage disequilibrium (LD) between adjacent SNPs, the *r* ^2^ statistic was calculated for each pair of loci using the formulae:

$$r^{2}\left(\text{fA},\text{fB},\text{fAB}\right)=\frac{{\left[f\left(\text{AB}\right)‐f\left(A\right)f\left(B\right)\right]}^{2}}{\text{fA}\left(1‐\text{fA}\right)\text{fB}\left(1‐\text{fB}\right)}$$

where f(AB) is the frequency of haplotypes having allele A at locus 1 and allele B at locus 2 and f(A) and f(B) are the observed allele frequencies at each locus (Hill & Robertson, 1968). For the calculation, we used the “*‐‐ld‐window 9,999 ‐‐ld‐window‐kb 1,000 ‐‐ld‐window‐r^2^ 0*” command line in PLINK v1.9. These settings allowed the analysis of SNPs that were not more than 9,999 SNPs apart, set the window size to 1,000 kb and used all SNPs in the analysis.

The *r* ^2^ values were used to model changes in effective population sizes (*N*_e_) over generations for each population and across the 13 populations using the SNeP tool (Barbato, Orozco‐terWengel, Tapio, & Bruford, 2015). Sved (1971) described the relationship between LD (*r* ^2^), *N*_e_ and c (recombination rate) using the equation:

$$\text{E(}r^{2})=1/\left(\alpha +KN_{e}c\right)+1/n$$

$$N_{e}=1/\left(r^{2}‐1/n\right)\text{kc}‐2/\text{kc}$$

Here, *r* ^2^ is the LD between different markers, *N*_e_ is the effective population size, c is the genetic distance between various markers measured in Morgans, *n* is the chromosome experimental sample size, α is a correction for the occurrence of mutations (Ohta & Kimura, 1971; α = 1 in the absence of mutation and α = 2 if mutation is considered), k = 4 for autosomes and k = 2 for the X chromosome. In contemporary studies, physical distance is used instead of genetic distance to estimate population size. A physical distance of 100 Kb is approximately equivalent to a genetic distance of 0.1 cM (or 1 cM ≈1 Mbp; Dumont & Payseur, 2008). When population size is changing linearly, the expectation of chromosome segment homozygosity is ~1/(4Ntc + 1), where Nt is the population size 1/(2c) generations ago (Hayes, Visscher, McPartlan, & Goddard, 2003). SNPs with a MAF > 0.05 were used to estimate *N*_e_.

The underlying population genetic structure was assessed with principal component analysis (PCA; Jolliffe, 2002), ADMIXTURE v1.3.0 (Alexander, Novembre, & Lange, 2009), TreeMix v1.13 (Pickrell & Pritchard, 2012) and NetView P v0.4.2.5 (Steinig, Neuditschko, Khatkar, Raadsma, & Zenger, 2016). PCA was performed with the “‐‐pca” command line in PLINK v1.9. To investigate past and recent population admixture, the number of genetic clusters inherent in the genomes of the study populations was determined with ADMIXTURE v1.3.0. The number of genetic clusters tested ranged between 2 and 13, and CLUMP v1.1.2 and DISTRUCT v1.1 were used to process and generate the graphics, respectively. To investigate patterns of population splits and cross‐population gene flow, the maximum‐likelihood tree‐based approach incorporating admixture events and implemented in TreeMix v1.13 was used. The phylogenetic tree was built without rooting, and the migration events “m” modelled as edges were added to the phylogeny until the model explained at least 99% of the variance in ancestry. The value of “m” with the highest log‐likelihood following six replicate runs of TreeMix v1.13 was chosen as the optimal.

To reveal fine‐scale population stratification independent of *a priori* ancestry information, network analysis was carried out using the NetView v.0.4.2.5 (Neuditschko, Khatkar, & Raadsma, 2012; Steinig et al., 2016). NetView explores network topologies using a single user‐defined threshold parameter, the number of mutual nearest neighbours (k). Fewer individuals are considered nearest neighbours at small values of k, leading to only genetically more similar individuals being connected and highlighting fine‐scale structure in the data set. We generated a population network based on shared allele distance matrix (1‐identity by state (IBS)) generated with PLINK v.1.9. The network was constructed with the super‐paramagnetic clustering (SPC) algorithm and Sorting Points Into Neighbourhoods (SPIN) software, which computes the maximum number of nearest neighbours for a given individual (Neuditschko et al., 2012; Steinig et al., 2016). The network was visualized and edited in the Cytoscape v.2.8.3 network construction package (Smoot, Ono, Ruscheinski, Wang, & Ideker, 2011). SPC and CYTOSCAPE are implemented in NetView. NetView requires a specification of the maximum number of nearest neighbours (k‐NN) that an individual can have. In this study, the number of k‐NN values tested was between 5 and 120.

The software hapFLK v.1.2 (Fariello, Boitard, Naya, SanCristobal, & Servin, 2013) was used to implement the hapFLK algorithm, which can be applied to un‐phased genotypic data, to detect signatures of selection while accounting for haplotype structure and varying *N*_e_. The implementation required the construction of a neighbour‐joining (NJ) tree using a kinship matrix. Pairwise Reynolds genetic distances (Reynolds, Weir, & Cockerham, 1983) were calculated and converted to a kinship matrix with R scripts provided in the hapFLK webpage (https://forge‐dga.jouy.inra.fr/projects/hapflk). In the construction of the NJ tree, no outgroup population was defined, but the software was prompted to use all populations and the midpoint as outgroup. The kinship matrix captured the population structure, which was used to model covariance matrix of allele frequencies, whereas a multi‐point LD model was used to create haplotype clusters on each chromosome (set to 13 (‐k, 13) per chromosome). This was determined using cross‐validation based estimation in fastPHASE 1.4.0 (Scheet & Stephens, 2006) using the setting ‐KL10 ‐KU20 ‐Ki3. The hapFLK statistic was calculated for each SNP as the average of 20 expected maximization runs fitting the LD model. *P*‐values were then computed based on a chi‐square distribution using a Python script (https://forge‐dga.jouy.inra.fr/document/588). To limit false positives, a q‐value threshold of 0.01 was applied to control for false discovery rate (FDR). Putative selection signatures were defined by the regions with a threshold of *p* < .001.

To further pinpoint loci under selection, we also used the cross‐population extended haplotype homozygosity (XP‐EHH) test (Sabeti et al., 2002, 2007) to make comparisons between the 13 populations. The XP‐EHH assesses haplotype differences between two populations and is designed to detect alleles that have increased in frequency to the point of fixation or near‐fixation in one of the populations (Pickrell et al., 2009; Sabeti et al., 2007). The test uses the integrated EHH (iHH) of a core SNP in two populations, A and B, rather than two alleles in a single population. The unstandardized XP‐EHH statistic is calculated as follows:

$$\text{unstandardized XP}‐\text{EHH}=\ln\left(\text{iHHA}/\text{iHHB}\right)$$

where iHH_A_ and iHH_B_ are the integrated EHH of a given core SNP in population A and B, respectively. A large positive value of XP‐EHH at a locus suggests selection in population A and in the case of a negative value, selection in population B. Here, we used the software developed by Pickrell et al. (2009) to estimate the unstandardized XP‐EHH statistics for all SNPs in all the 13 populations with cross‐population comparison of each population against the other 12. The unstandardized XP‐EHH statistics were standardized using their means and variances in each comparison. Because previous studies showed that the standardized XP‐EHH statistics follow standard normal distribution (Ma, Zhang, Zhang, & Ding, 2014; Sabeti et al., 2007), *P*‐values were estimated using the standard normal distribution. For each cross‐population comparison, we determined the regions under selection based on the threshold *P *< .001.

Using the NCBI map viewer, the regions revealed by hapFLK and XP‐EHH were annotated using the ARS1 release 102 goat reference genome assembly (Bickhart et al., 2017). Functional enrichment and gene ontology (GO) analysis were performed with Panther 14.1 (Mi, Muruganujan, Ebert, Huang, & Thomas, 2019) using *Bos taurus* as the background. We performed text mining with STRING 11.0 (https://string‐db.org/) to identify phenotypes and protein–protein interaction networks associated with the candidate genes.

## 3 RESULTS

Genetic diversity indices (mean ± standard deviation (*SD*); Table 1) that were calculated for each population show that Keffa had the lowest values of *H*_O_ (0.348 ± 0.143), *H*_E_ (0.361 ± 0.130), *P*_N_ (0.978) and *D*_ST_ (0.289 ± 0.018). The highest values were observed in Afar (*H*_O_ = 0.382 ± 0.124), Nubian (*H*_E_ = 0.391 ± 0.106; *D*_ST_ = 0.315 ± 0.019) and Hararghe Highland (*P*_N_ = 0.997). The lowest values of inbreeding were observed in Long‐eared Somali (*F*_HOM_ = −0.002 ± 0.03) and Gondar (*F*_ROH_ = 0.006 ± 0.014), respectively. Overall, Nubian had the highest values of *F*_HOM_ (0.054 ± 0.118) and *F*_ROH_ (0.087 ± 0.116). *F*_HOM_ showed a strong positive correlation (*r* = .978) with *F*_ROH_.

**Figure 1:**
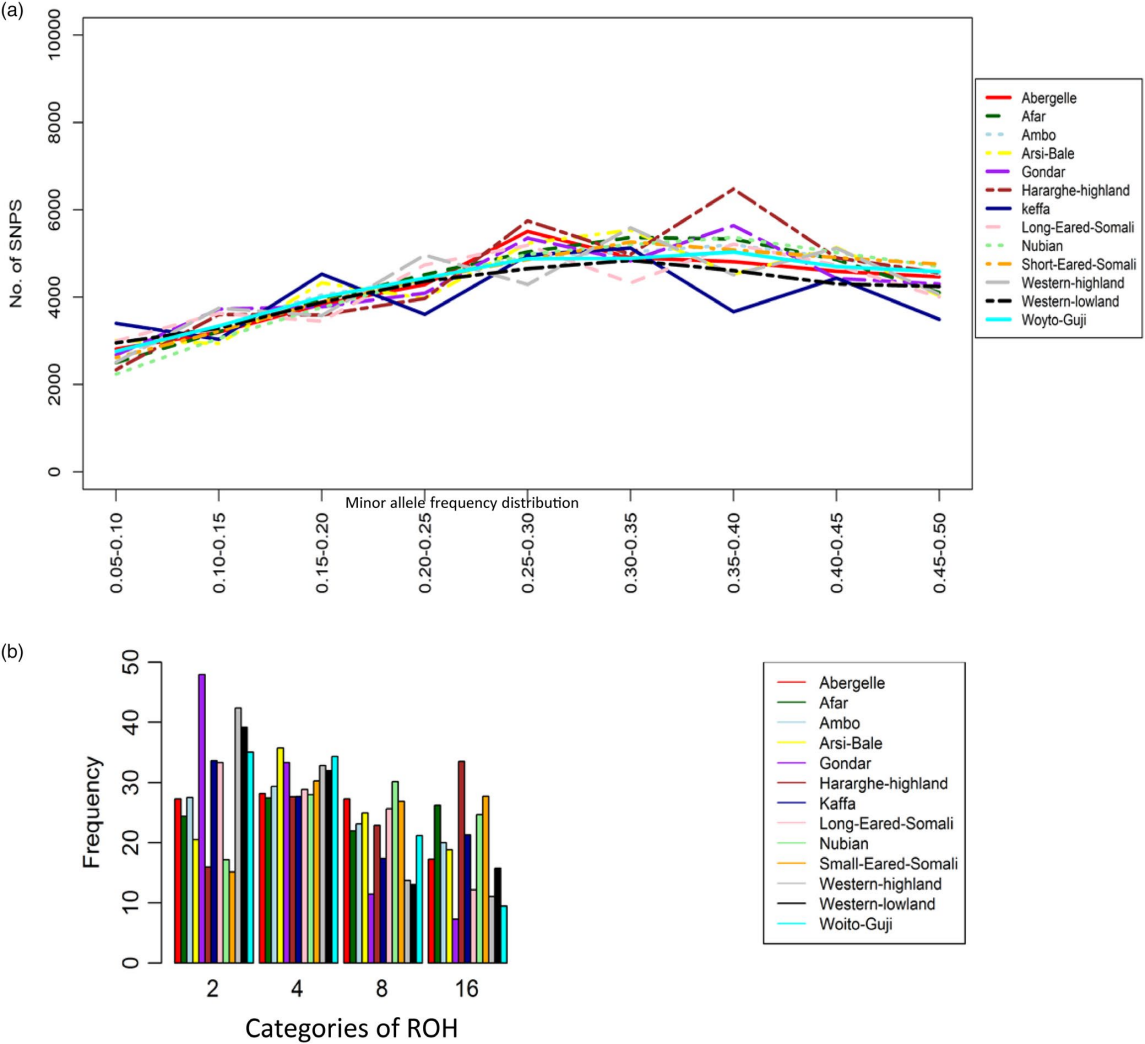
(a) Minor allele frequency (MAF) distribution and (b) Frequency of runs of homozygosity segments (2, 4, 8 and 16 Mb) in each Ethiopian goat population

The highest and lowest proportion of SNPs with low and high MAF, respectively, were observed in Keffa (Figure 1a). Woyto‐Guji and Western lowland/Gumez had the lowest variation in MAF. Figure 1b shows the proportion of ROH for the four genome length categories (2, 4, 8 and 16 Mb). Gondar had the highest proportion of short ROH (2 Mb), followed by Western highland/Agew, Western lowland/Gumez, Woyto‐Guji, Keffa and Long‐eared Somali, respectively. Populations with the highest proportion of long ROH (16 Mb) were Small‐eared Somali, Hararghe Highland and Nubian. Abergelle, Ambo, Afar and Arsi‐Bale had a high proportion of ROH segments of intermediate length.

The average number of ROH segments (mean ± *SD*) per animal ranged from 2.46 ± 2.99 (Gondar) to 16.85 ± 19.84 (Nubian) (Table 2). Woyto‐Guji had the next lowest mean number (3.51 ± 2.99), and Keffa presented the next highest mean number (12.13 ± 12.67). The maximum number of ROH segments ranged from 14 (Woyto‐Guji) to 70 (Nubian). Keffa presented the next highest maximum number (44) of ROH segments. The average length of the genome comprising of ROH segments ranged from 4,554.92 ± 2,240.02 Kb (Gondar) to 10,072.83 ± 8,267.14 Kb (Short‐eared Somali). The shortest and longest lengths of the genome comprising of ROH segments were observed in Hararghe Highland (2,236.95) and Short‐eared Somali (40,669.67), respectively. The average number of SNPs within an ROH segment was lowest in Gondar (84.61 ± 40.18) and highest in Short‐eared Somali (187.53 ± 151.62). This presented the minimum and maximum values of 40 SNPs in Afar and 749 SNPs in Short‐eared Somali, respectively. The average SNP density (SNPs per Kb) was similar across the 13 populations (53–54 SNPs/Kb), although the range was large. The proportion of homozygous sites averaged 99% across all populations (Table 2).

**Table 2:** Descriptive statistics for runs of homozygosity (ROH) segments within each of the 13 Ethiopian indigenous goat populations

	No of ROH segments		Size (Kb)		No. of SNP		SNP Density (SNP/Kb)		Prop. of homozygous
	Mean ± *SD*	Max	Mean ± *SD*	Range	Mean ± *SD*	Range	Mean ± *SD*	Range	Mean ± *SD*
Abergelle	5.50 ± 7.45	35	7,152.31 ± 4,247.39	2,420.37–16840.43	132.46 ± 76.94	48–303	53.64 ± 4.04	42.48–62.34	0.99 ± 0.01
Gondar	2.46 ± 2.99	16	4,554.92 ± 2,240.02	2,438.59–13010.61	84.62 ± 40.18	49–241	53.58 ± 3.33	47.35–61.09	0.99 ± 0.01
Ambo	6.64 ± 7.29	33	7,031.41 ± 4,647.14	2,324.55–21353.34	131.22 ± 84.69	48–391	53.16 ± 3.10	43.04–63.05	0.99 ± 0.01
WH/Agew	6.09 ± 4.83	20	5,962.93 ± 3,008.47	2,385.04–14184.03	111.34 ± 54.85	48–263	53.19 ± 2.83	45.53–60.64	0.99 ± 0.01
WL/Gumez	5.69 ± 6.25	38	6,386.99 ± 3,944.45	2,247.98–19014.48	118.72 ± 71.91	52–348	53.44 ± 3.17	43.23–59.64	0.99 ± 0.01
Keffa	12.14 ± 12.67	44	6,881.78 ± 5,043.47	2,822.41–19047.42	128.80 ± 93.20	55–353	53.37 ± 2.04	49.78–57.66	0.99 ± 0.01
WoG	3.51 ± 2.99	14	6,435.37 ± 3,240.10	2,368.95–13882.20	118.09 ± 60.34	50–271	54.61 ± 3.52	47.38–64.31	0.99 ± 0.01
Arsi‐Bale	10.12 ± 9.88	34	8,414.97 ± 3,762.29	2,579.62–16555.66	156.59 ± 68.49	53–304	53.63 ± 2.02	48.67–60.36	0.99 ± 0.01
Afar	5.12 ± 7.95	37	6,635.72 ± 5,722.43	2,340.03–26593.98	123.54 ± 106.88	40–489	53.82 ± 3.31	47.79–64.60	0.99 ± 0.01
HaH	7.23 ± 9.68	33	8,217.44 ± 6,080.78	2,236.95–23376.38	162.93 ± 110.61	49–415	53.24 ± 2.76	44.73–57.04	0.99 ± 0.01
SeS	5.41 ± 6.19	28	10,072.83 ± 8,267.15	2,893.13–40669.67	187.53 ± 151.63	54–749	53.58 ± 2.98	48.39–64.00	0.99 ± 0.01
LeS	4.59 ± 5.01	17	6,207.37 ± 3,281.42	2,474.84–12859.00	114.78 ± 60.63	51–241	54.20 ± 3.43	45.96–62.83	0.99 ± 0.01
Nubian	16.85 ± 19.84	70	9,669.93 ± 5,017.69	2,839.61–21506.99	179.99 ± 91.74	55–396	53.63 ± 2.70	44.62–60.09	0.99 ± 0.01

Abbreviations: WH, Western highland; WL, Western lowland; WoG, Woyto‐Guji; HaH, Hararghe Highland; SeS, Small‐eared Somali; Les, Long‐eared Somali.

**Figure 2:**
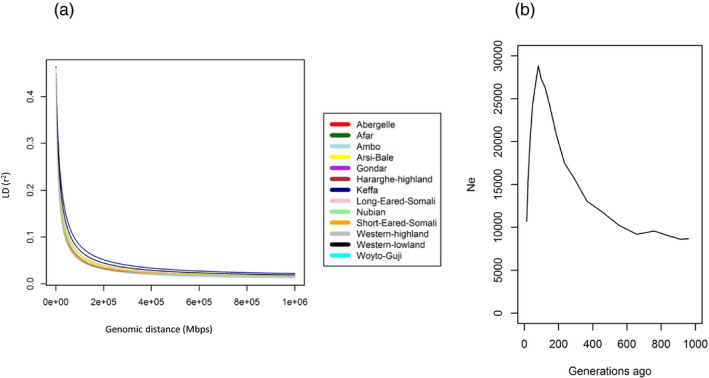
(a) Trend in LD decay over genomic distances for each population and (b) The trend in composite effective population size modelled across the 13 Ethiopian goat population

The average *r* ^2^ value (Table 3) ranged from .028 (Woyto‐Guji) to .051 (Keffa). The trends in LD decay over genomic distances reveal high LD over short distances, which decays rapidly with distance (Figure 2a). LD decays more rapidly in Woyto‐Guji but slower in Keffa, Western highland/Agew and Western lowland/Gumez.

**Table 3:** Average linkage disequilibrium (*r* ^2^), effective population size at 1,000 and 13 generations

Goat populations	*r* ^2^	*N*_e_ (1,000 generations ago)	*N*_e_ (13 generations ago)
Abergelle	.032	7,317	2,778
Gondar	.031	6,274	2,899
Ambo	.029	6,698	1,223
WH/Agew	.039	7,398	1644
WL/Gumez	.045	6,233	719
Keffa	.051	5,408	1,029
Woyto‐Guji	.028	6,994	5,390
Arsi‐Bale	.038	6,342	1,355
Afar	.031	7,302	3,296
Hararghe Highland	.031	7,274	5,401
Short‐eared Somali	.033	7,229	3,988
Long‐eared Somali	.035	6,795	1,491
Nubian	.036	7,408	743

Abbreviations: WH, Western highland; WL, Western lowland.

One thousand (1,000) generations ago, Nubian and Keffa had the highest and lowest *N*_e_, respectively (Table 3). Thirteen (13) generations ago, the lowest *N*_e_ was observed in Gumez and highest in Hararghe Highland. The overall composite trend over the past 1,000 generations (Figure 2b) reveals a gradual increase in *N*_e_ to about 600 generations followed by a rapid increase to about 150 generations, and then a sharp decline to present time. Each population, however, showed contrasting trends (Figure S1). A trend similar to the composite characterizes Woyto‐Guji, Short‐eared Somali and Hararghe Highland. A continuous and gradual decline characterizes Arsi‐Bale, Keffa, Western lowland/Gumez, Western highland/Agew and Nubian. The *N*_e_ for Abergelle, Gondar, Ambo and Long‐eared Somali increases gradually to 200–300 generations ago after which it declines rapidly to present time. The *N*_e_ of the Afar population increases gradually to about 300 generations ago, stabilizes to 150 generations and then declines rapidly to present time.

**Figure 3:**
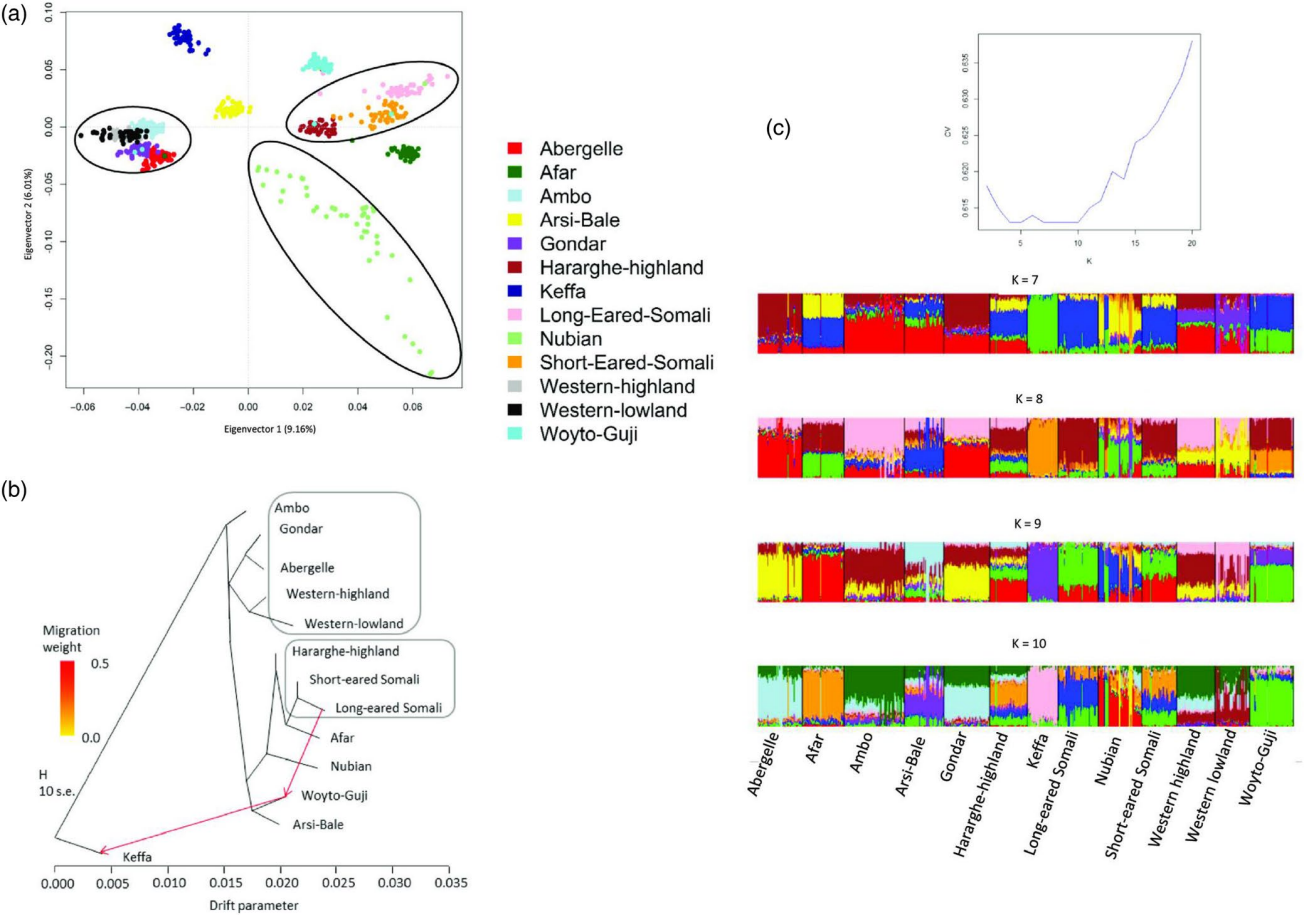
(a) Principal component analysis plot. (b) Tree Mix phylogenetic tree. (c) Plots of CV error against *K* and ADMIXTURE displays for 7 ≤ *K* ≤ 10

PCA projected seven genetic clusters (Figure 3a). Cluster 1 comprised Abergelle, Ambo, Gondar, Western highland/Agew and Western lowland/Gumez. Cluster 2 comprised Hararghe Highland, and Long‐ and Short‐eared Somali. Clusters 3, 4, 5, 6 and 7 comprised, respectively, Afar, Arsi‐Bale, Keffa, Nubian and Woyto‐Guji, which were depicted as distinct genetic entities. In agreement with the high *D*_ST_ value, Nubian individuals spread out across the fourth quadrant of the PCA suggesting high intra‐population variation. TreeMix (Figure 3b) replicated the PCA clusters and showed two gene flow events, from Long‐eared Somali to Woyto‐Guji and from Woyto‐Guji to Keffa.

The optimal value of *K* following ADMIXTURE analysis could not be determined with certainty. At the first instance, the CV error was lowest at 4 ≤ *K* ≤ 5. It then increased slightly at *K* = 6 before declining to the same lowest value observed at 4 ≤ *K* ≤ 5, at *K* = 7, 8, 9 and 10 (Figure 3c inset). If the increase in CV error at *K* = 6 reflects lack of stability in allocating the genetic backgrounds, then the values of *K* at 7, 8, 9 and 10 may explain the variation in the data set. Taking *K* = 7 as the most optimal, it supports the PCA and TreeMix but with higher resolution. At this *K*‐value, Keffa is the only population with one uniform genetic background which is shared with Woyto‐Guji. The other major genetic backgrounds are observed in Abergelle, Ambo, Long‐eared Somali, Nubian and Gumez. The seventh genetic background is unique to a few individuals of Nubian. Other than these individuals of Nubian and Keffa, the other populations exhibit variable proportions of genome admixture of at least two genetic backgrounds. Although this admixture pattern is repeated at 8 ≤ *K* ≤ 10, the genomes of Afar and Woyto‐Guji show gradual reduction in admixture while the opposite is observed for Arsi‐Bale and Nubian. By revealing a common genome background between Long‐eared Somali and Woyto‐Guji and between Woyto‐Guji and Keffa at 7 ≤ *K* ≤ 10, ADMIXTURE corroborates TreeMix that showed migration events between these populations.

**Figure 4:**
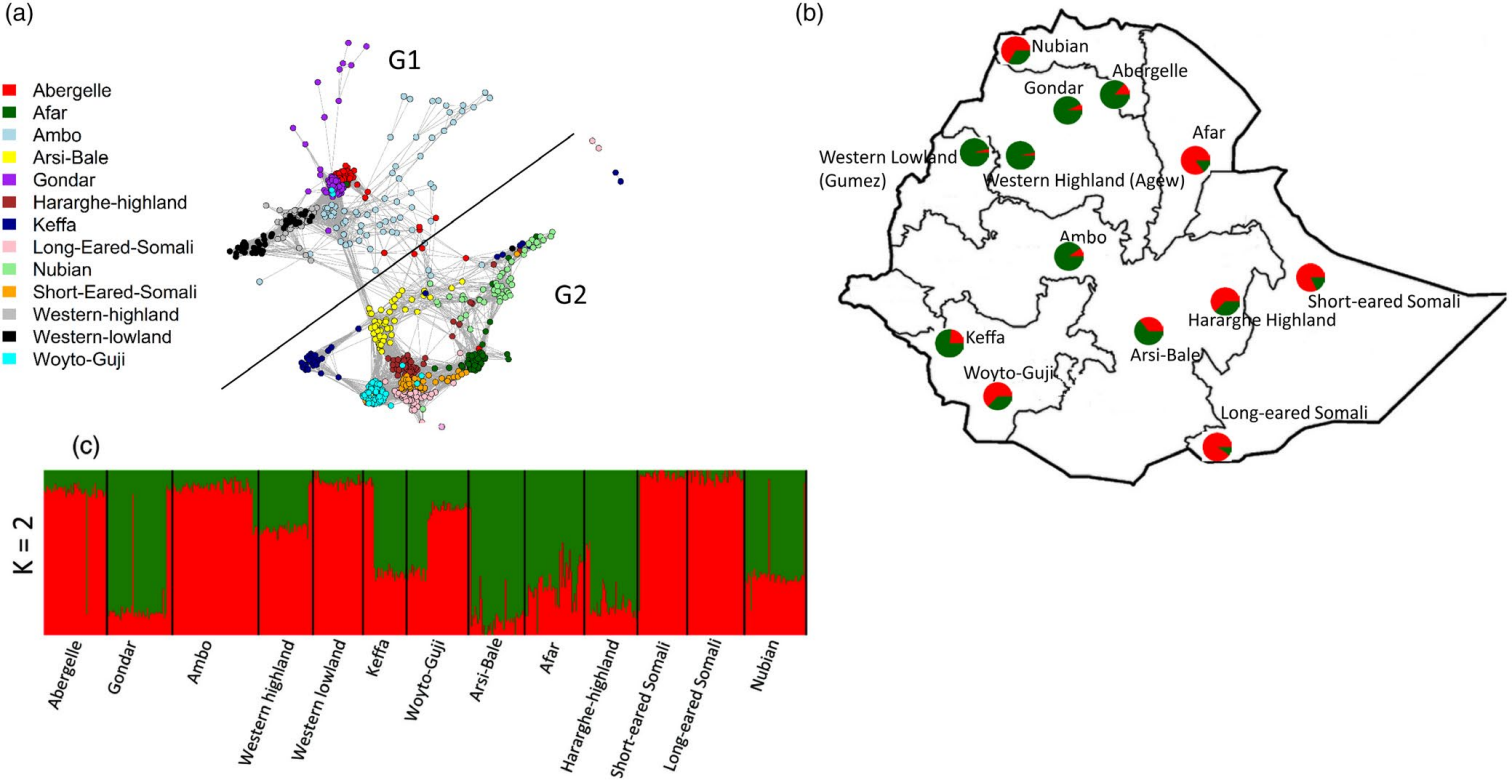
(a) NetView P plot for K‐NN = 75 showing clustering of the 13 populations into two groups. (b) Geographic sampling regions in Ethiopia together with pie charts showing ancestry proportions for *K* = 2 generated in ADMIXTURE. (c) ADMIXTURE bar plot showing proportion of ancestries for *K* = 2

NetView (Figure S2) showed that from 75 ≤ k‐NN ≤ 120, only four individuals, two each of Keffa and Long‐eared Somali, remained unassigned. The major clusters became evident, at the first instance, from k‐NN = 55, when the 13 populations appeared to separate into two broad groups, hereby designated as G1 and G2 (Figure 4a). G1 comprised Abergelle, Ambo, Gondar, Western highland/Agew and Western lowland/Gumez. G2 comprised Afar, Arsi‐Bale, Hararghe Highland, Keffa, Long‐eared Somali, Nubian, Short‐eared Somali and Woyto‐Guji. These two groups are consistently retained up to k‐NN = 120. This prompted us to look at the results of ADMIXTURE at *K* = 2 (Figure 4b,c). As expected, it revealed two genome backgrounds, hereby named GB1 and GB2. GB1 occurs at a frequency of >70% in Abergelle, Ambo, Western highland/Agew, Western lowland/Gumez, and Short‐ and Long‐eared Somali. Abergelle, Ambo, Western highland/Agew and Western lowland/Gumez comprise G1 of NetView. GB2 is found at a frequency of >70% in Arsi‐Bale, Afar, Gondar, Hararghe Highland and Nubian, all the populations found in G2 of NetView except Gondar. Long‐ and Short‐eared Somali occur in G2 of NetView but in GB1 of ADMIXTURE. Although Keffa and Woyto‐Guji occur in G2, in ADMIXTURE they show almost an equal proportion of GB1 and GB2 in their genomes. The minor discordance between NetView and ADMIXTURE could be due to differences in the way they are structured to allocate populations into clusters. Despite this minor difference, the NetView results led us to hypothesize that the analysis is revealing, possibly two ancestral genomes that could have arrived with the initial introduction of goats in the Horn of Africa.

**Figure 5:**
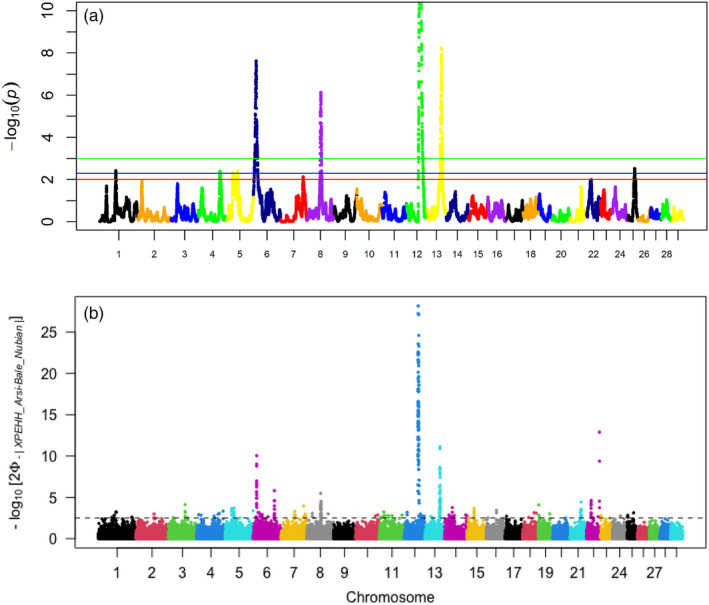
Genome‐wide hapFLK statistics between Arsi‐Bale and Nubian goat populations. Green and red lines correspond to *p* < .005 and <.001, respectively

Seventy‐eight pairwise comparisons were performed with hapFLK v1.2 software and XP‐EHH test, assuming that each population was adapted to specific circumstances in its home range. Although the two approaches identified multiple regions under selection (Figures S3 and S4), here we focused only on the regions that overlapped between the two approaches. The regions revealed by XP‐EHH fell within the boundaries of the ones identified by hapFLK. The most significant and consistent signals were revealed in pairwise comparisons involving Arsi‐Bale and Nubian with the other 11 populations (Figures S3 and S4). The ones involving Arsi‐Bale were on chromosome (CHI) 6 (8,533,828 7,357,213 bp; average size: 3,621,429 ± 2,264,393 bp) and CHI12 (53,982,423–62,977,761 bp; average size: 6,978,324 ± 952,467.8 bp). The ones involving Nubian were on CHI8 (54,717,960–63,857,851 bp; average size: 4,912,265 ± 1,773,863 bp) and CHI13 (49,975,695–67,760,577 bp; average size: 10,147,644 ± 2,916,617 bp). Comparative analysis between Arsi‐Bale and Nubian revealed the four putative signatures (Figure 5a,b), suggesting they are specific to these two populations. Arsi‐Bale goats carry dense hairy coats and reside at an altitude of ≥4,000 m above sea level (www.dagris.info/). Nubian goats have long‐haired coats and reside at low‐altitude drylands in north‐western Ethiopia, northern Sudan and western Eritrea (www.dagris.info/). High altitudes present a hypoxic cold environment. Low‐altitude drylands present an environment characterized by grazing stress. Grazing stress encompasses complex interacting biophysical stressors including heat, physical exhaustion, direct solar radiation and unavailability of feed (quality and quantity) and water. The selection signatures observed in Arsi‐Bale and Nubian led us to hypothesize that putative selection may have resulted in adaptive and phenotypic divergence in the two populations.

We annotated the four candidate regions based on the ARS1 release 102 goat reference genome assembly. We found a total of 123 (Arsi‐Bale = 29; Nubian = 94) unannotated genes, that is prefixed with “LOC/ENSCHIG” in the candidate regions. Although potentially under selection, these unannotated genes were not included in the functional enrichment and ontology analysis. In total, 20 (CHI6) and 45 (CHI12) genes were present in the Arsi‐Bale regions, while 62 (CHI8) and 244 (CHI13) were observed in the Nubian regions (Table S1). Functional enrichment and GO analysis were performed separately for each population‐specific gene sets using Panther and STRING. The 65 Arsi‐Bale and 306 Nubian candidate genes showed greater enrichment in biological processes relating to cellular processes (GO: 0009987), metabolic processes (GO: 0008152) and biological regulation (GO: 0065007). Text mining was performed with STRING to determine gene functions from published literature. It identified that several of the candidate genes are linked with traits of adaptive, economic and functional significance. Some genes such as *CDX2*, *NBEA* and *PITX* (Arsi‐Bale) and *PROCR*, *CTCFL*, *NNAT* and *PDYN* (Nubian) are involved in multiple functions.

## 4 DISCUSSION

### 4.1 Genetic variation and marker polymorphisms

We present findings from an analysis of genome diversity, structure and dynamics of indigenous goats in Ethiopia, at the route of one of the most ancient gateways of domesticates into Africa. Ethiopia is characterized by ancient and modern human ethnic diversity that has long been associated with livestock husbandry and diverse agro‐eco‐climates, which may have influenced the genome architecture of indigenous livestock. The 13 study populations retained high levels of genome diversity (mean *H_O_* and *H_E_* above 0.348) and within population genetic variation (D_ST_ > 0.289). Although these values are high, they are close to those reported in other indigenous goats (Berihulay et al., 2019; Kim et al., 2016; Manunza et al., 2016; Mdladla, Dzomba, Huson, & Muchadeyi, 2016; Nicoloso et al., 2015; Onzima et al., 2018). Keffa was the least and Nubian was the most diverse, population. Extensive genome admixture incorporating at least two genetic backgrounds at 7 ≤ *K ≤* 10 was observed in all but Keffa. This admixture could explain the high diversity and genome variability in the Ethiopian indigenous goats. It is the result of past and modern‐day socio‐economic and cultural exchanges and natural selection which retains genetic variation.

### 4.2 ROH and inbreeding

Genomic inbreeding coefficients, such as F_ROH_ and F_HOM_, are more accurate at estimating autozygosity and detecting past and recent inbreeding compared to estimates derived from pedigree data (Curik, Ferenèakoviæ, & Sölkner, 2014; Ferenčaković et al., 2013; Knief, Kempenaers, & Forstmeier, 2017). Furthermore, in the absence of genealogical information, molecular data can be used to infer population history and inbreeding (Kim, Sonstegard, Van Tassell, Wiggans, & Rothschild, 2015; Zavarez et al., 2015). The latter is important especially for African indigenous livestock that most often lack written pedigree records. The strong and positive correlation (*r* = .978) between F_ROH_ and F_HOM_ observed in our study corroborates previous findings in pigs (Zhang et al., 2014) and cattle (Mastrangelo et al., 2016). It confirms that the extent of a genome under ROH can be used to predict the proportion of the genome that is identical by descent (IBD). A high and positive correlation between F_ROH_ and conventional pedigree‐based estimates of inbreeding (F_PED_) has been reported (Ferenčaković et al., 2013; Martikainen, Tyrisevä, Matilainen, Pösö, & Uimari, 2017; Purfield, Berry, McParland, & Bradley, 2012) confirming F_ROH_ as an appropriate estimator of IBD alleles. The importance of understanding and quantifying genome‐wide autozygosity has also been highlighted through correlations of F_ROH_ with inbreeding depression for a range of production (Bjelland, Weigel, Vukasinovic, & Nkrumah, 2013; Kim et al., 2015; Pryce, Haile‐Mariam, Goddard, & Hayes, 2014) and fertility (Kim et al., 2015; Martikainen et al., 2017) traits. In this study, F_ROH_ and F_HOM_ revealed low levels of genomic inbreeding in Ethiopian indigenous goats, results that are consistent with findings on Egyptian Barki (Kim et al., 2016) and Ugandan goats (Onzima et al., 2018). Colli et al. (2018) suggested that significant introgression of other breeds in African goats explains their low inbreeding. We however suggest that low inbreeding in African goats is the result of extensive outcrossing within and between diverse populations and individuals considering that these flocks are communally grazed and watered, and mating is mostly uncontrolled.

### 4.3 Demographic history and dynamics

Genome‐wide ROH, LD and *N*_e_ are valuable sources of information on how population structure, demography and management evolve over time (Ceballos et al., 2018; Kirin et al., 2010). Generally, short ROH is most likely correlated with ancestral inheritance, ancient bottlenecks or consanguinity, whereas long ROH is associated with recent inbreeding (Browning & Browning, 2013; Purfield et al., 2012). The distribution of ROH can also provide information on specific selection events. The distribution of the sizes of ROH segments varied across the 13 study populations, with six reporting high proportion of short (<2 Mb) ROH segments, three with a high proportion of long (≥8–16 Mb) ROH segments and four with intermediate (4 Mb) ROH segments. The variable distribution of ROH lengths with frequent short and intermediate length ROH segments has been observed in other goats (Brito et al., 2017; Kim et al., 2016; Onzima et al., 2018), sheep (Purfield, McParland, Wall, & Berry, 2017), cattle (Mastrangelo et al., 2016) and pigs (Bosse et al., 2012). The high proportion of short ROH observed in Gondar, Western highland/Agew, Western lowland/Gumez, Woyto‐Guji, Keffa and Long‐eared Somali may indicate more ancient inbreeding and/or bottleneck. The high number of long ROH in the Small‐eared Somali, Hararghe Highland and Nubian suggests a large effect of recent inbreeding. However, for all the 13 populations analysed in this study, low levels of genomic inbreeding were observed and thus ancient and contemporary bottleneck may explain our results, a possibility that we do not favour. Although selection coupled with a smaller *N*_e_ can account for differences in ROH lengths, we also do not favour this as a possible explanation of our results as artificial selection for any trait is either weak or absent among Ethiopian goats. We suggest that the high proportion of short ROH segments in Gondar, Western highland/Agew, Western lowland/Gumez, Woyto‐Guji, Keffa and Long‐eared Somali may indicate an initial small founder population, while the long ROH segments in Small‐eared Somali, Hararghe Highland and Nubian were possibly derived from large founding flocks.

Although we observed an overall decrease in *N*_e_ from 1,000 to 13 generations ago, each population had a unique trend (Figure S1). Assuming a generation time of 3 years for traditionally managed indigenous goats, it can be inferred from the composite plot that the gradual increase in *N_e_* begun around 1,860 years ago (ya) (620 generations). It was followed by a rapid increase starting around 1,140 ya (380 generations) and then by a sharp decline from around 480 ya (160 generations) that persists until now (Figure 2b). Within the estimated time frame (240–1140 ya), three exceptionally favourable climatic periods, corresponding to the African Humid Period (AHP) (Wright, 2017), interspersed with short dry spells, were experienced in eastern Africa (Verschuren, 2007; Verschuren et al., 2000). Indigenous goats seem to have thrived when conditions were favourable but drastically shrunk during the subsequent drought (Verschuren, 2007; Verschuren et al., 2000) or following the abrupt or gradual termination of the AHP. Each population, however, seems to have responded differently to the climatic events as deduced from the individual trends (Figure S1). It suggests the historical demographic dynamics of the indigenous goats may have been more complex than is reflected by the composite trend thus supporting further investigation possibly using the PSMC algorithm (Nadachowska‐Brzyska, Burri, Smeds, & Ellegren, 2016) and full genome sequences. The PSMC capitalizes on the combined pattern of the distributions of the time to the most recent common ancestor (TMRCA) between two alleles in an individual at large number of loci spread across the genome. It therefore gives more detailed information on historical‐ancient *N*_e_ dynamics. Full genome sequences offer the advantage of sampling large numbers of unlinked loci across the genome. This may be necessary to accurately estimate population genetic parameters and determine the timing of demographic events.

### 4.4 Past and recent population genetic structure

The use of several methods to investigate population structure allowed us to reveal and describe past and recent population genetic structure in indigenous goats in Ethiopia and by extension sub‐Saharan Africa. NetView revealed two broad genetic groups lacking a clear phylogeographic structure that corresponded slightly to the genetic backgrounds revealed by ADMIXTURE at *K* = 2. These two genetic groups may possibly represent two deep ancient ancestries that arrived with the first introduction of goats in eastern Africa. It mirrors findings of mitochondrial DNA which identified two haplogroups that also lacked a clear phylogeographic structure in Ethiopian (Tarekegn et al., 2018), Kenyan (Kibegwa et al., 2015), Sudanese (Sanhory et al., 2014) and Egyptian (Naderi et al., 2008) indigenous goats. With the current data set, we nevertheless cannot determine the origin and route of entry and dispersal of the two deep ancestries and whether they arrived together or independently. However, their occurrence in all the 13 populations, albeit at different frequencies, points to a relatively early dispersal in the region, most likely facilitated by human socio‐cultural and trade interactions as inferred from anthropologic, linguistic and human genetic studies (Pagani et al., 2012).

Cryptic population genetic structure, represented by seven genetic groups, was revealed by PCA and TreeMix. These results were supported by the output of ADMIXTURE: the optimal number of clusters was K = 7. Except Keffa, the genomes of the other populations are a mosaic of at least two genetic backgrounds. This pattern was repeated at *K* ≥ 7 except for Abergelle, Woyto‐Guji and Afar which showed one predominant genetic background. How these seven backgrounds evolved from the two ancestries remains a subject of speculation. Geo‐specific local founder events accentuated by genetic drift arising from past reproductive isolation could be a possible source of the observed evolutionary hepta‐groupings. With extensive human migrations across Ethiopia dating back to the 16th century (Habitamu, 2014; Yelma, 1966), it is possible that human cultural and socio‐economic interactions dispersed the seven backgrounds across the country resulting in admixed genomes. These results are similar to the observation of similar levels of genome admixture in Ethiopian cattle (Dadi et al., 2008), sheep (Edea, Dessie, Dadi, Do, & Kim, 2017) and humans (Pagani et al., 2012; Plaster, 2011) and in other African livestock (Benjelloun et al., 2015; Mbole‐Kariuki et al., 2014; Mwacharo et al., 2017), and are explained by the fact that the three livestock species were and still are often kept together.

The unique genetic background in Keffa goats at *K* ≥ 7 is noteworthy. This population is found exclusively in the Keffa region of south‐western Ethiopia, which is also the home tract of Sheko cattle. Among Ethiopian cattle, Dadi et al. (2008) observed a homogeneous and unique genetic background in the Sheko. The Keffa region is tsetse‐infested, and trypanosomosis, a potential driver of selection in African cattle (Smetko et al., 2015), is endemic. Sheko cattle are reported to have some degree of trypanotolerance (Lemecha et al., 2006; Mekonnen, Gültas, Effa, Hanotte, & Schmitt, 2019). During informal conversations with Keffa goat owners, they indicated that their goats also exhibit a degree of trypanotolerance. If confirmed, it would explain the genome uniformity in the two species, the result of natural selection for trypanotolerance. Trypanosomosis acts as a natural barrier for the dispersal of other goat and cattle populations in the Keffa region, and until recently when control measures against the disease have been enhanced and the ecology drastically modified for human activities and increased settlements, reproductively isolated the Keffa goats and Sheko cattle. The correlation between the uniform genetic background and reduced susceptibility to trypanosomosis in the two species needs to be confirmed.

### 4.5 Genome‐wide signatures of selection

Four strong selection signatures were identified, two each in Arsi‐Bale and Nubian goats. Despite the observation of a unique genetic background in Keffa that we suggest could be the outcome of trypanotolerance, no selection signatures were observed in this population. This was rather surprising and difficult to explain but may suggest that reproductive isolation and the resulting genome autozygosity could be the cause. We attribute the four strong selection signatures to differential adaptation to contrasting environments namely high altitude and the associated low temperatures experienced by the Arsi‐Bale population, and low dry altitude and high temperatures experienced by the Nubian population. One Arsi‐Bale candidate region spanned *ALOX5AP* a gene that was identified in sheep as a potential candidate for climate‐mediated adaptation (Lv et al., 2014). In humans, a mutation in *ALOX5AP* was associated with lung function (Ro et al., 2012). Given the high altitude and restricted oxygen concentration in the Arsi‐Bale mountains, *ALOX5AP* may play a role in adaptation to high altitude, especially in so far as respiration is concerned. Several candidate genes with critical roles in maintaining genome integrity through DNA repair processes (*BRCA2*, *PDS5B*, *RAD51*) (Couturier et al., 2016; Prakash, Zhang, Feng, & Jasin, 2015) overlapped with the Arsi‐Bale candidate genomic regions. In the tropics, high‐altitude regions receive much higher annual levels of UV radiation. Therefore, maintaining genome integrity against possible DNA damage induced by ionizing radiation is essential. Other candidate genes found in selective sweeps included *KATNAI1*, *FRY* and *RXFP2*. *KATNAI1* has been associated with variation in fibre diameter in domestic sheep breeds (Seroussi, Rosov, Shirak, Lam, & Gootwin, 2017; Zhang et al., 2013). The *FRY* has been linked with variations in coat pigmentation in many sheep breeds (Garcia‐Gamez et al., 2011; Seroussi et al., 2017; Wei et al., 2015; Zhang et al., 2013) and is also involved in growing wing hairs and bristles in Drosophila (Cong et al., 2001; Fang, Lu, Emoto, & Adler, 2010; He, Fang, Emoto, Jan, & Adier, 2005). Mutations in *RXFP2* were linked to horn type and development in sheep (Johnston et al., 2011; Wang, Zhou, Li, Zhao, & Chen, 2014), reproductive success and survival in Soay sheep (Johnston et al., 2013), and the genomic region spanning *RXFP2* was also identified to be under selection in Creole (Gautier & Naves, 2011) and West African Borgou cattle (Flori et al., 2014). We also observed genes (*STARD13*/*CCNA1*, *FLT1*, *DCLK1*, *NBEA*) that are reported to be associated with female reproduction in bovines (Kfir et al., 2018) and chicken (Shen et al., 2017; Sun et al., 2015). The *PITX2* and *CDX2* have been associated with puberty in cattle (Cánovas et al., 2014) and embryogenesis in the mouse (Lu et al., 2018). Evidence drawn from animal experiments suggests that conditions in high‐altitude environments and prolonged exposure to altitude‐initiated stress, including cold and hypoxia, can have direct negative effects on reproductive function (Heath & Williams, 1989). The exposure can have significant negative effects on female reproduction through reduction in fertility, increasing foetal loss and/or reducing fecundability (Adekilekun, Aboua, Oyeyipo, & Oguntibeju, 2019).

The Nubian is a goat breed that is adapted to arid environments. It is therefore not surprising that hapFLK and XP‐EHH identified selective sweeps overlying regions that spanned genes associated with adaptation to environmental stress (*PCK1*, *CTCFL*, *SPO11*, *BMP7*), oxidative stress (*PLAGL2*, *SRXN1*), adaptation to different ecological environments (*ZBTB46*, *ARFRP1*, *STMN3*, *GMEB2*), and mitochondrial homeostasis (*TDRD7*) including classical innate immunity and fever induction (*HCK*, *PROCR*, *CTSZ*) (Table S2). Coat and skin colour are an integral part of adaptation. The coat of Nubian goats is light coloured and most often white. In one of the candidate regions, there were two genes that have been associated with colouration (*GNE*, *EDN3*). Surprisingly, we observed genes that have been associated with dairy (*PROCR*, *AURKA*, *FOXS1*, *CD72*, *AVP, PXMP4*, *PIGU*, *ZNF341*, *NCOA6*, *ACSS2*, *E2F1*), beef (*MYLK2*, *MYL9*, *MYH7B*, *TNFRSF6B*, *PROCR*, *ERGK3*), and reproduction (*CTCFL*, *SPO11*, *RBM38*, *PMEPA1*, *CTNNBL1*, *NNAT*, *BLCAP*, *CPNE1*, *SPAG4*, *CTCFL*, *TGFBR1*) traits (Table S2). With candidate regions overlapping these genes our results demonstrate the potential of the Nubian to be used in breeding programmes to increase production to meet future demand for proteins of animal origin under unpredictable climatic conditions.

## 5 CONCLUSION

The genetic history of African indigenous goats is complex. It has obviously been closely intertwined with the history of local human communities. The need to adapt to diverse African environments may also have shaped present‐day African goat genomes. Here, we have provided insights that improve our understanding of the genomic landscape and demographic history of sub‐Sahara African indigenous goats in Ethiopia. Our results provide a foundation to formulate and test biological hypotheses relating to population demographic profiles and genome dynamics in African livestock. Further studies are needed to refine and confirm our interpretations and the proposed hypotheses. These include among others, the association between the uniform genetic background in Keffa and reduced susceptibility to trypanosomosis, the origin and route of entry and dispersal of the two deep ancestries into the region, and the suggestion that the historical demographic dynamics of the indigenous goats may be more complex than is reflected by the composite trend.

## CONFLICT OF INTEREST

The authors declare no conflict of interest regarding this manuscript.

## Supporting information

Fig S1‐S2

Fig S3

Fig S4

Tables S1‐S2

## Data Availability

The data used here will be made available upon request.
