# Supplementary material for: Ethiopian indigenous goats offer insights into past and recent demographic dynamics and local adaptation in sub‐Saharan African goats
Source: Evol Appl. 2021 Jun 15;14(7):1716–31. doi: 10.1111/eva.13118 (PMC8287980; doi:10.1111/eva.13118)
Supplement: Supplementary file 1 — Fig S1‐S2 [file EVA-14-1716-s003.pptx]

## Slide 1
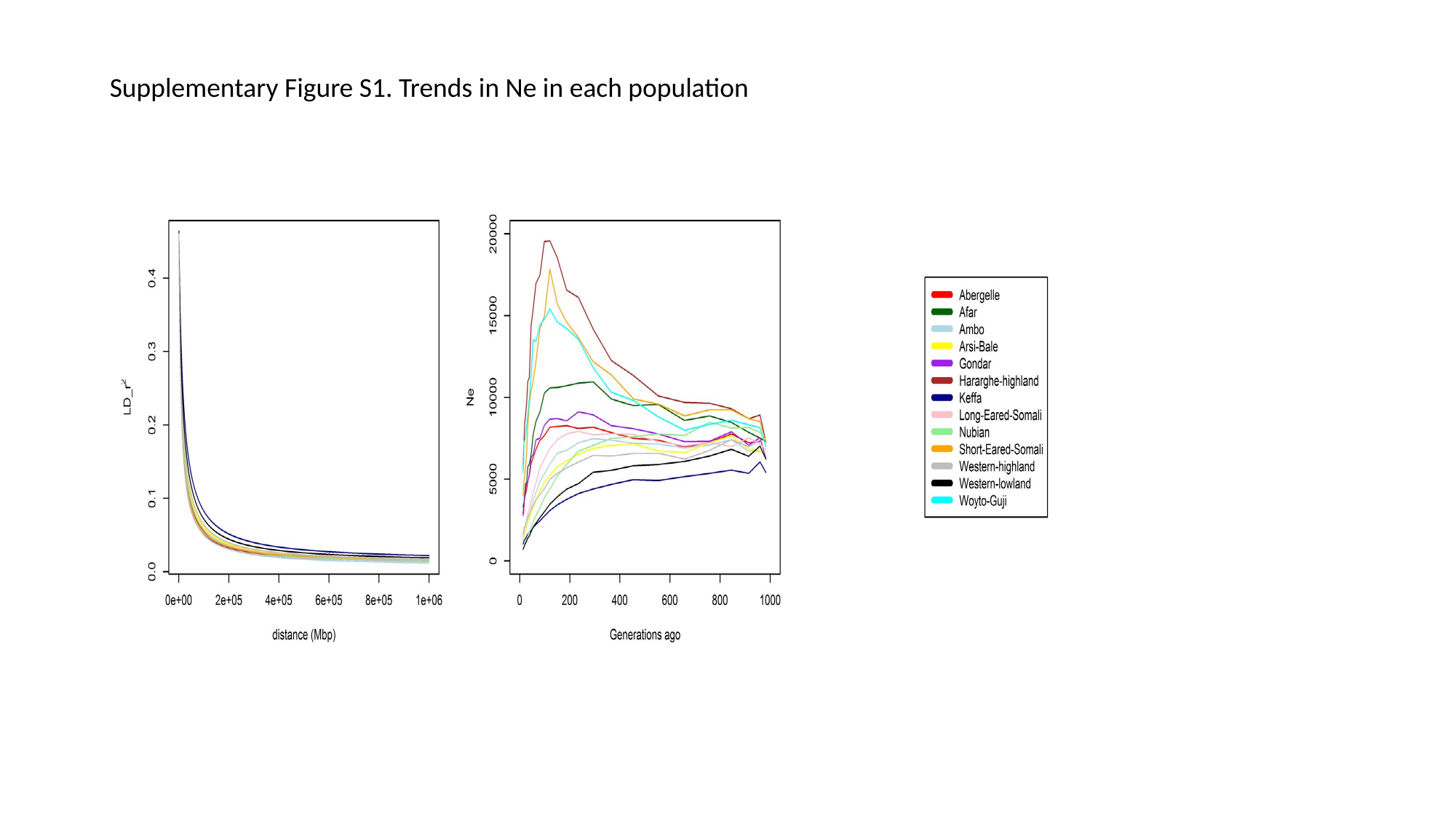

Supplementary Figure S1. Trends in Ne in each population

## Slide 2
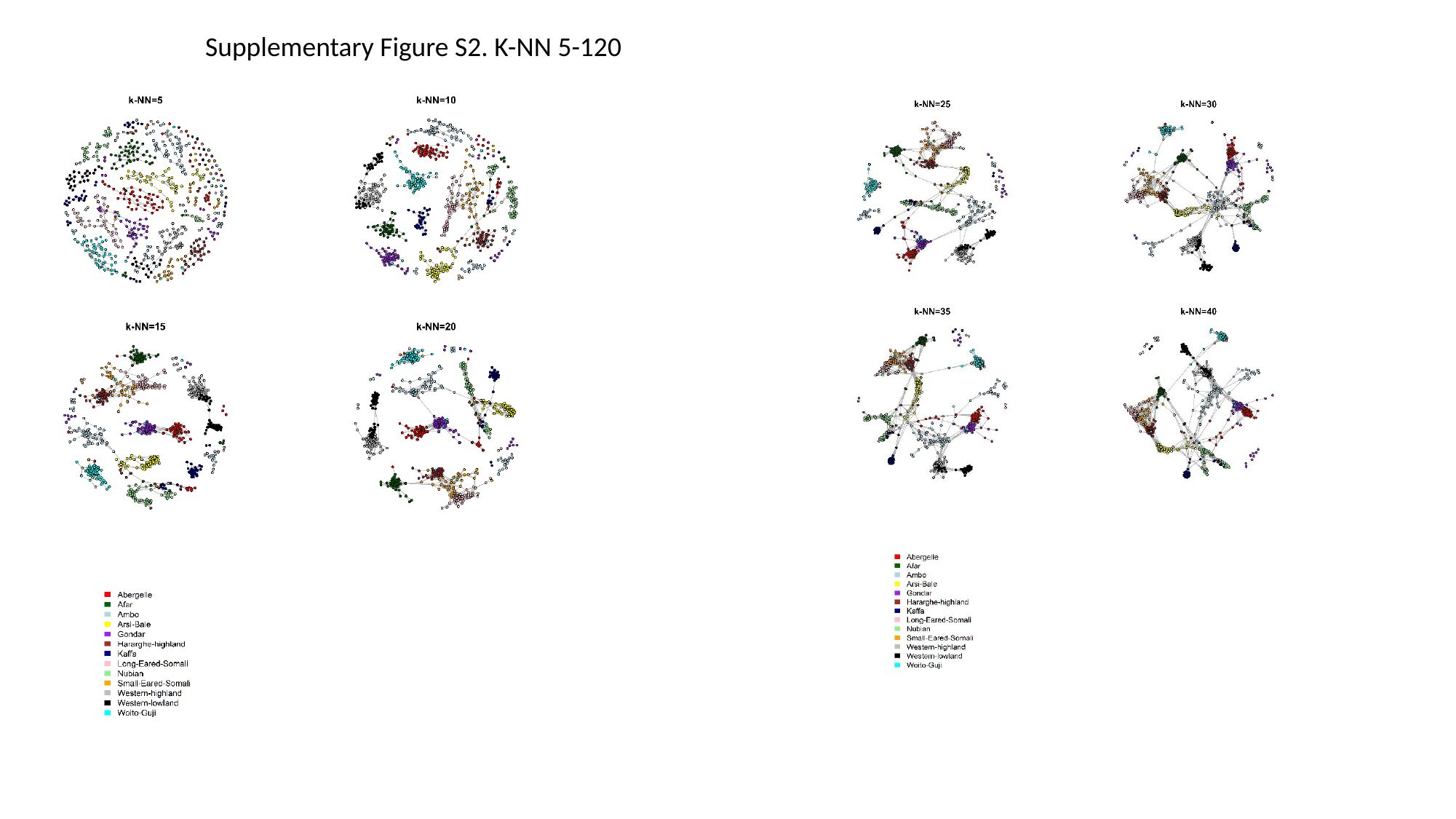

Supplementary Figure S2. K-NN 5-120

## Slide 3
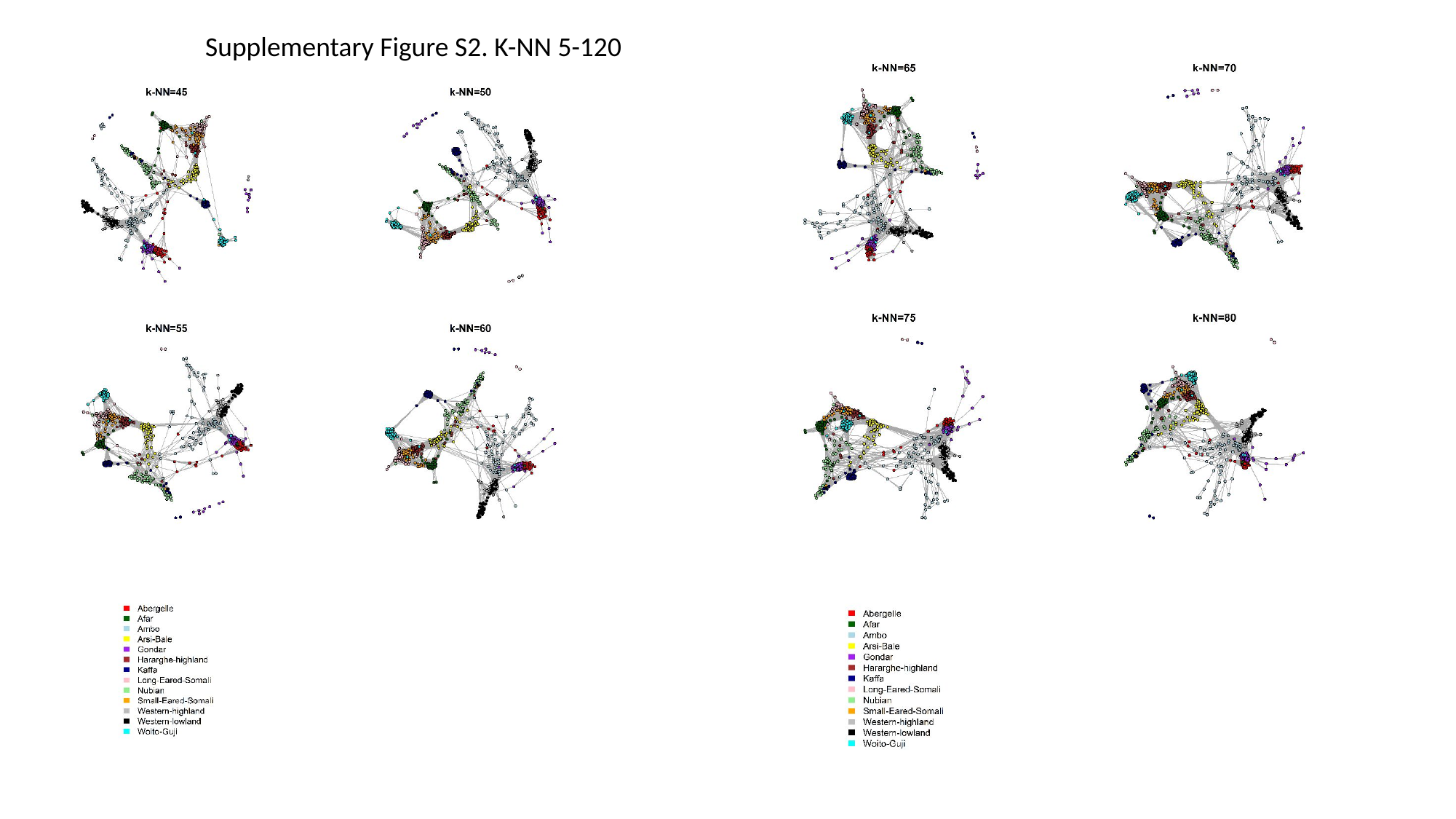

Supplementary Figure S2. K-NN 5-120

## Slide 4
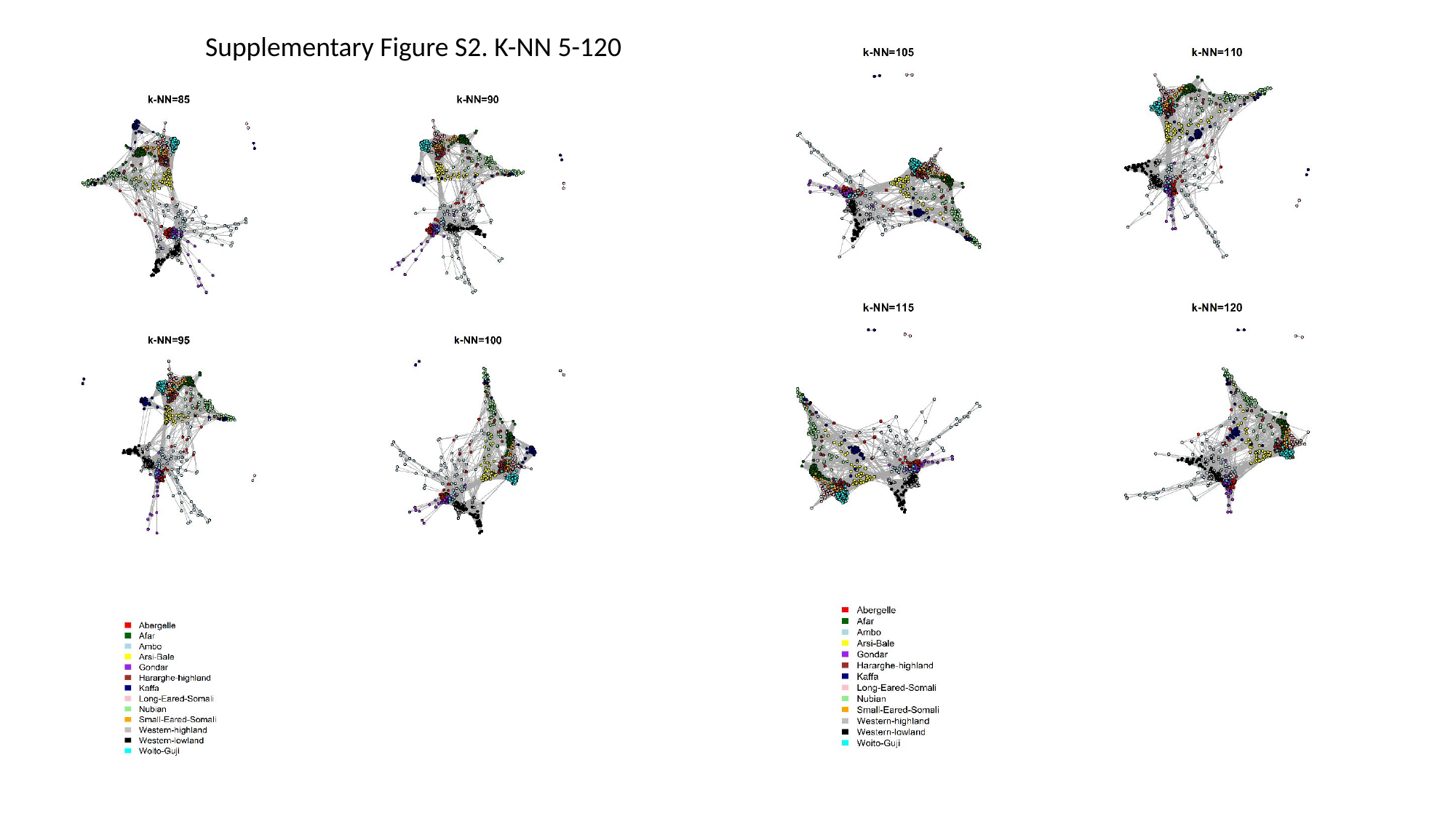

Supplementary Figure S2. K-NN 5-120
