# Supplementary figures and images for: Ethiopian indigenous goats offer insights into past and recent demographic dynamics and local adaptation in sub‐Saharan African goats
Source: Evol Appl. 2021 Jun 15;14(7):1716–31. doi: 10.1111/eva.13118 (PMC8287980; doi:10.1111/eva.13118)

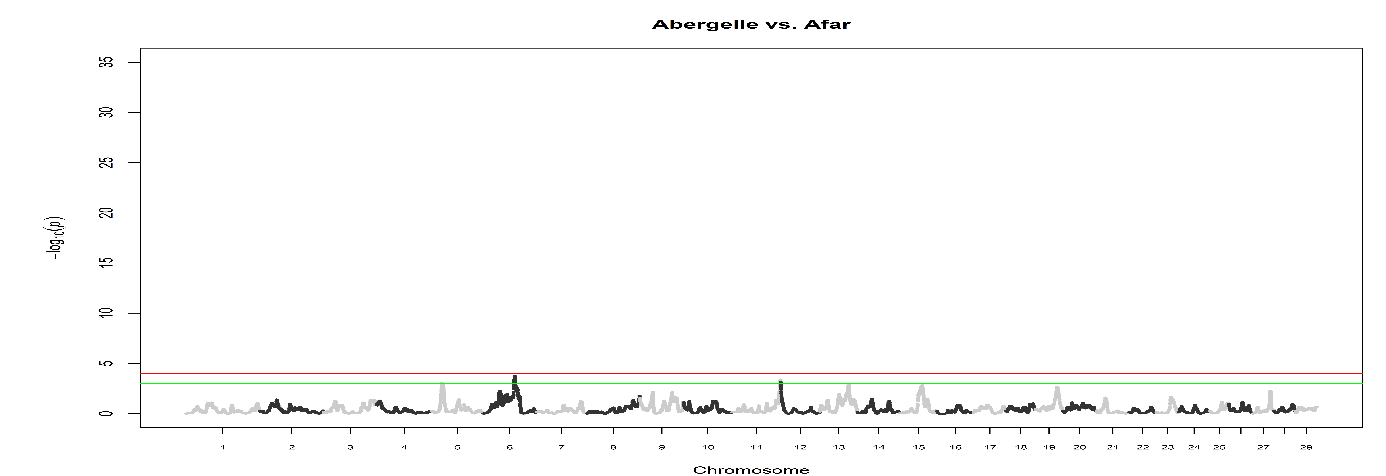


Supplementary Figure S3


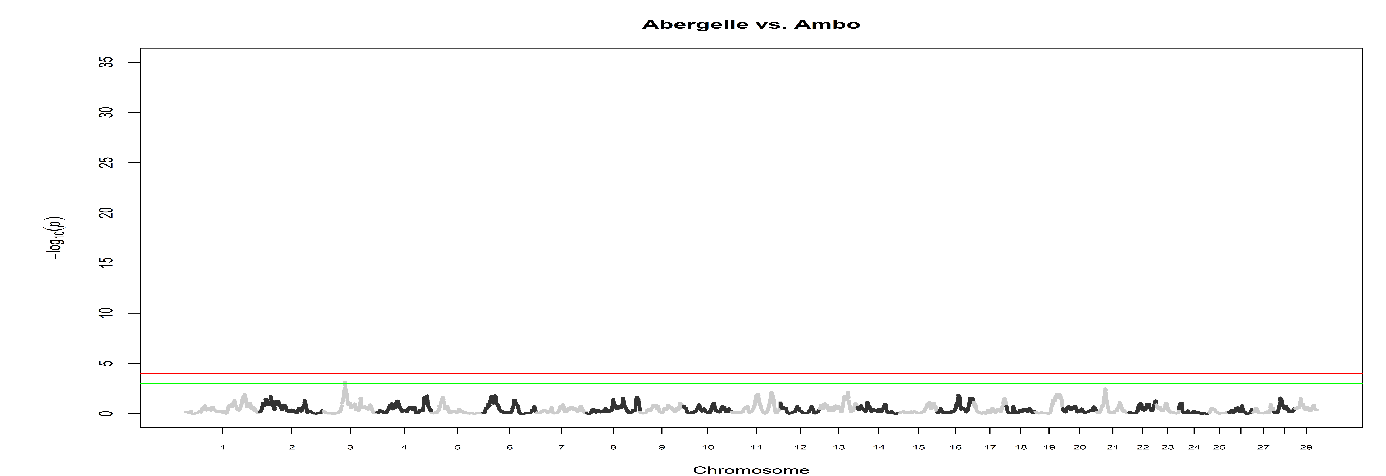


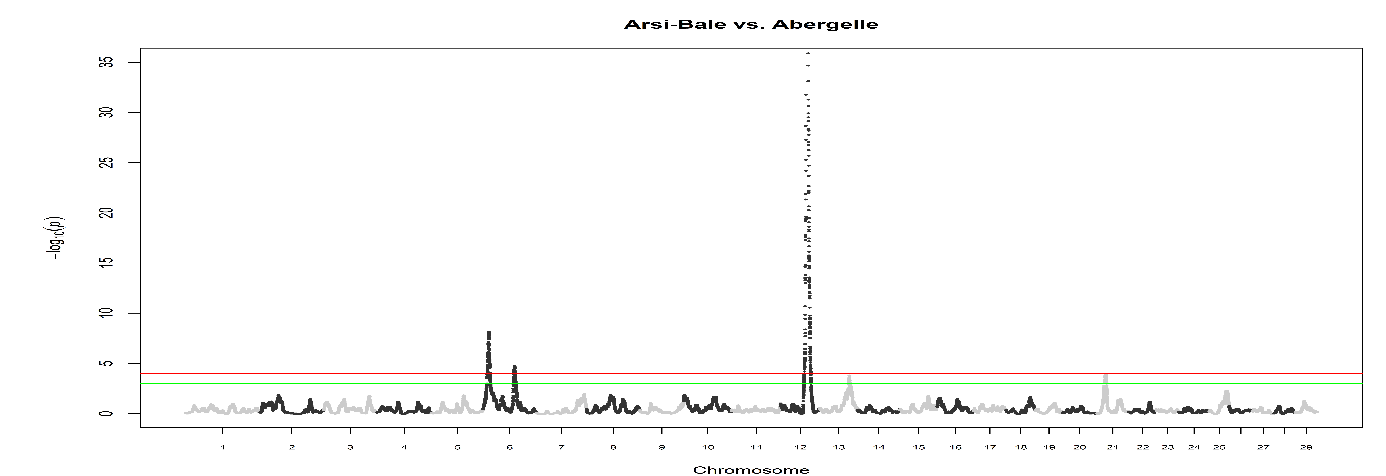


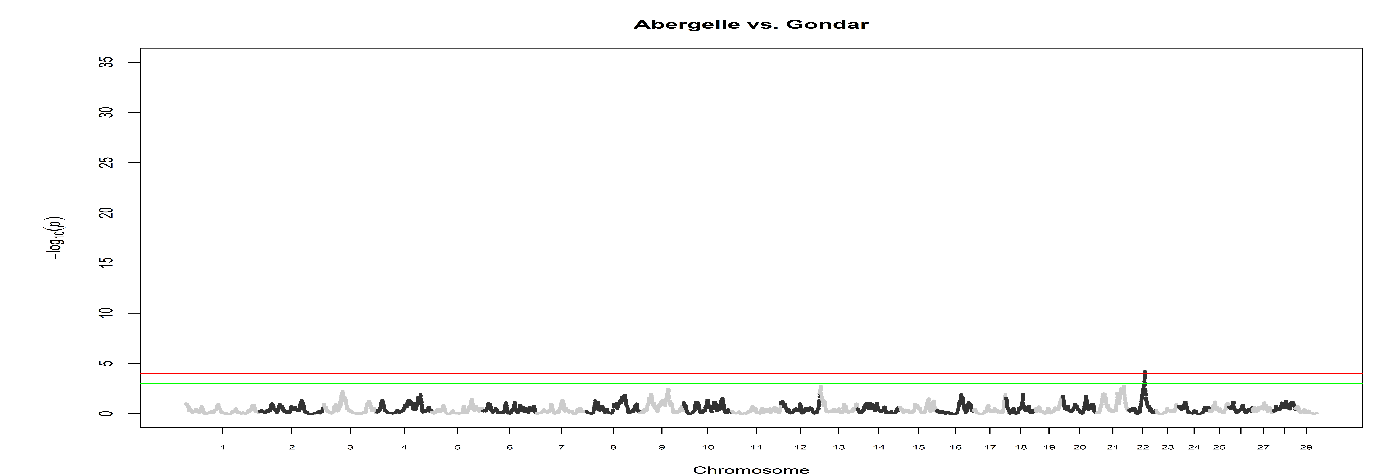


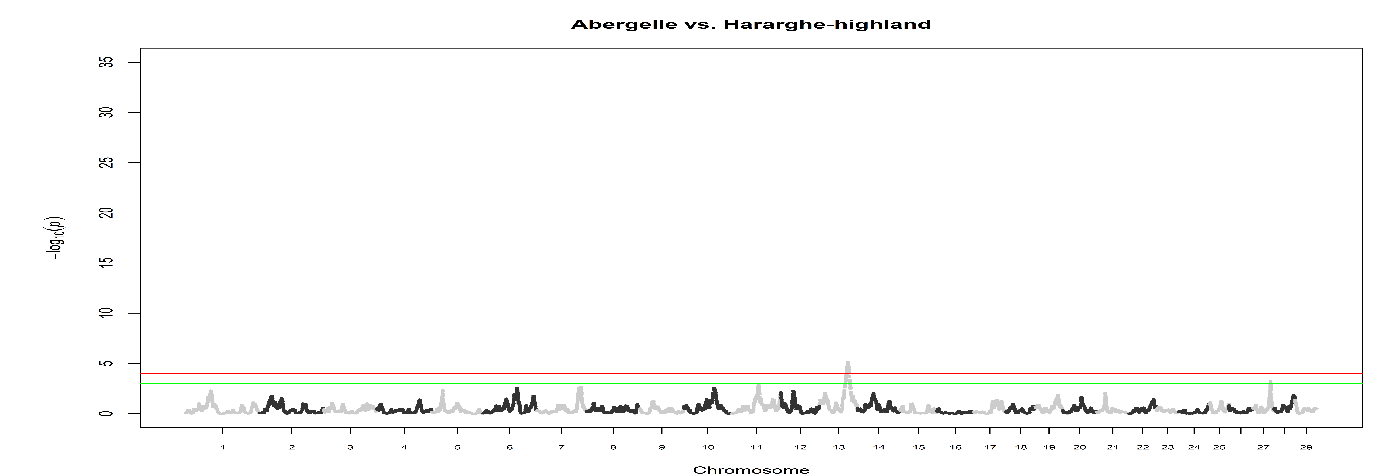


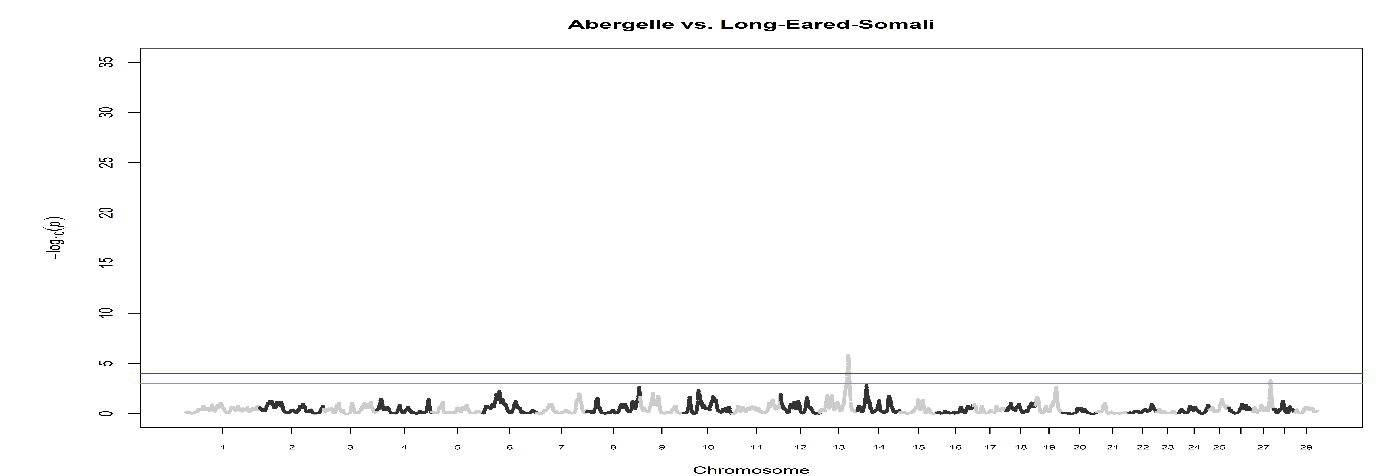


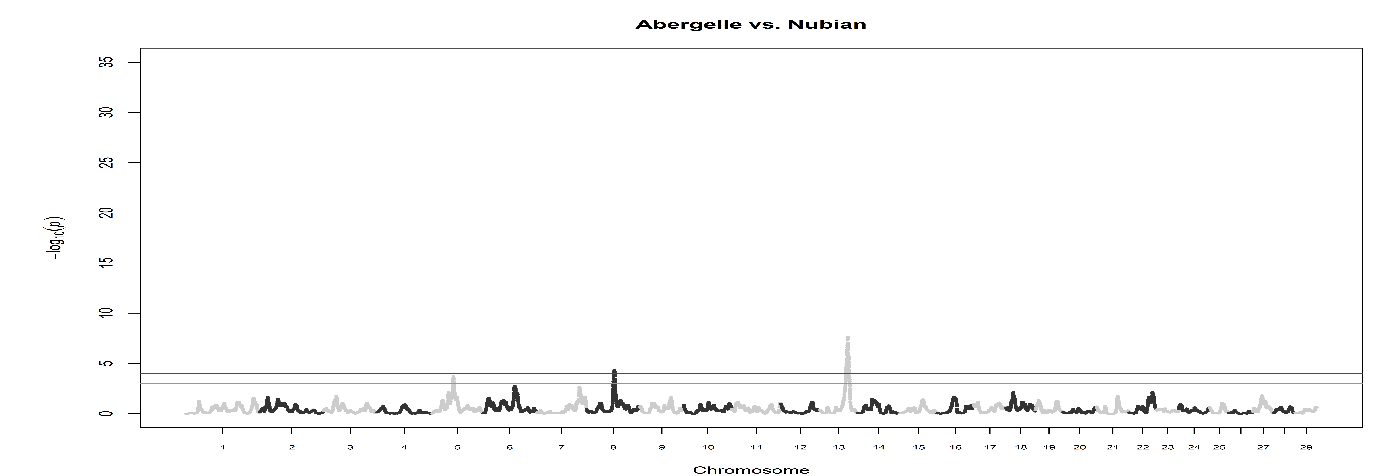


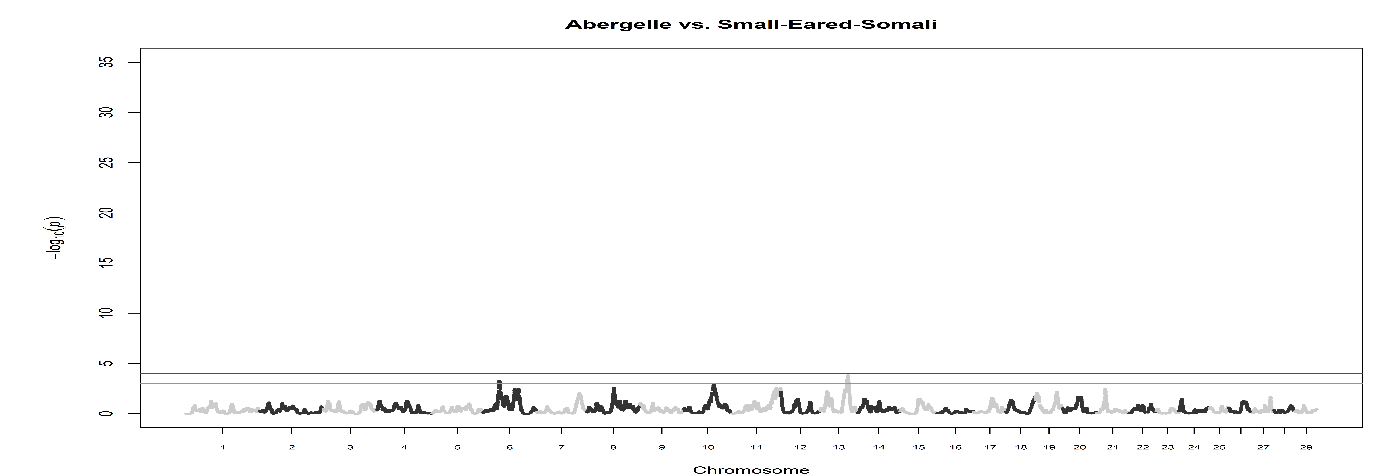


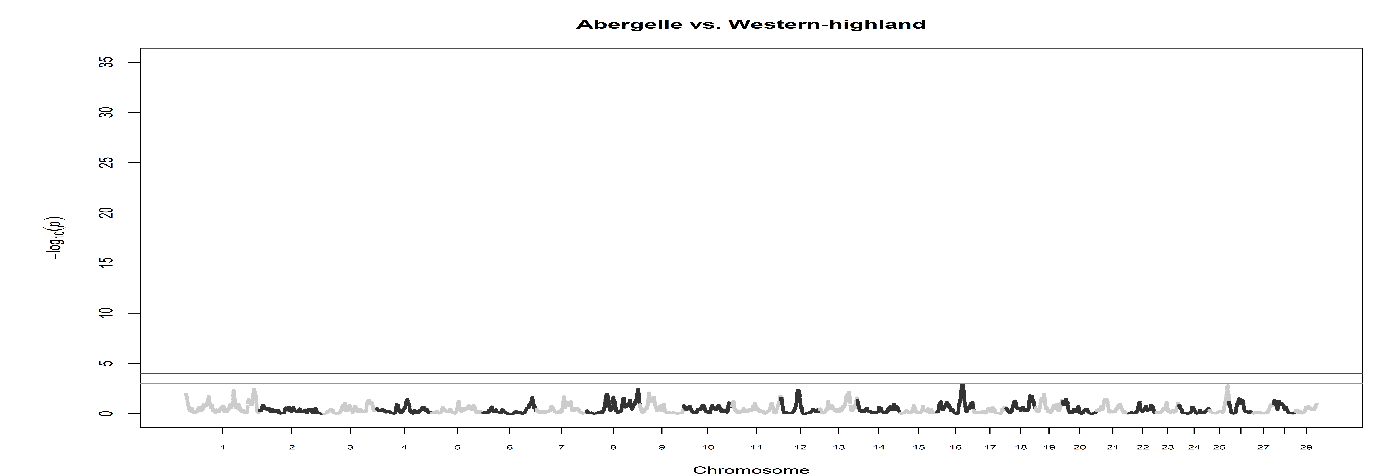


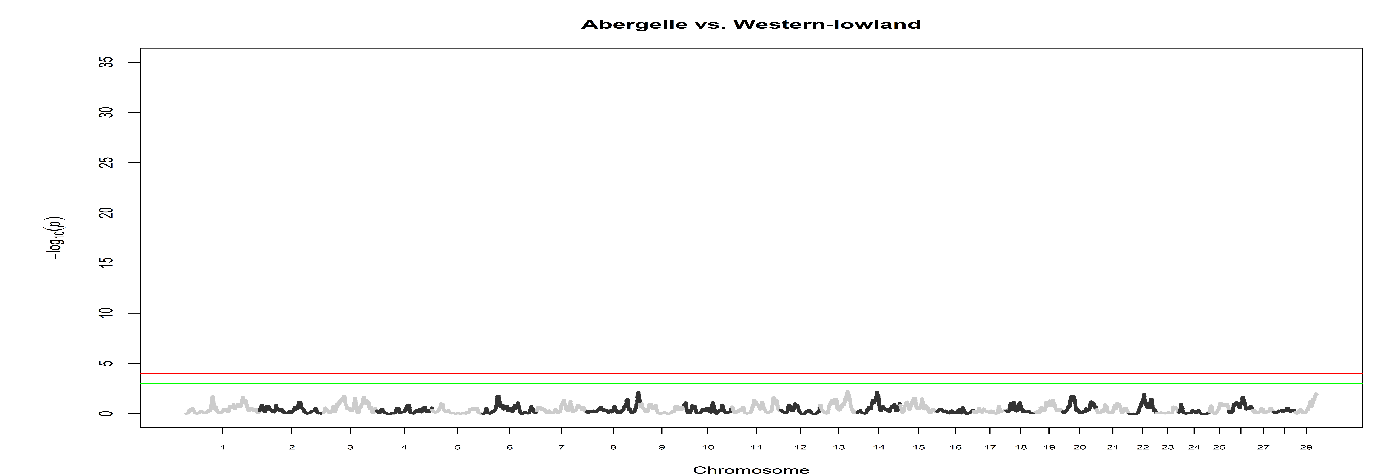


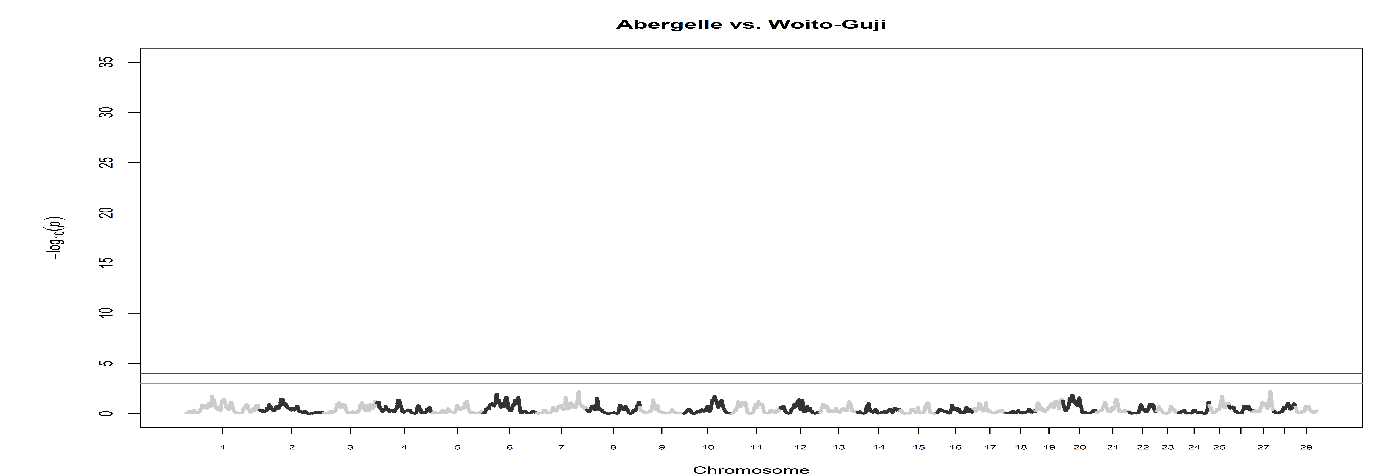


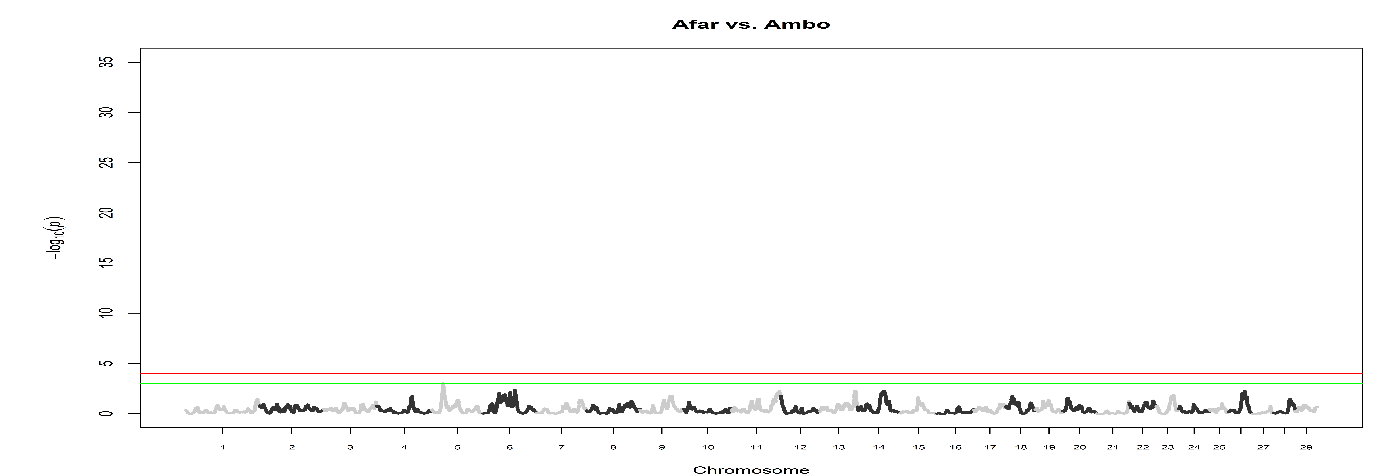


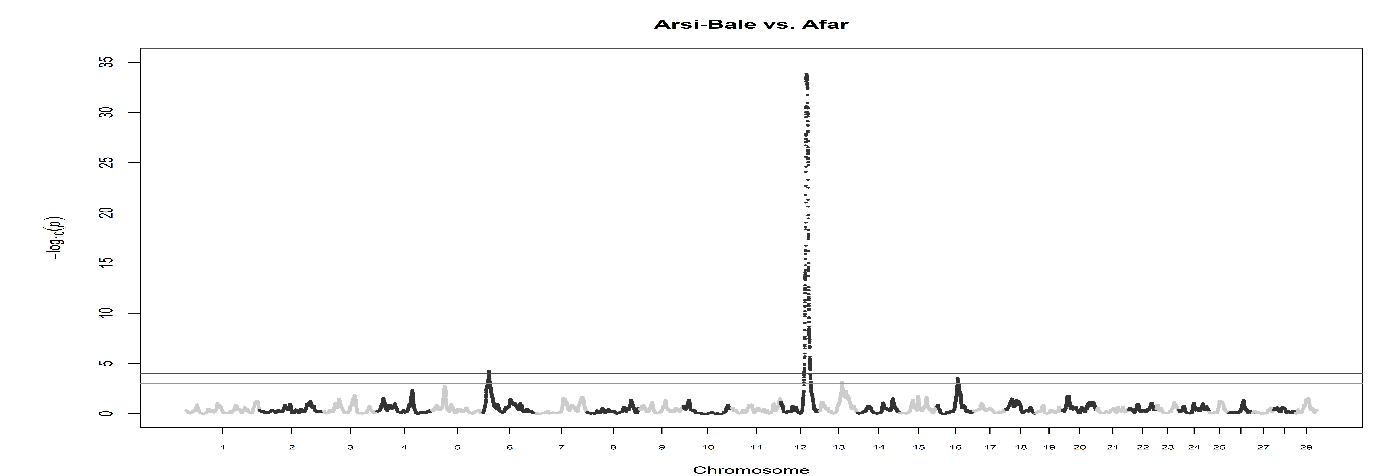


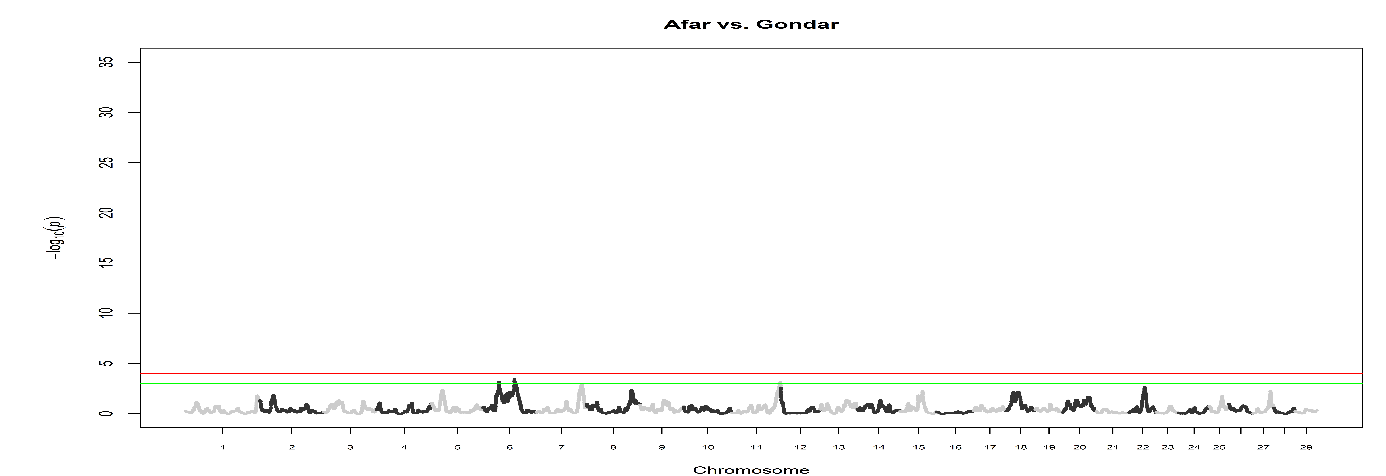


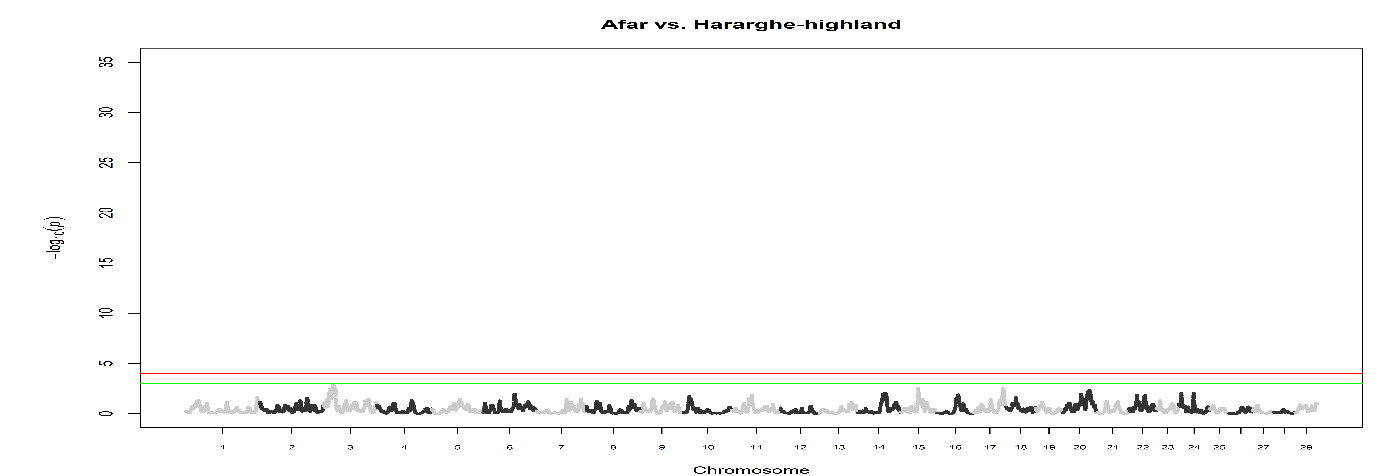


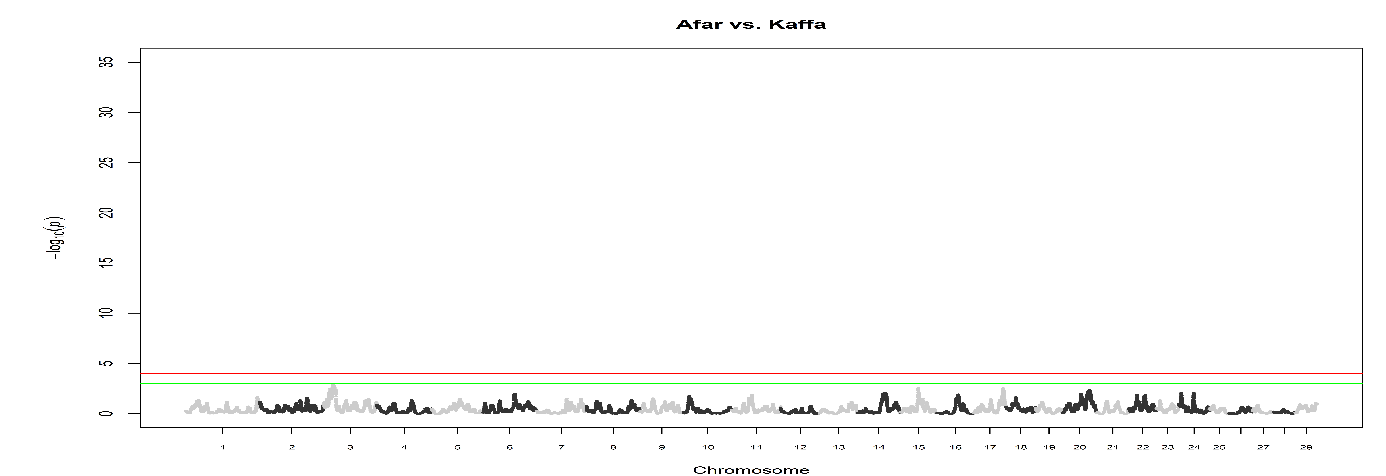


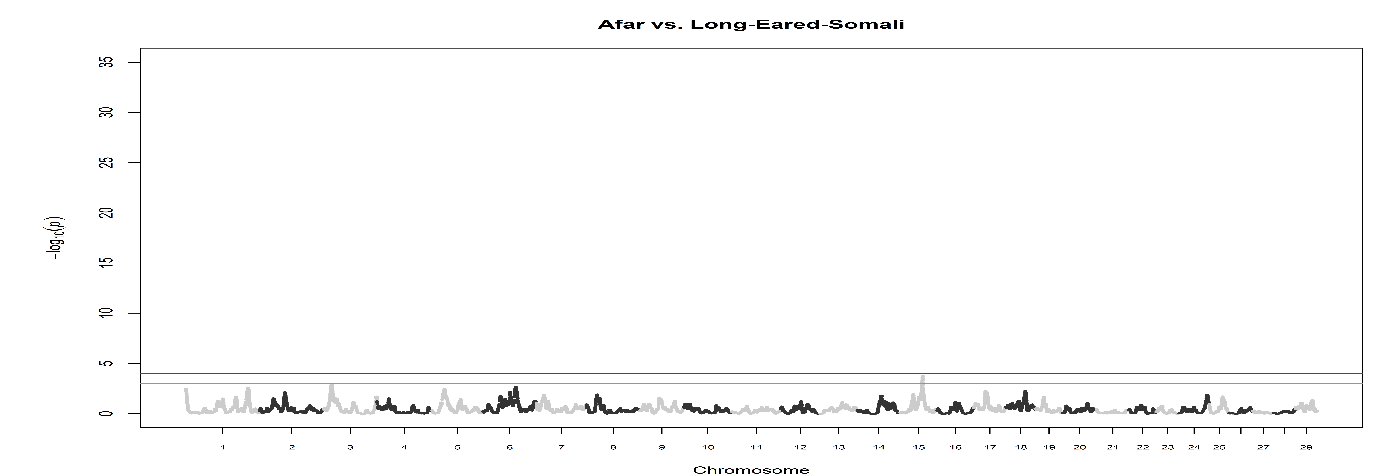


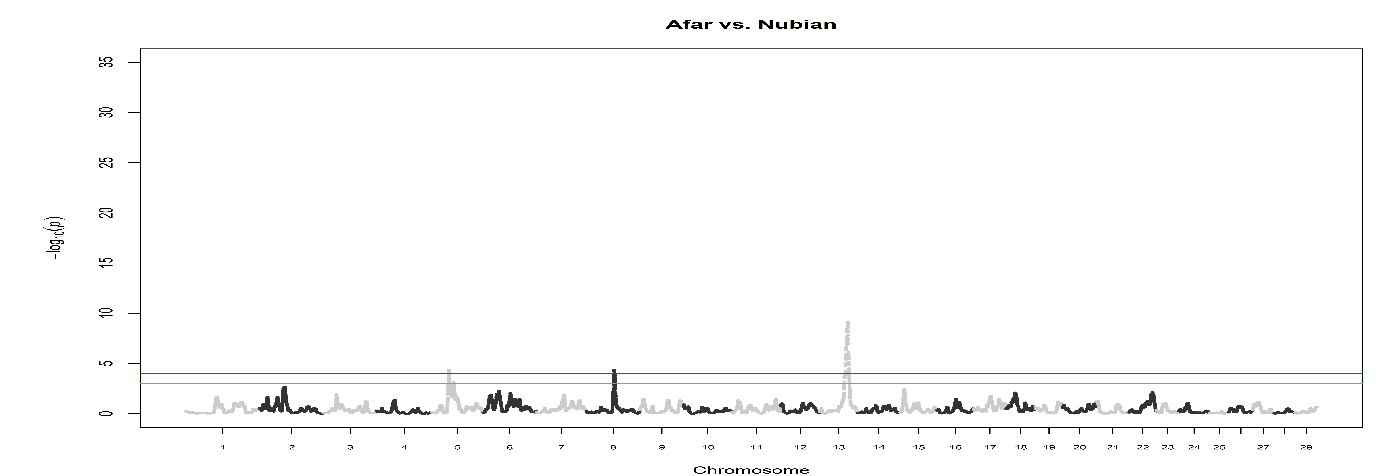


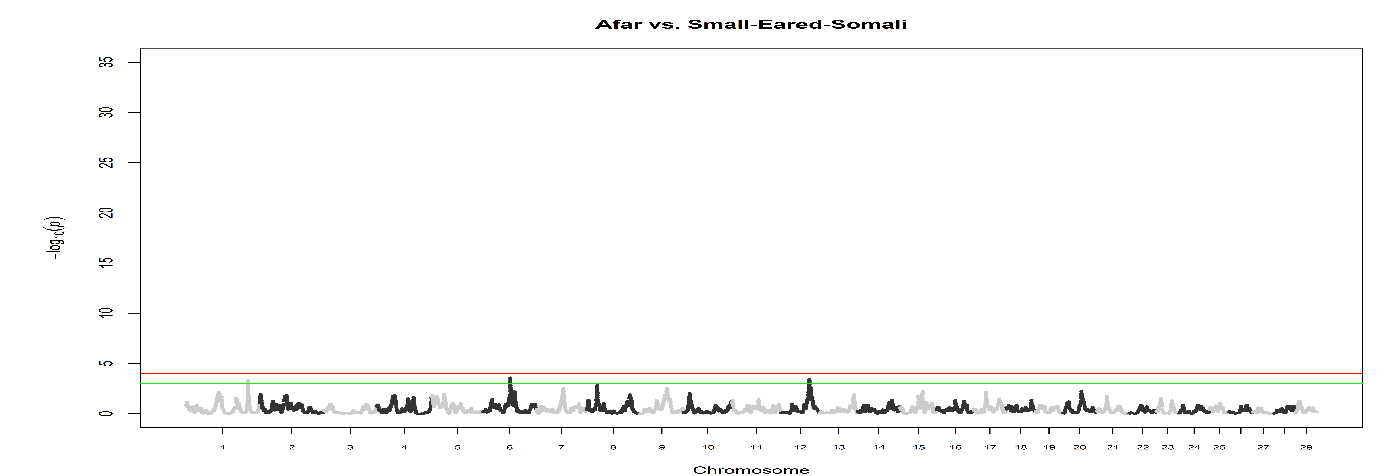


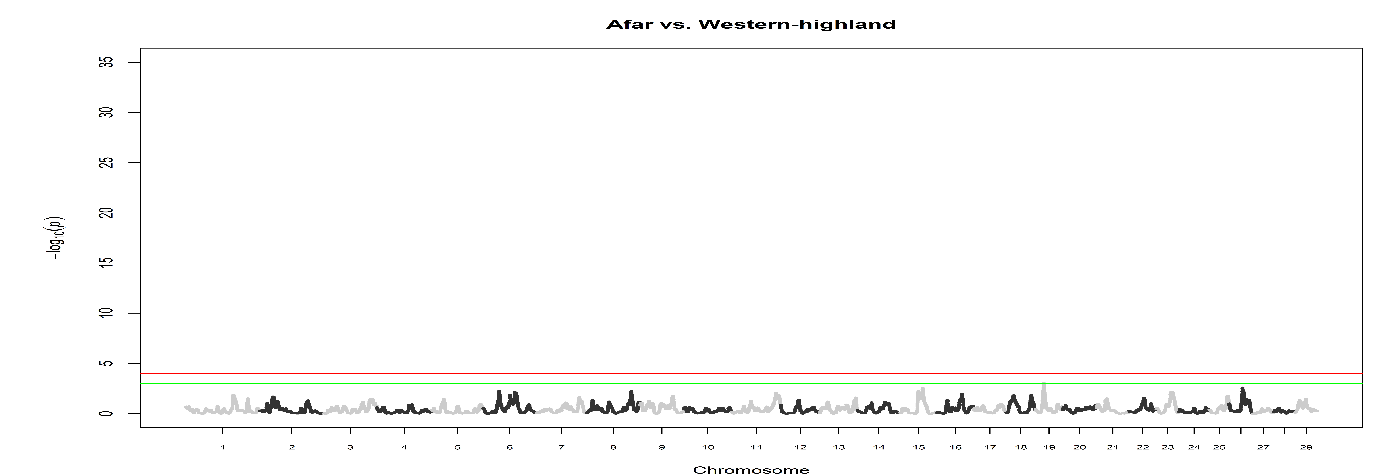


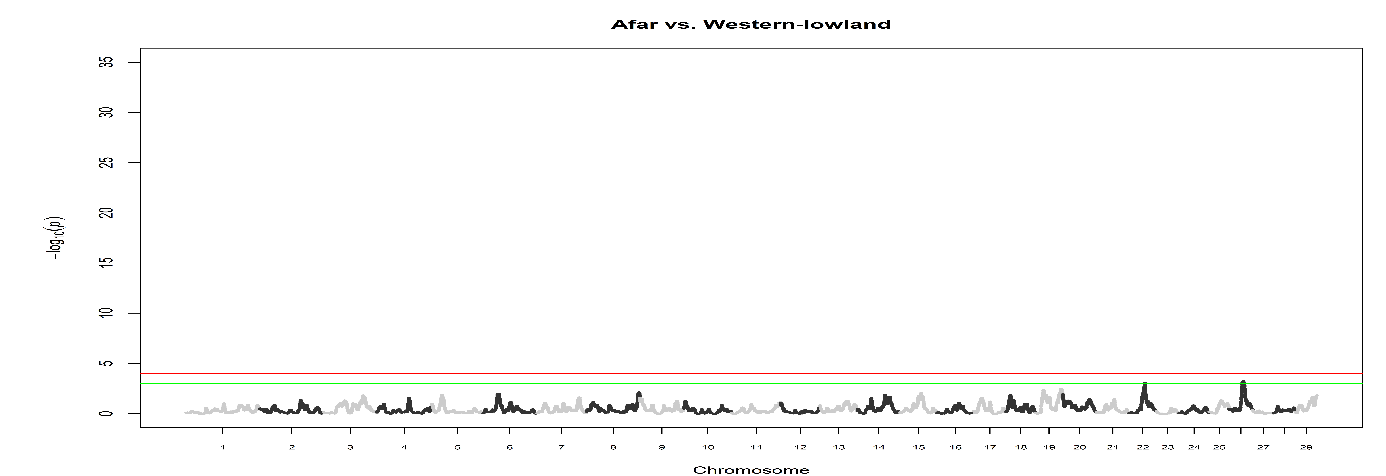


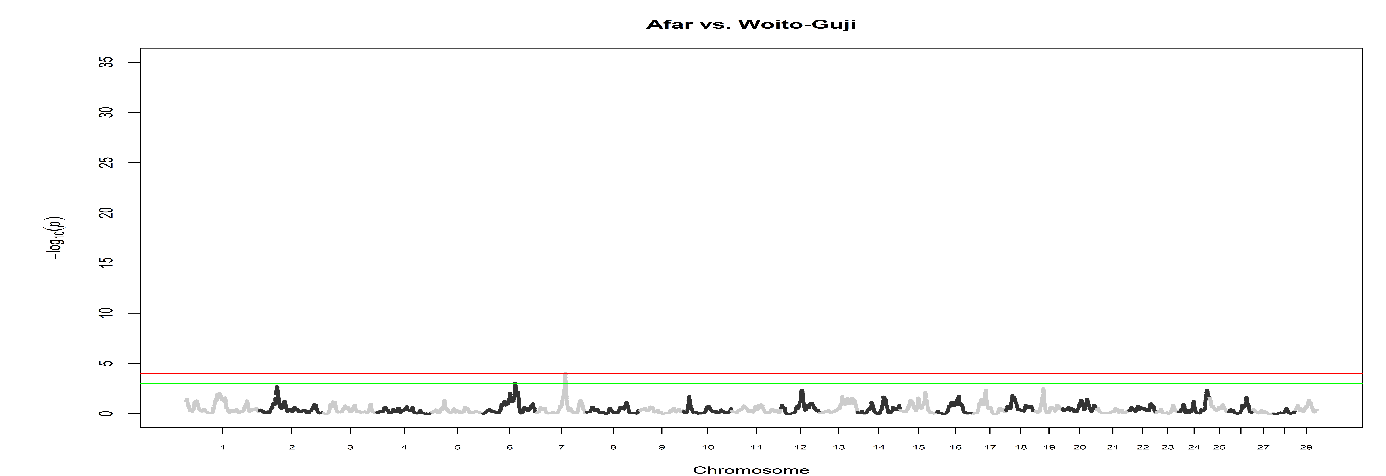


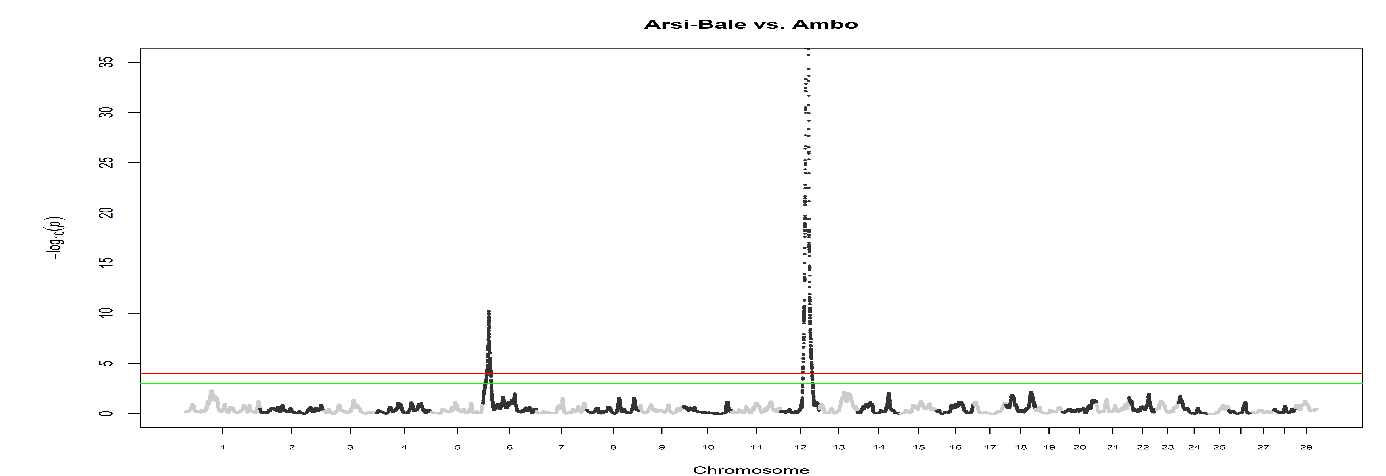


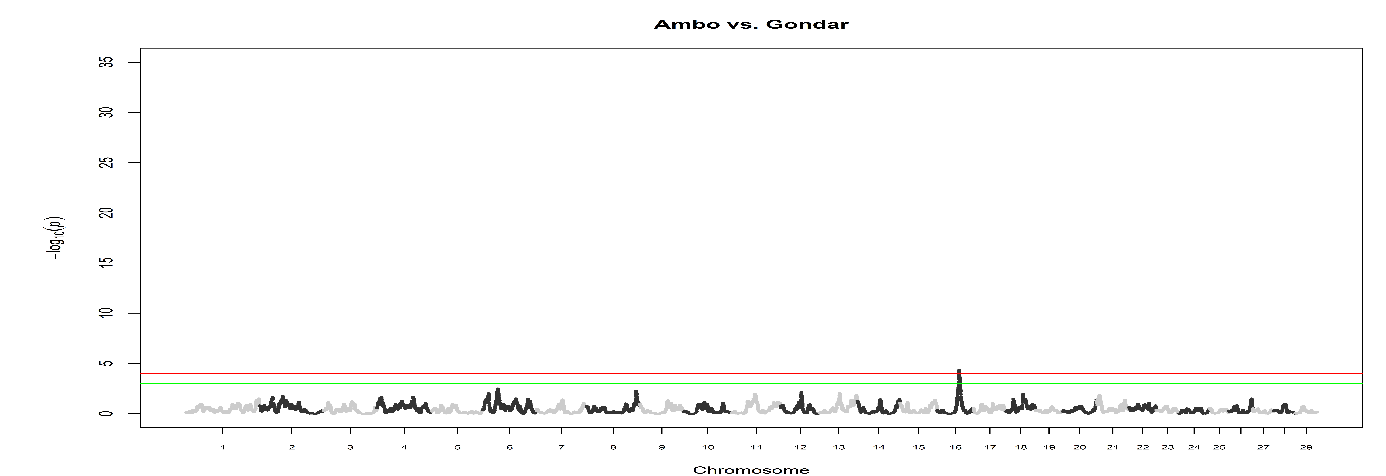


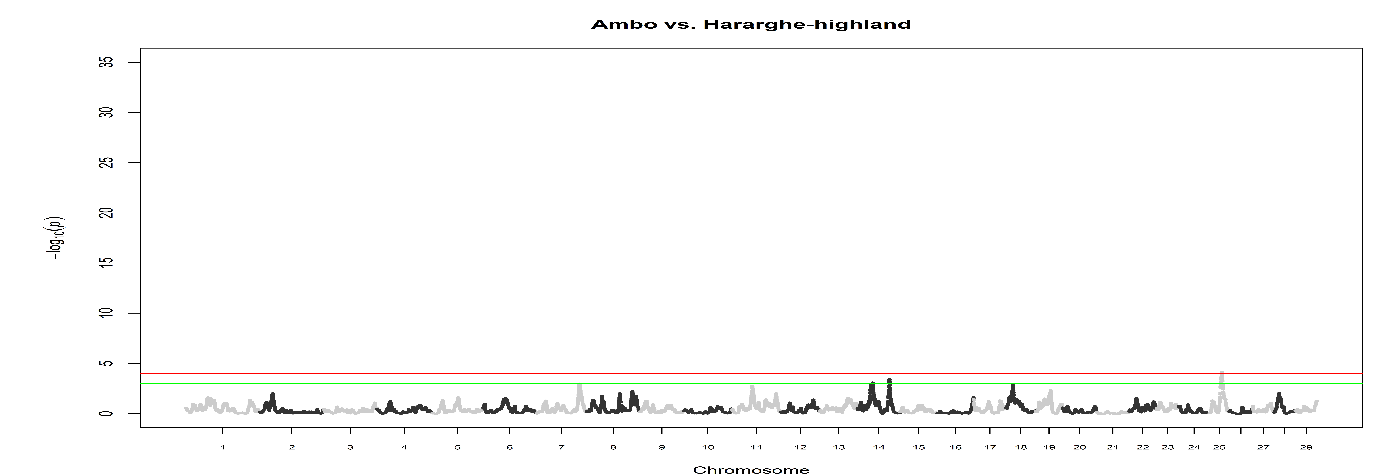


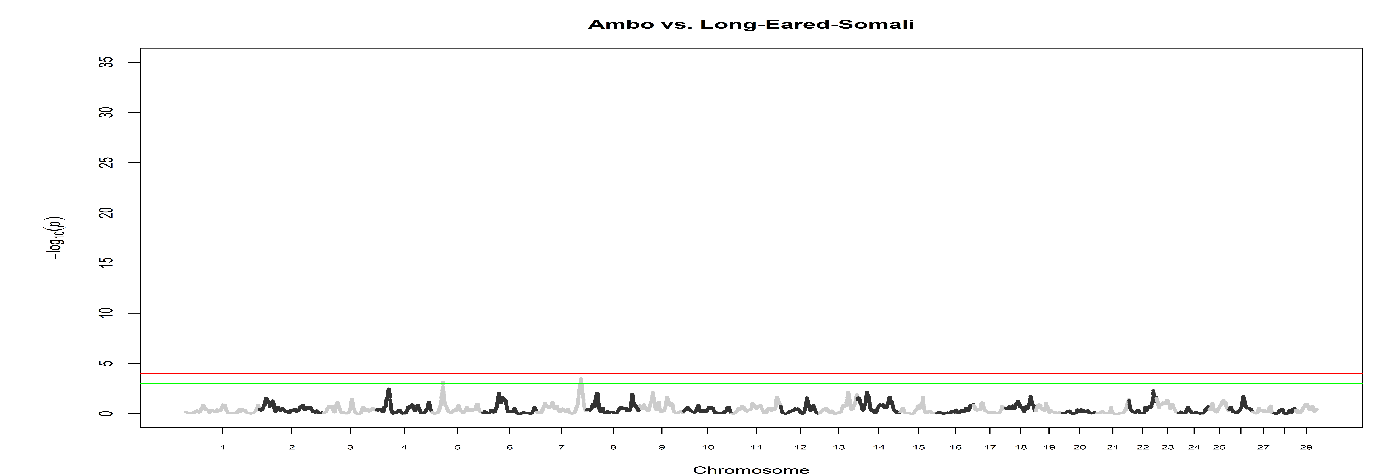


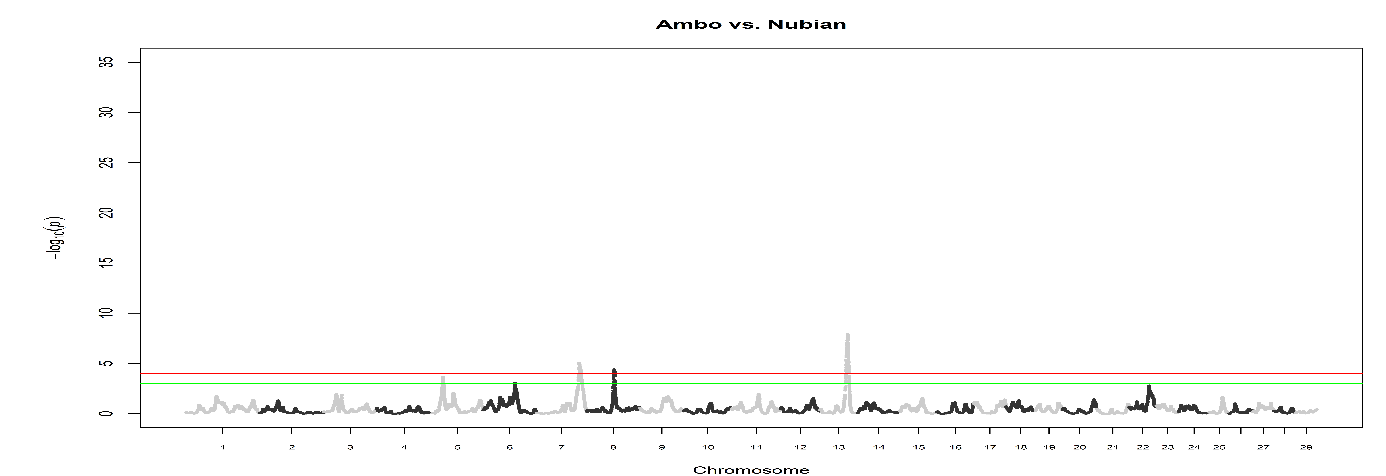


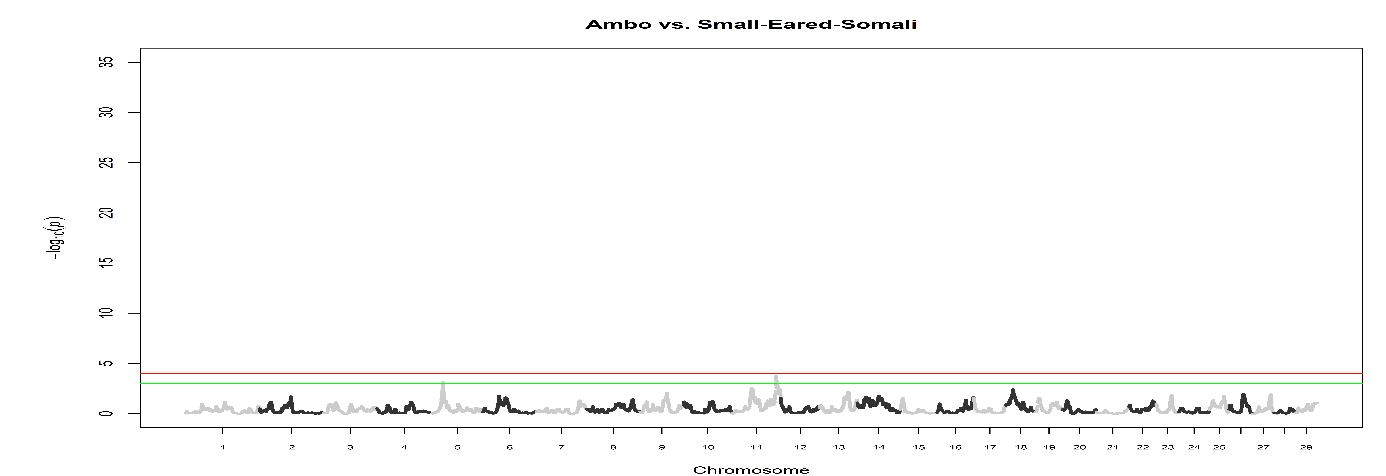


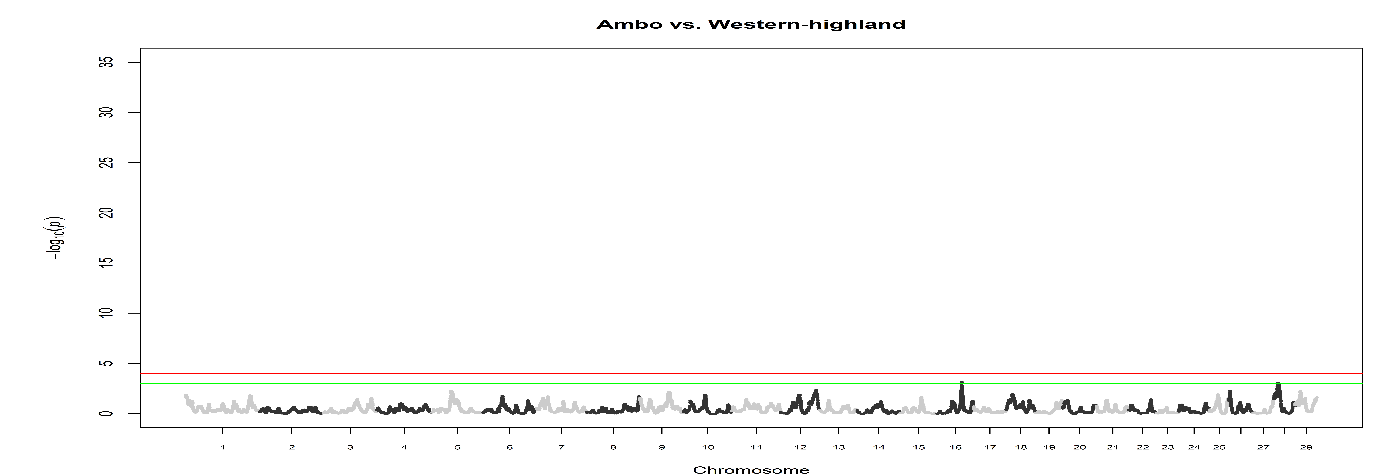


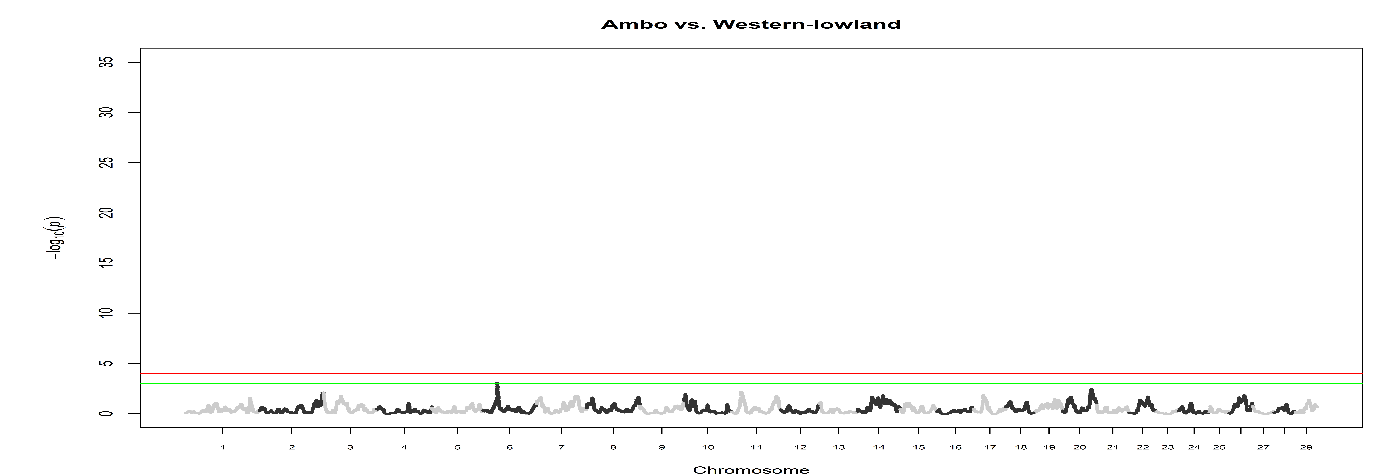


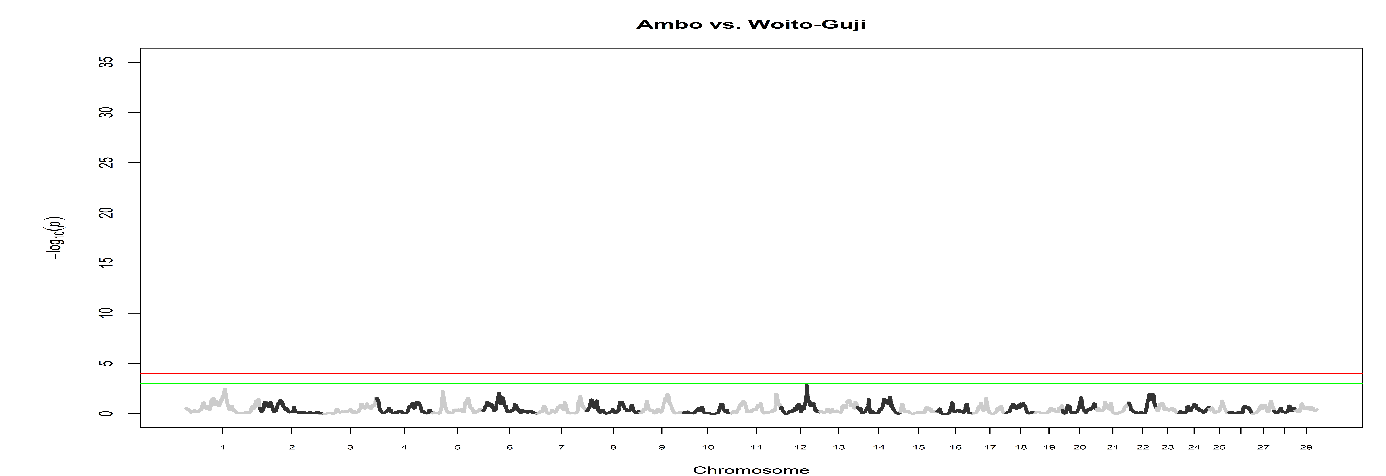


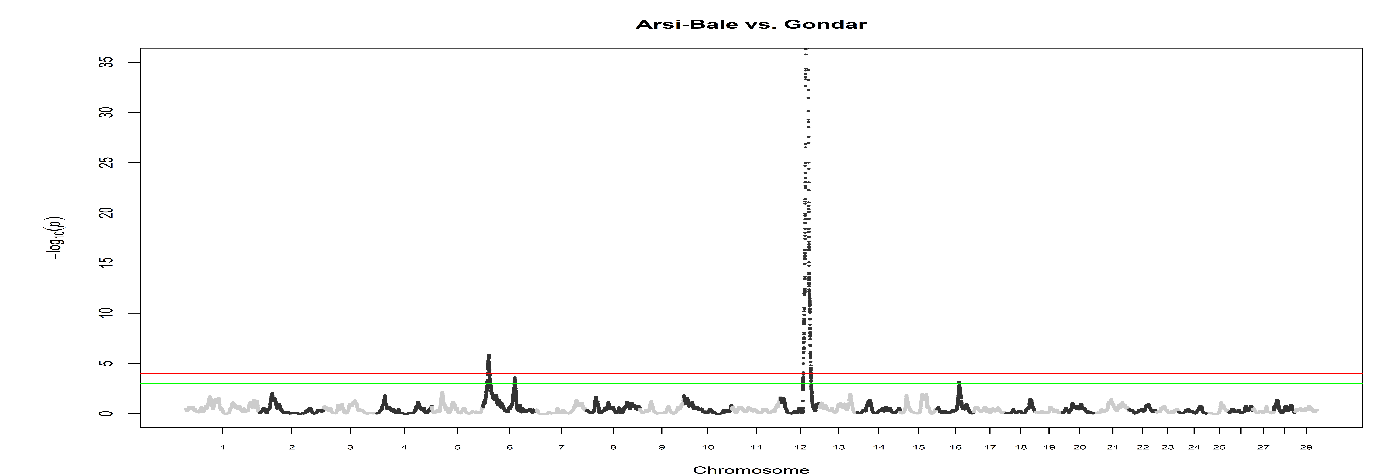


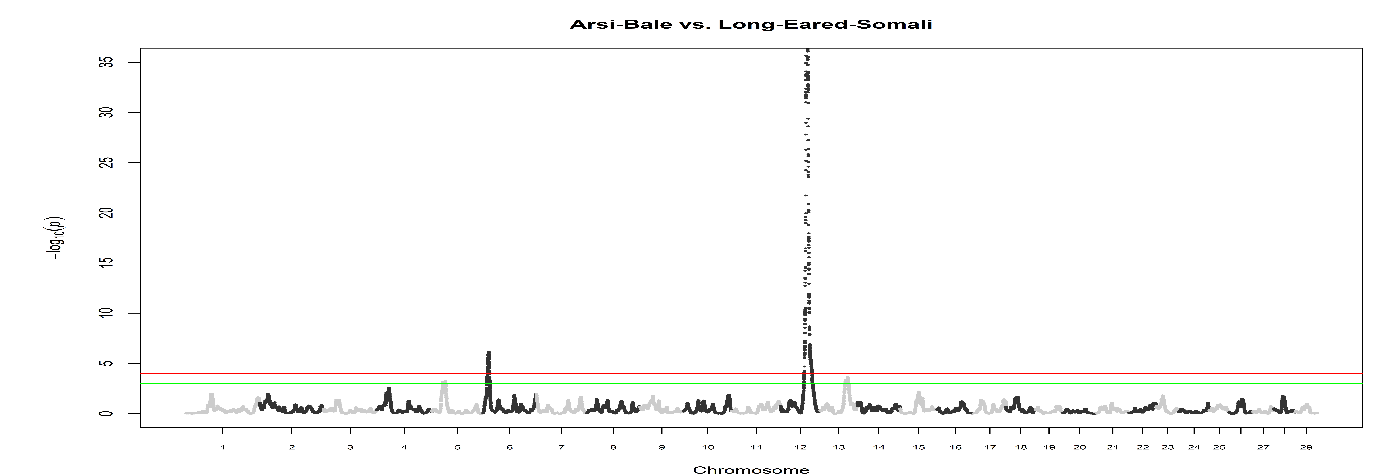


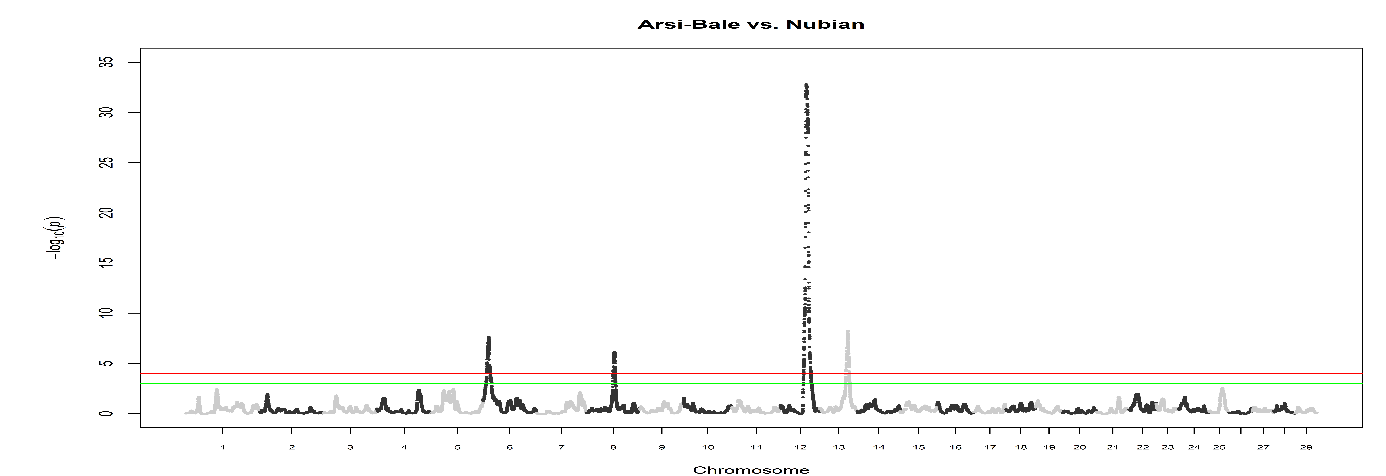


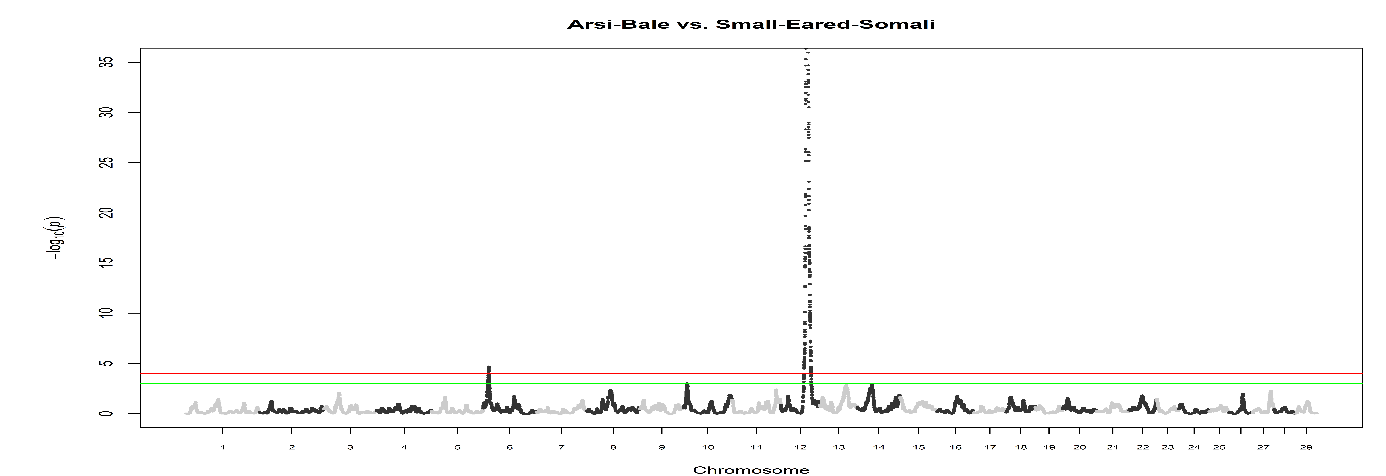


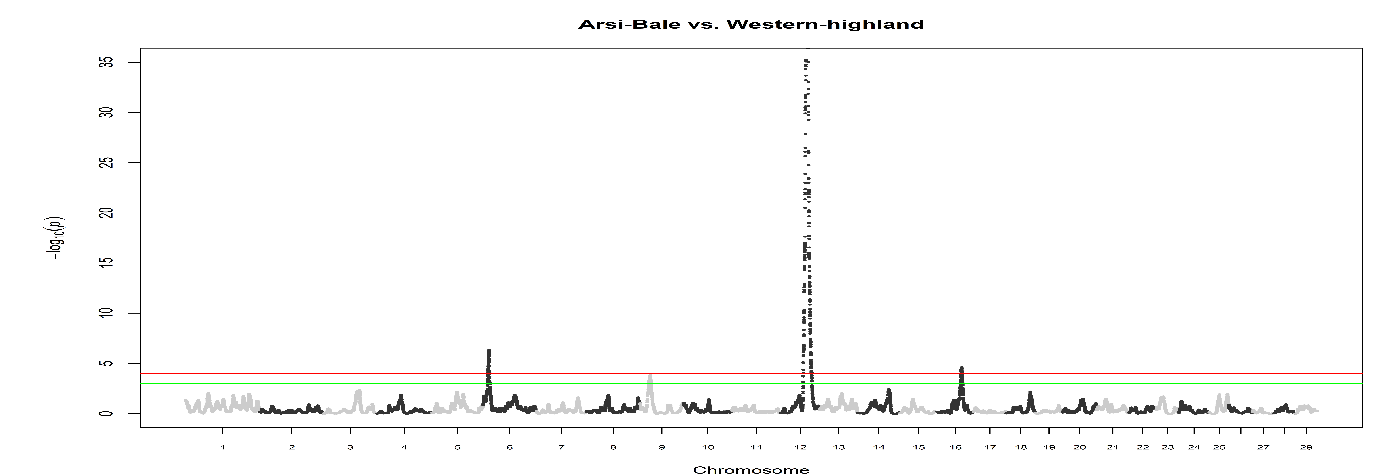


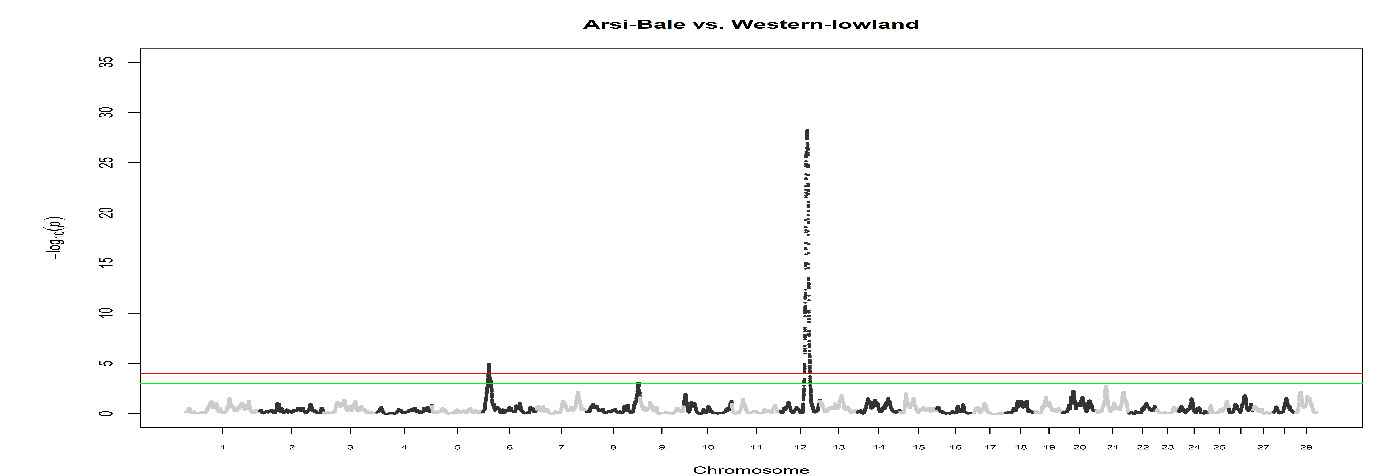


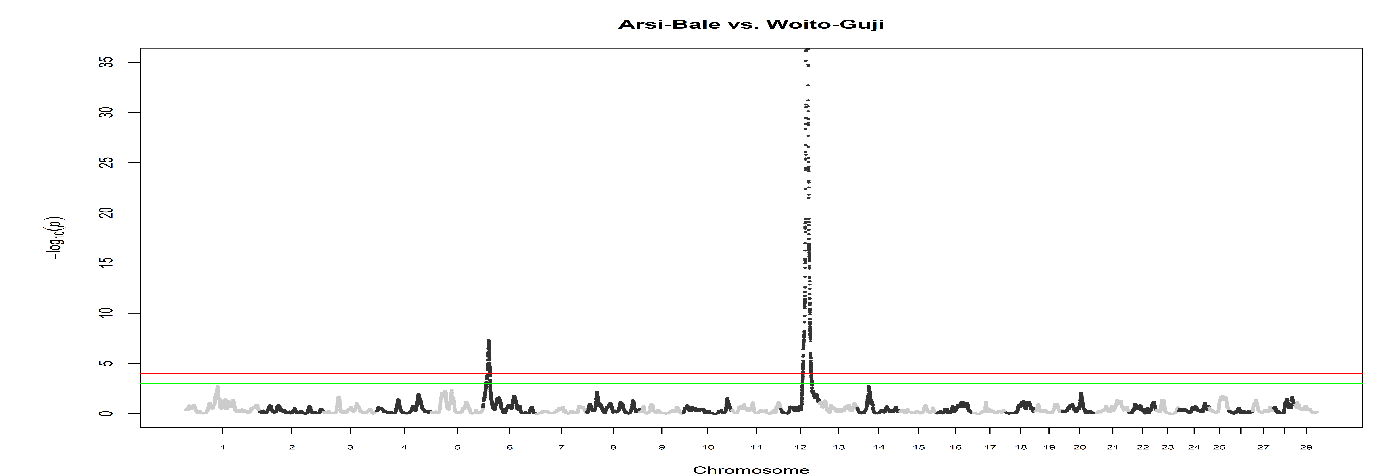


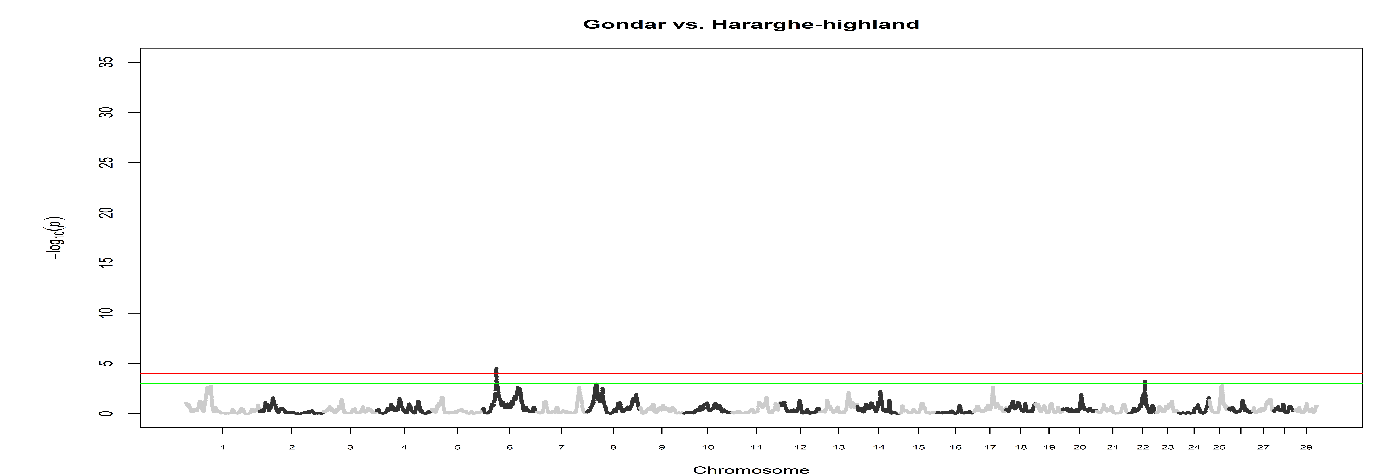


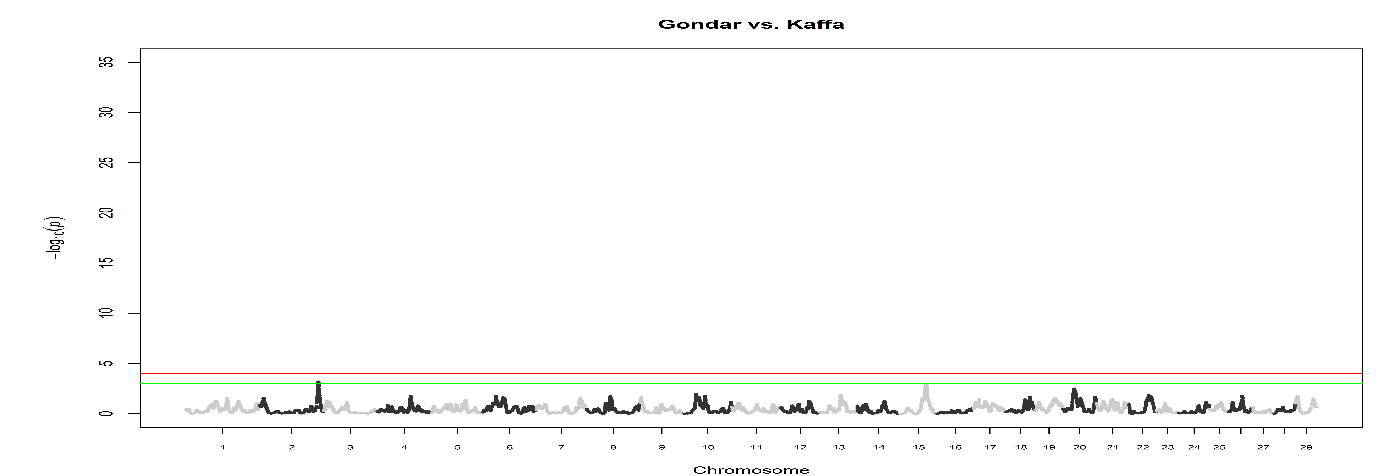


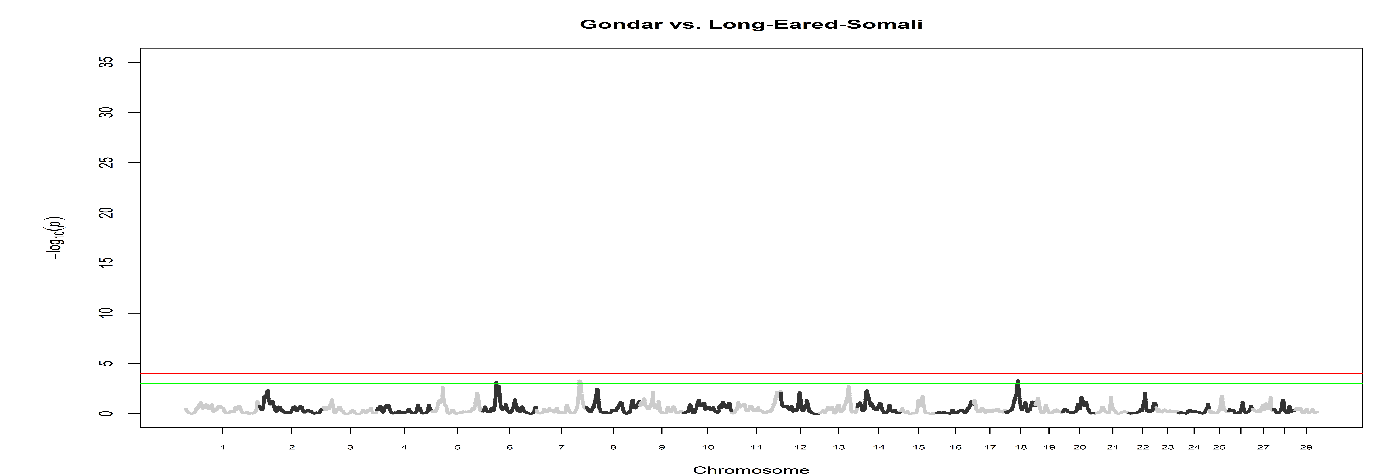


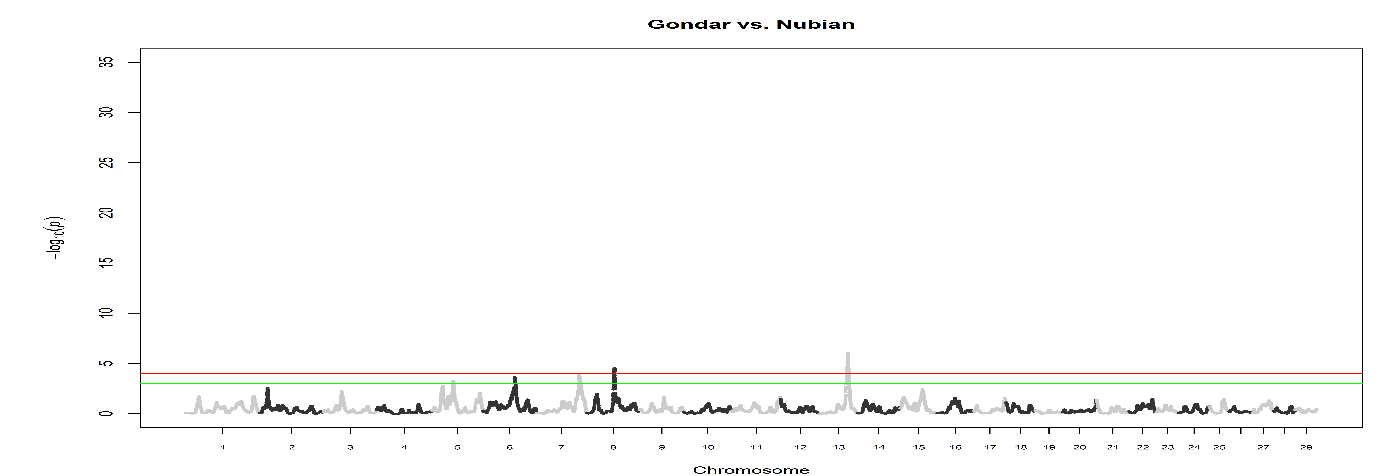


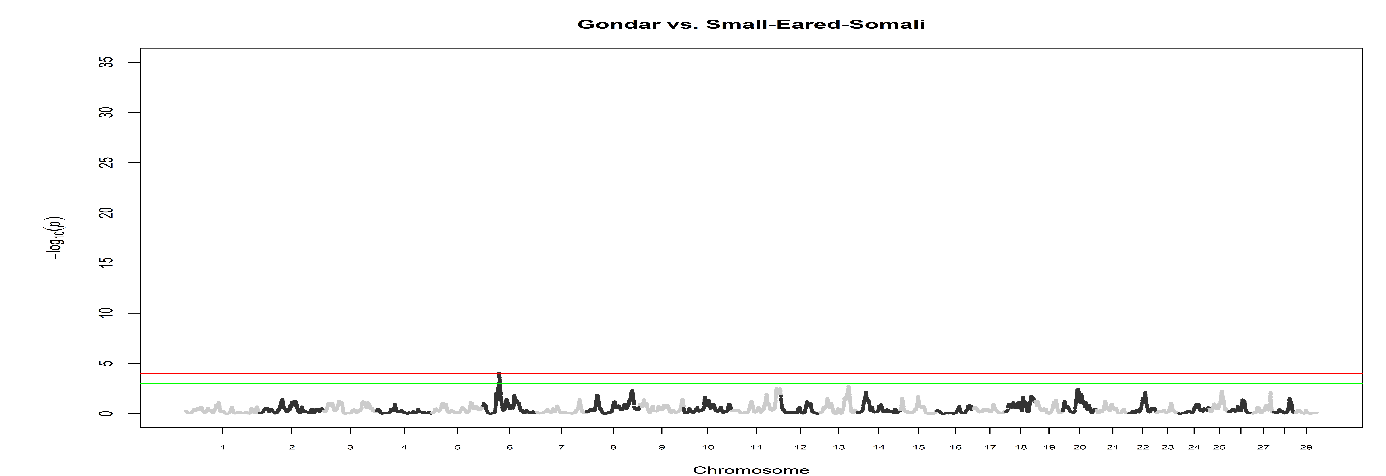


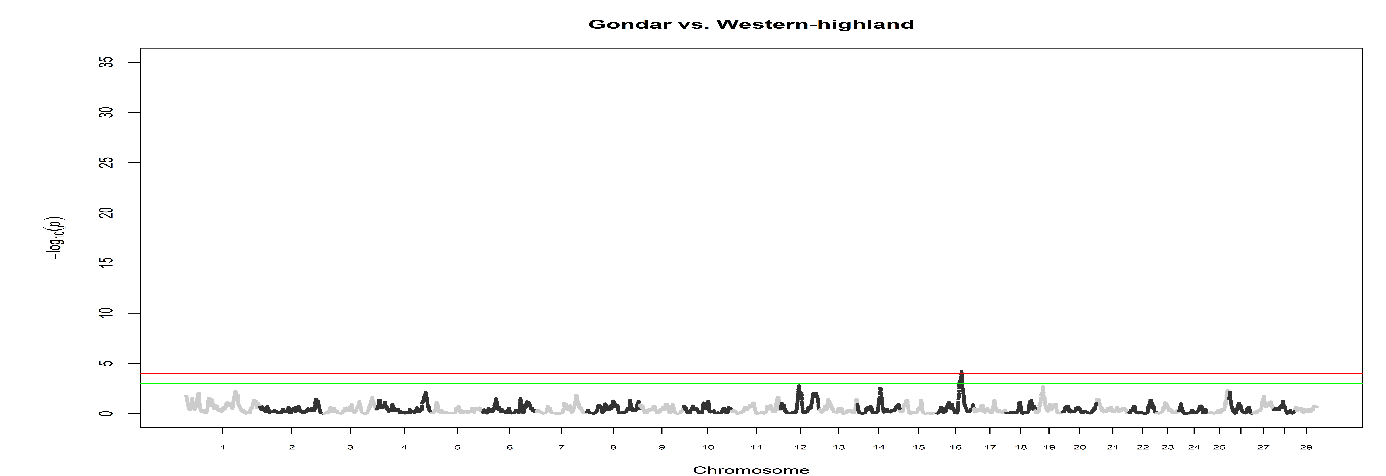


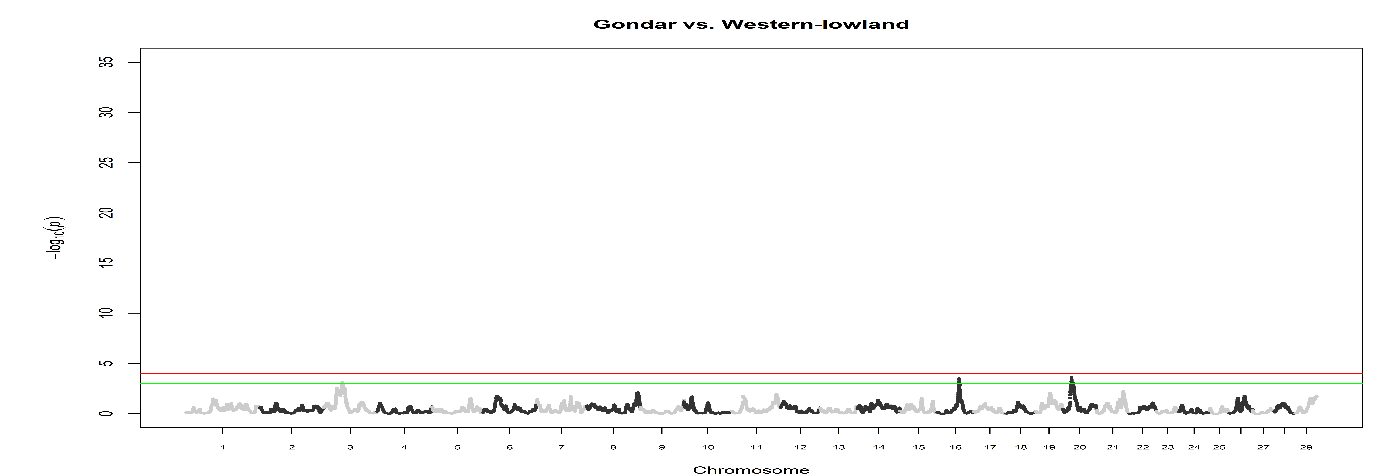


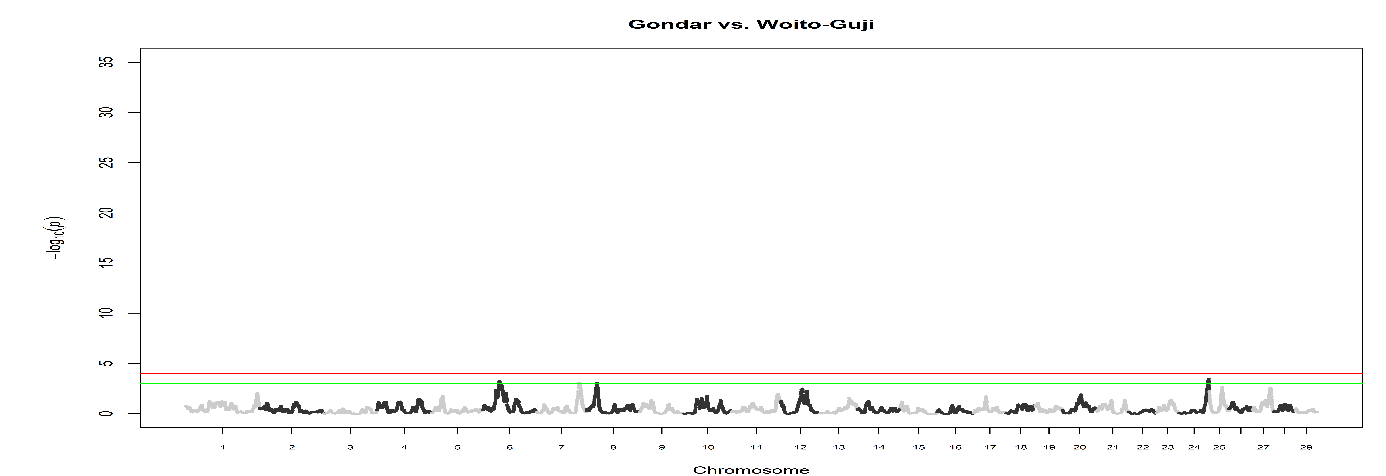


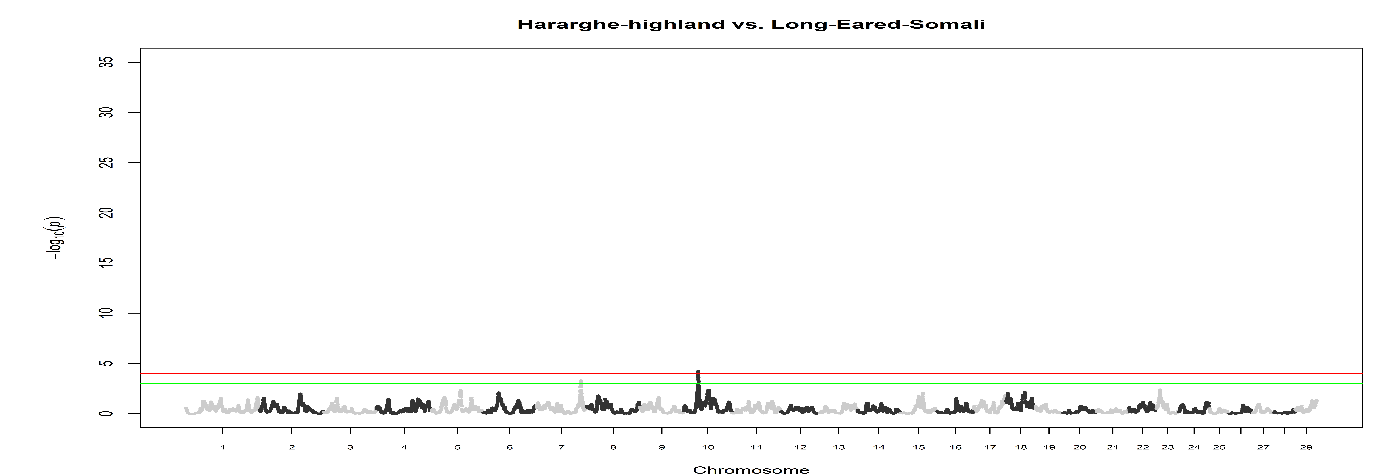


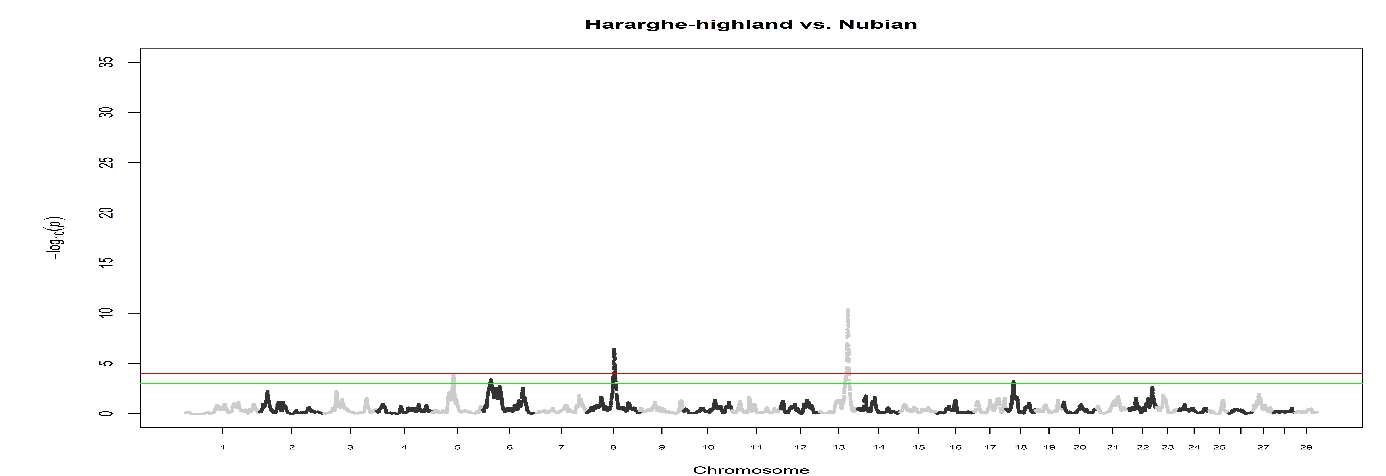


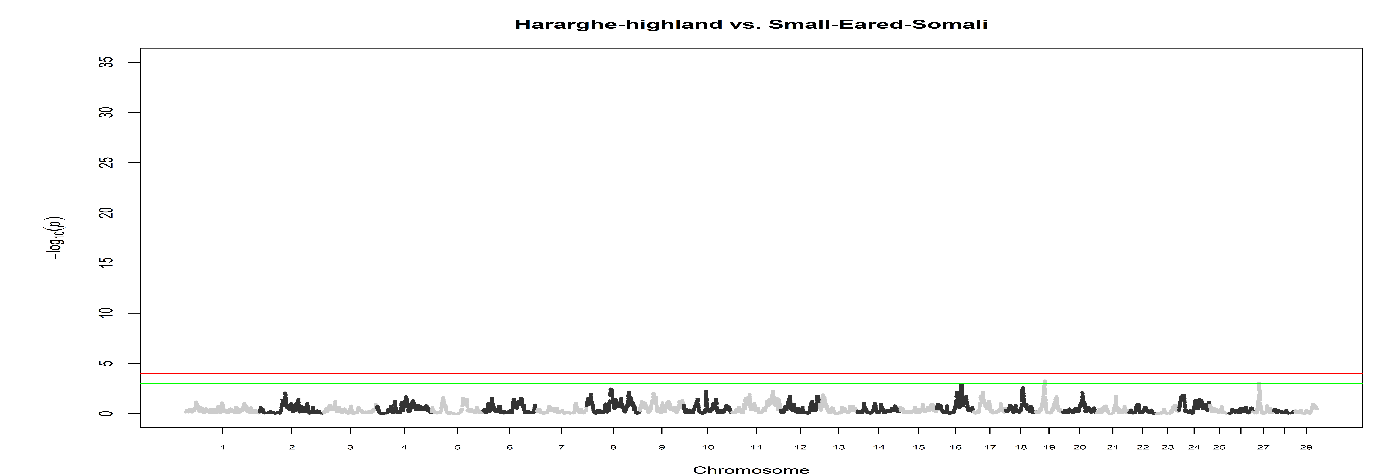


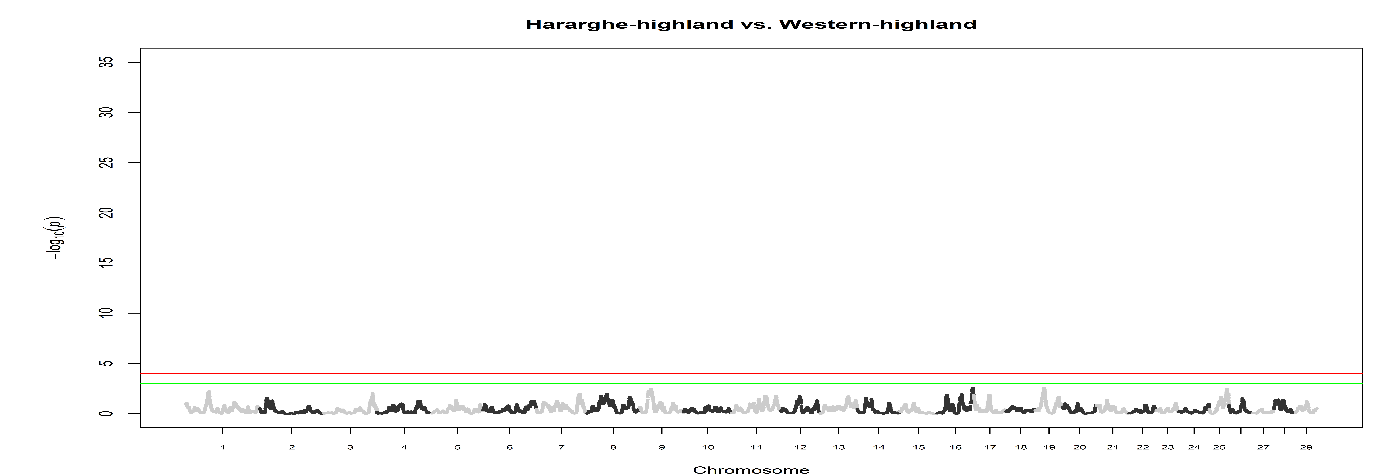


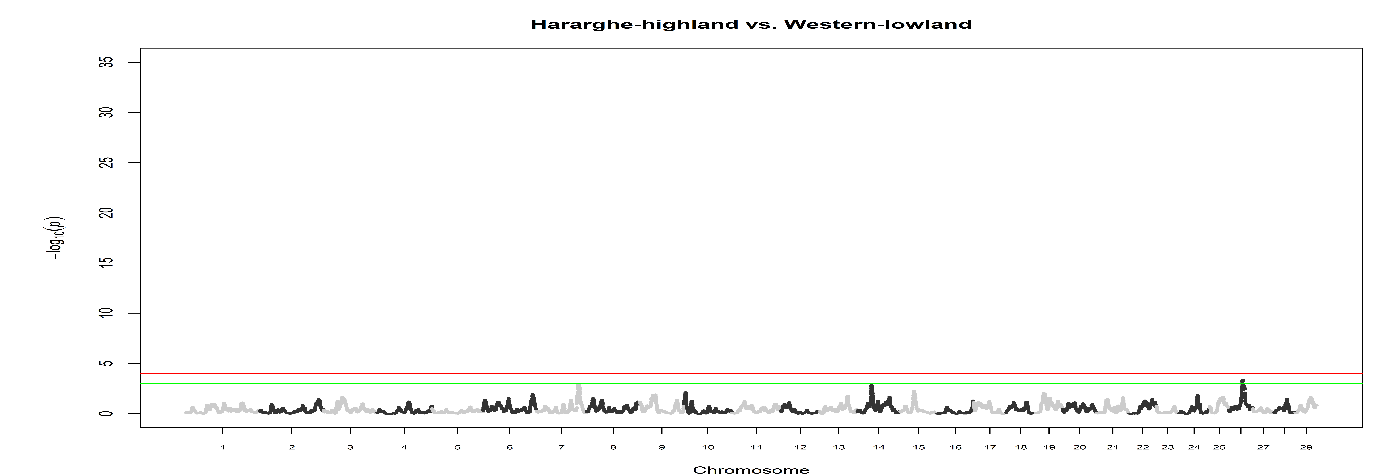


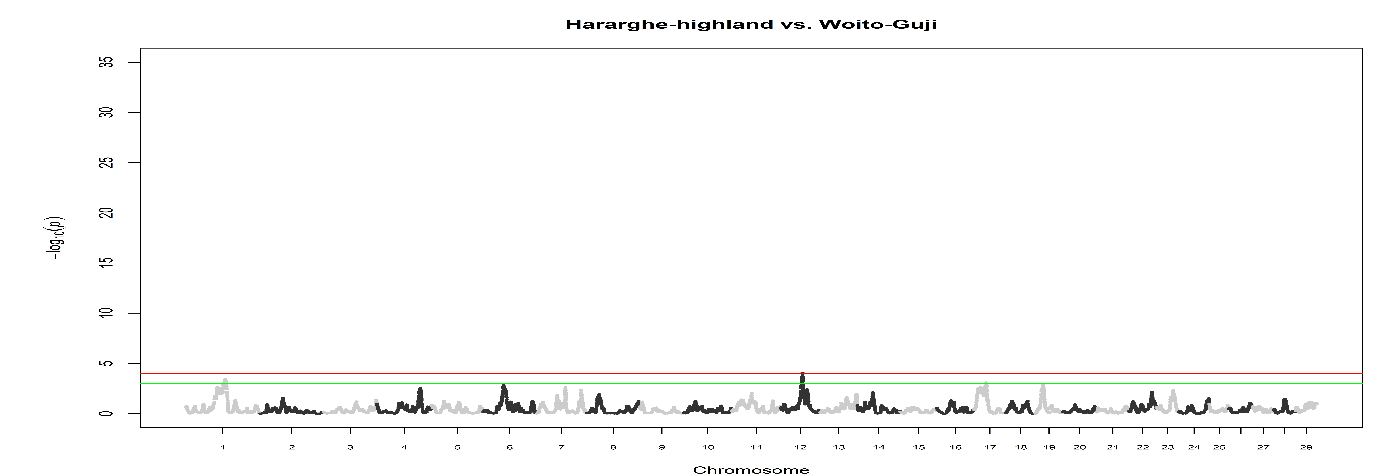


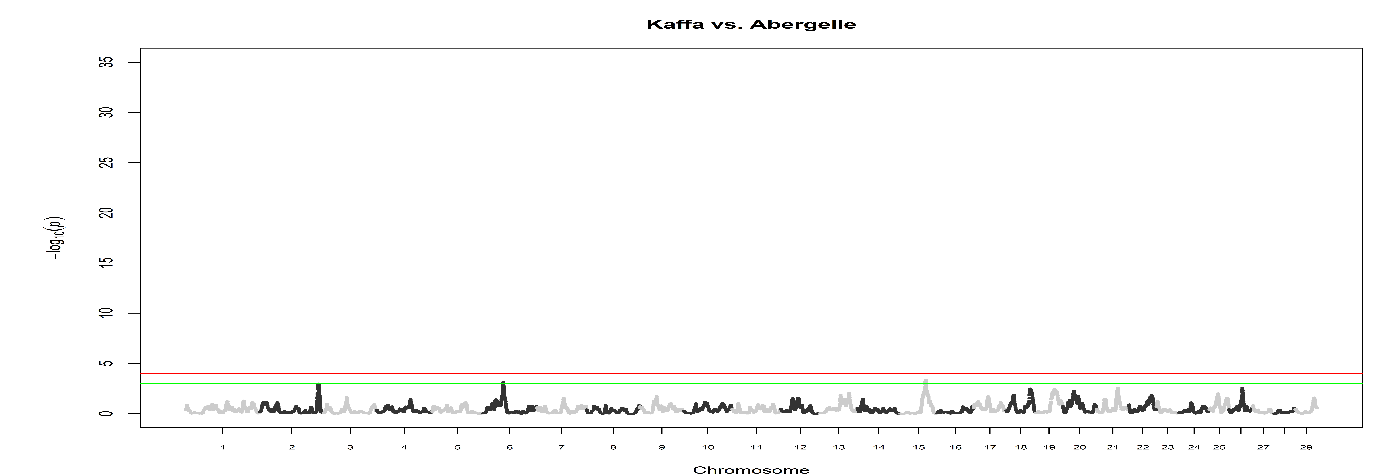


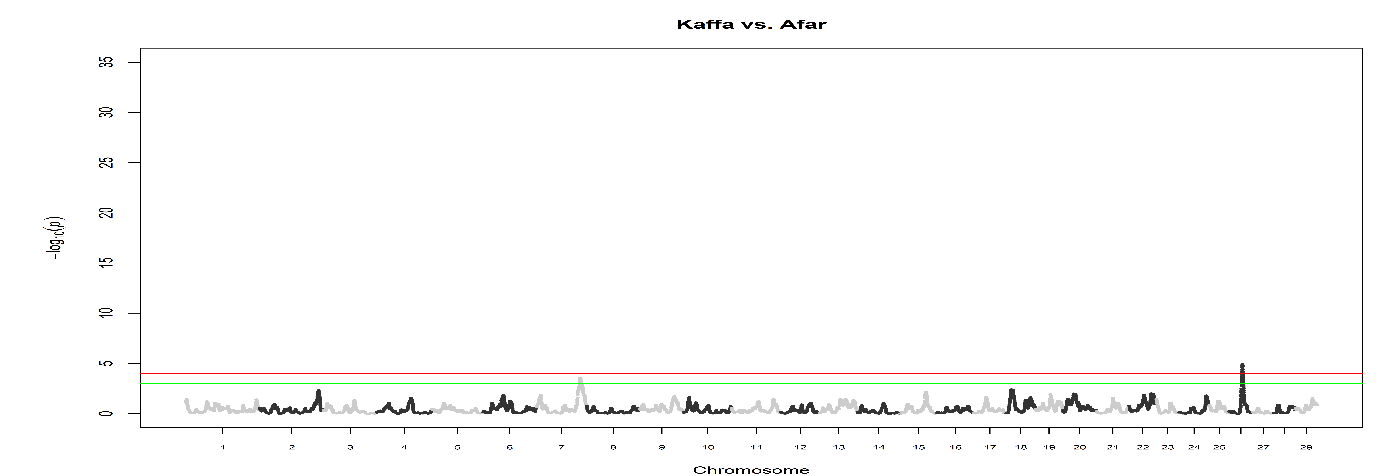


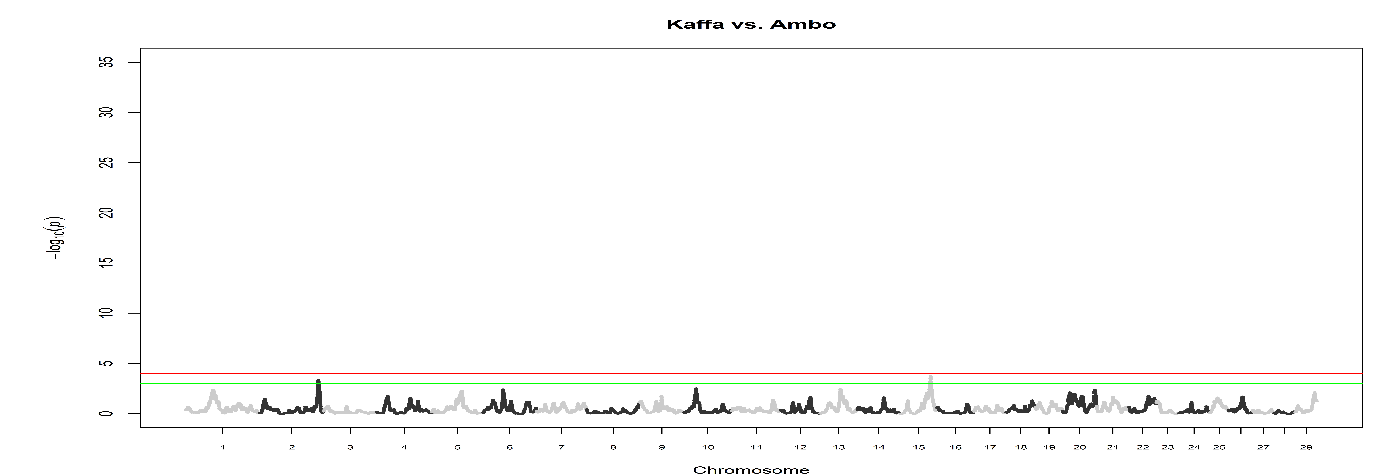


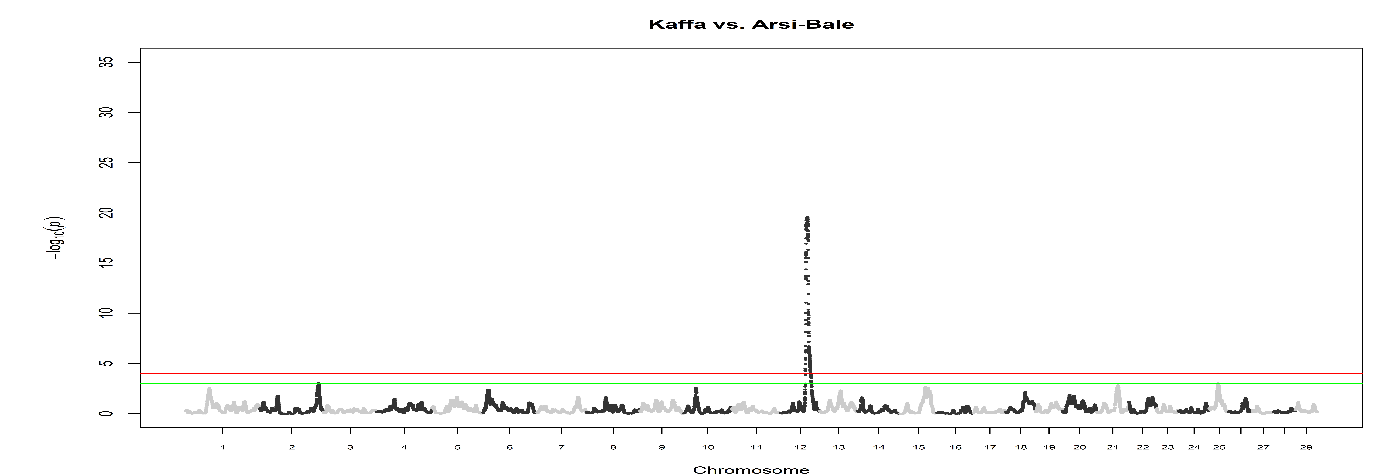


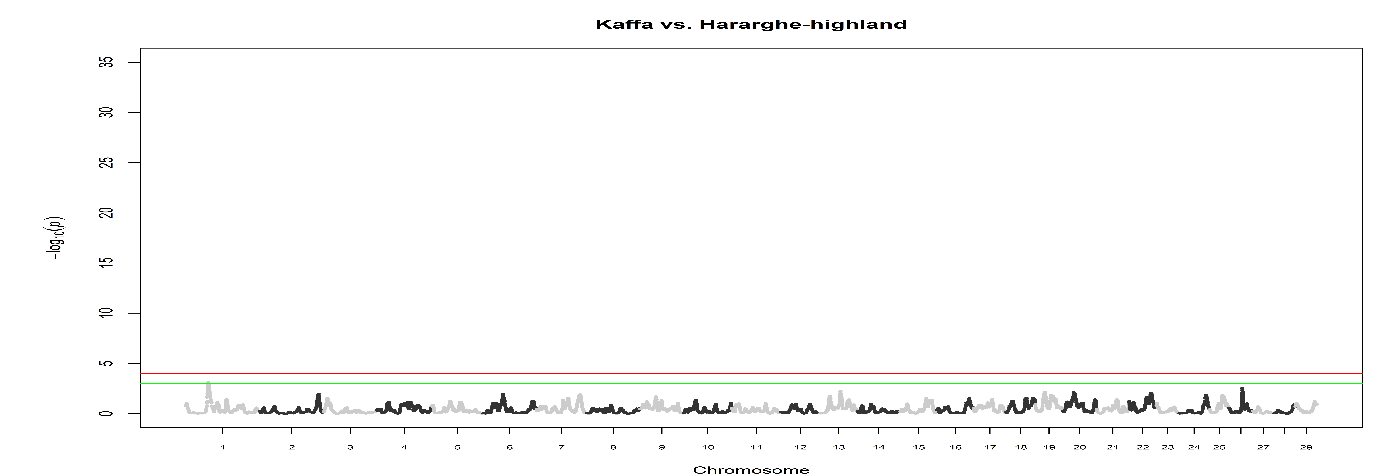


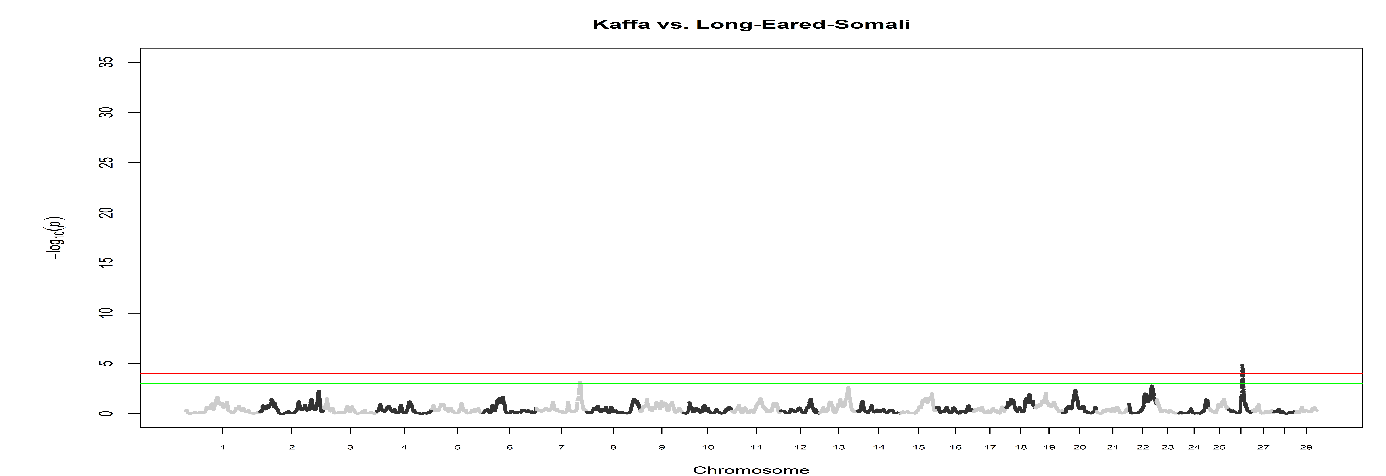


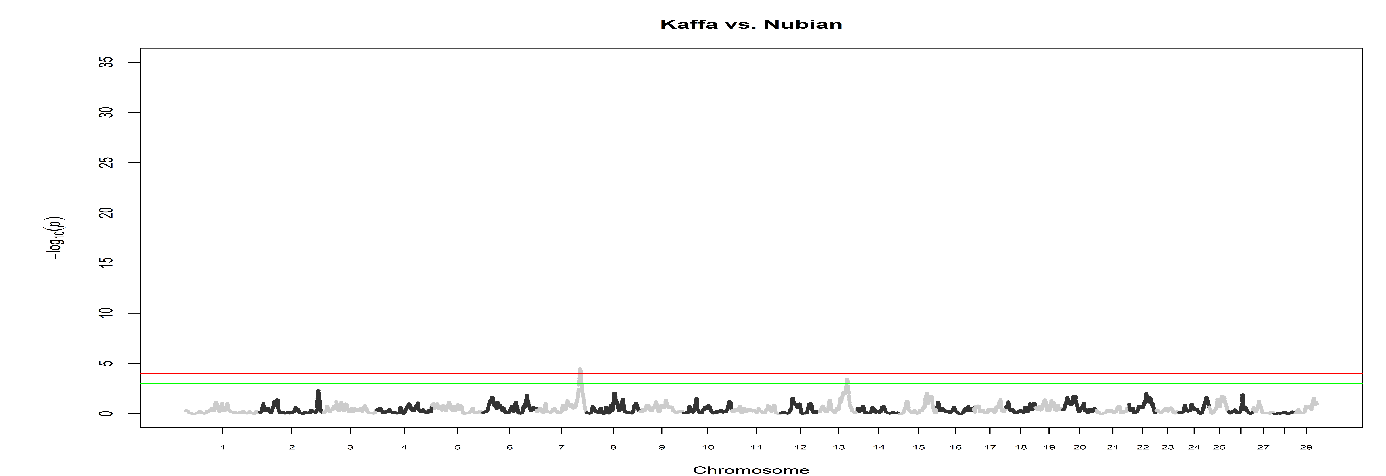


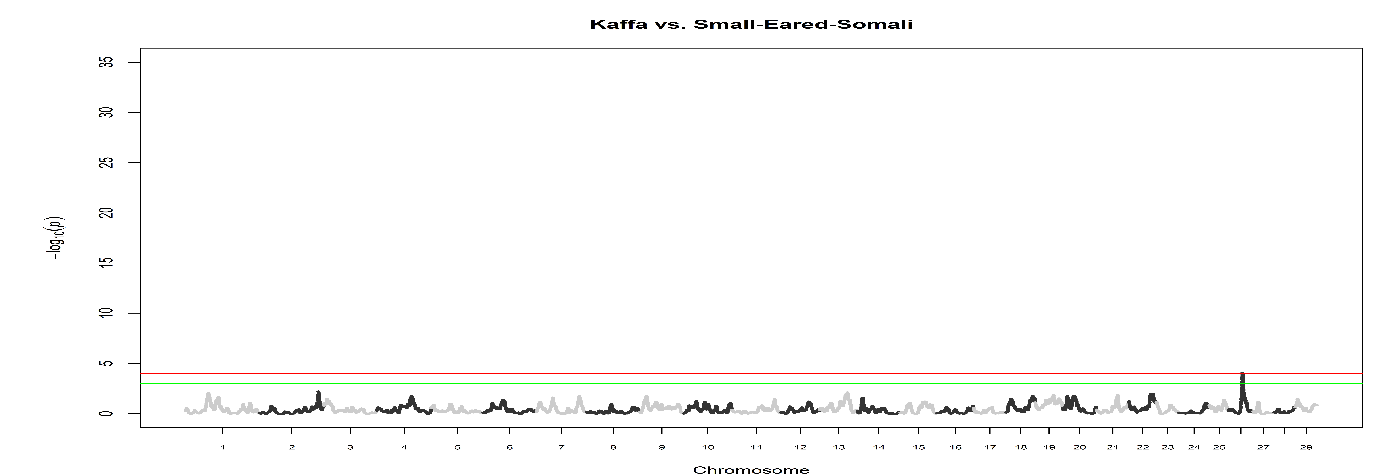


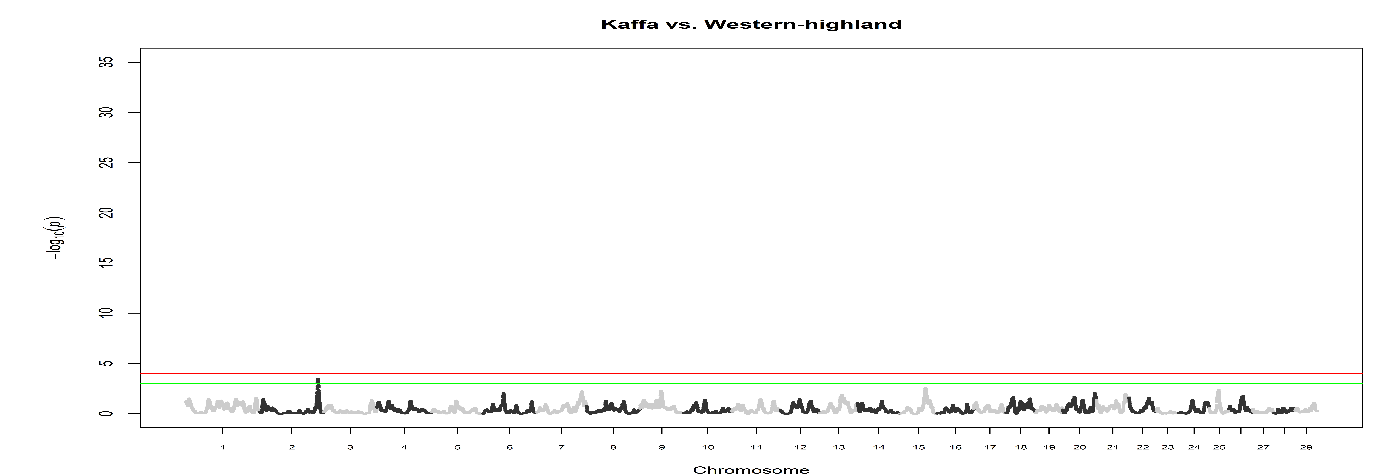


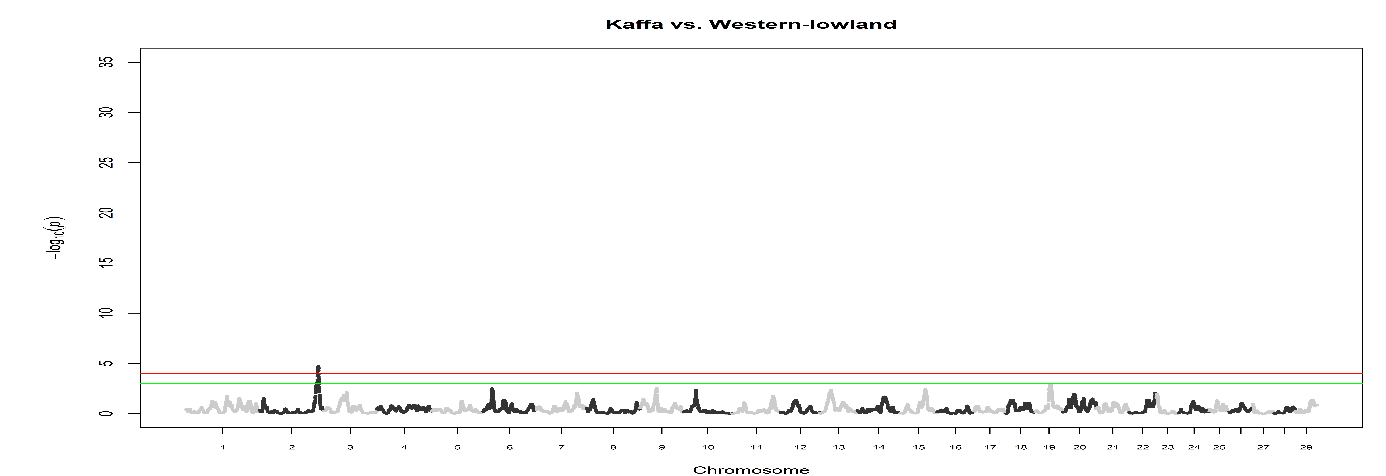


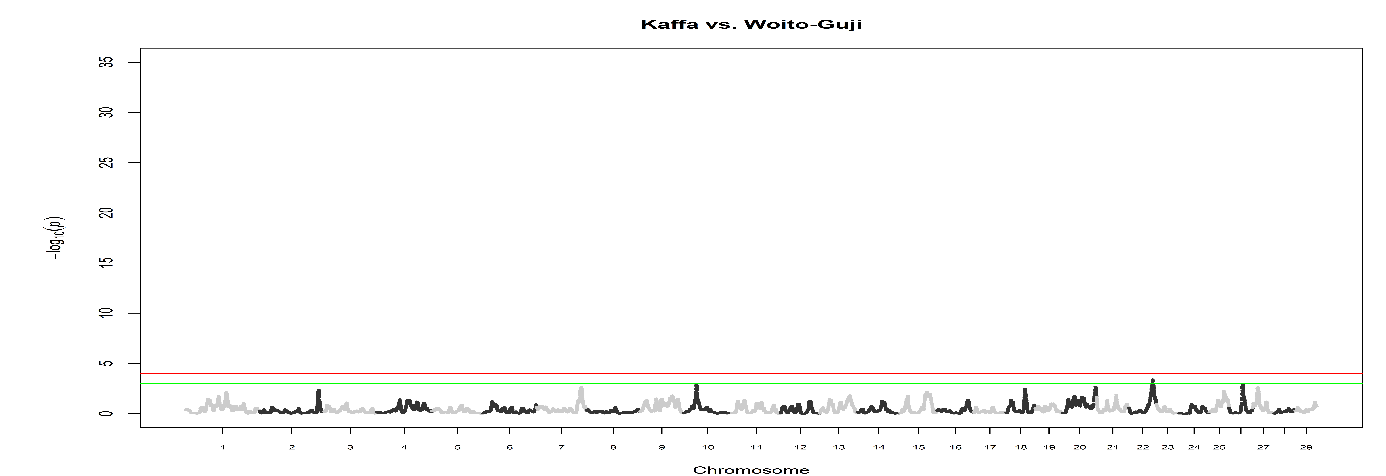


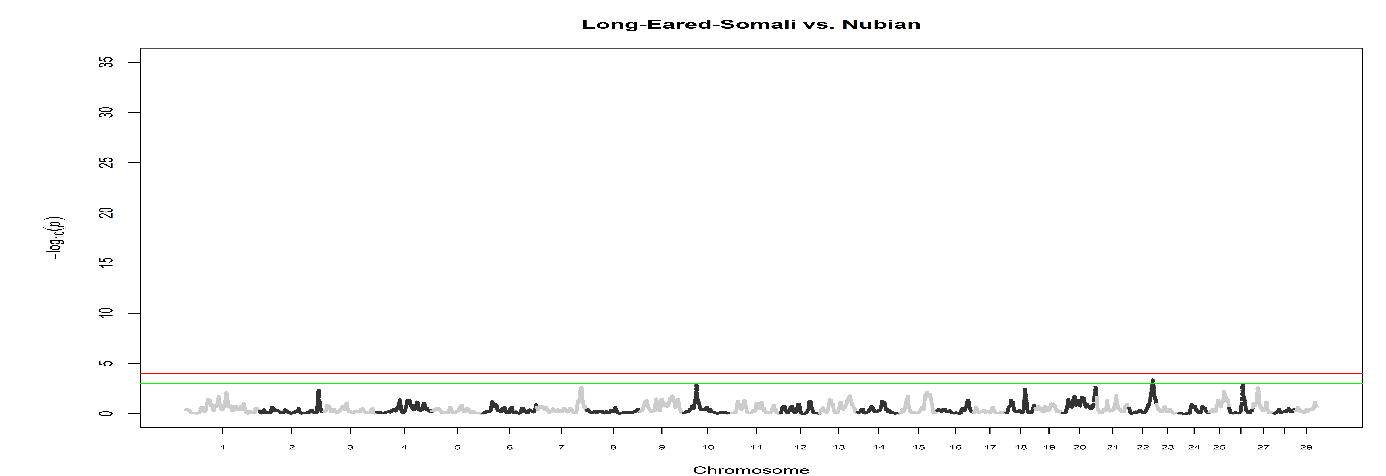


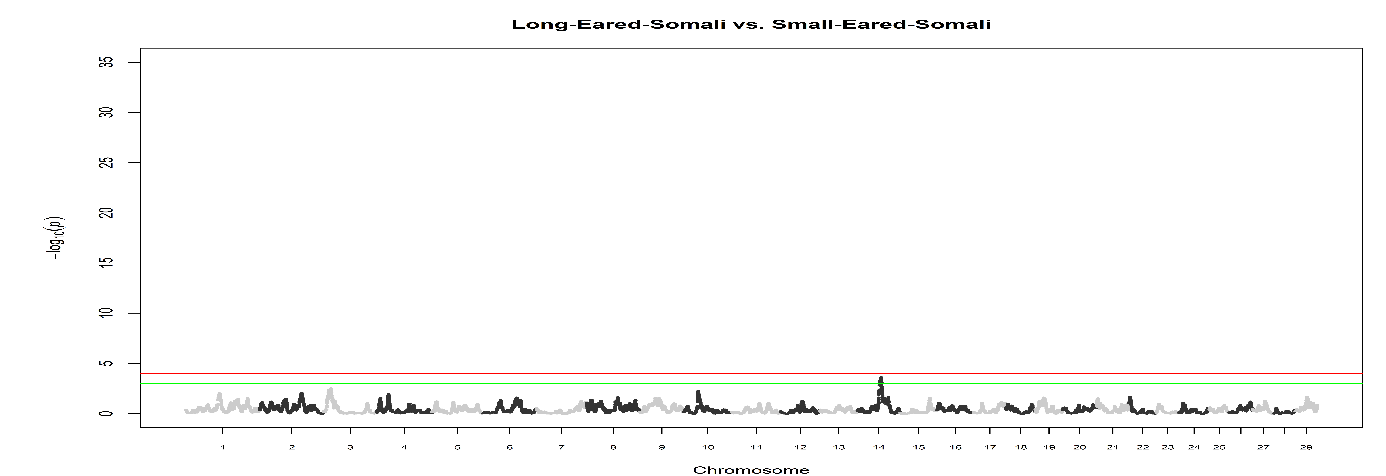


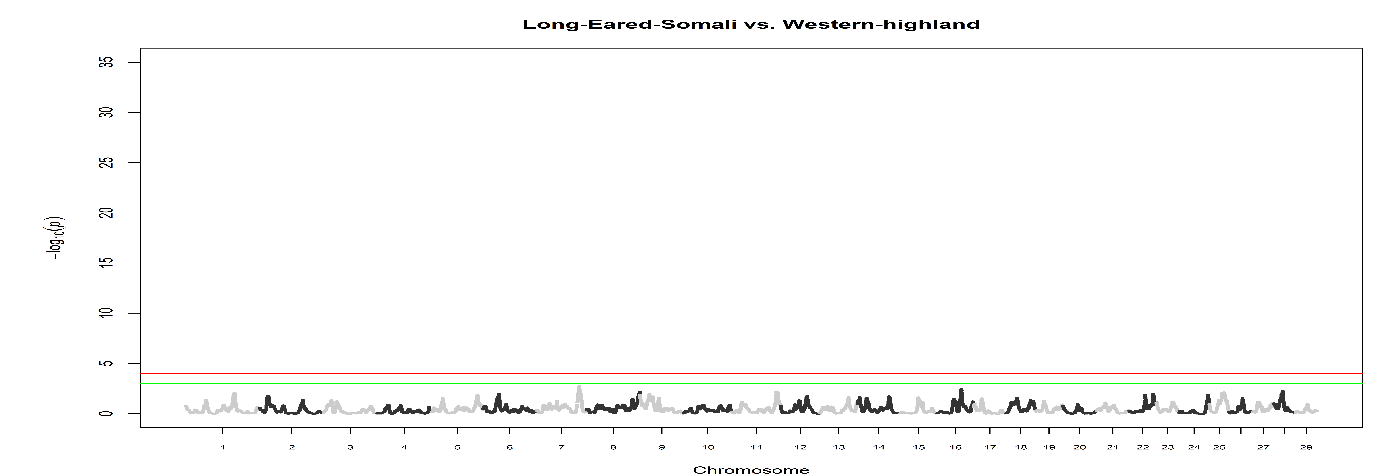


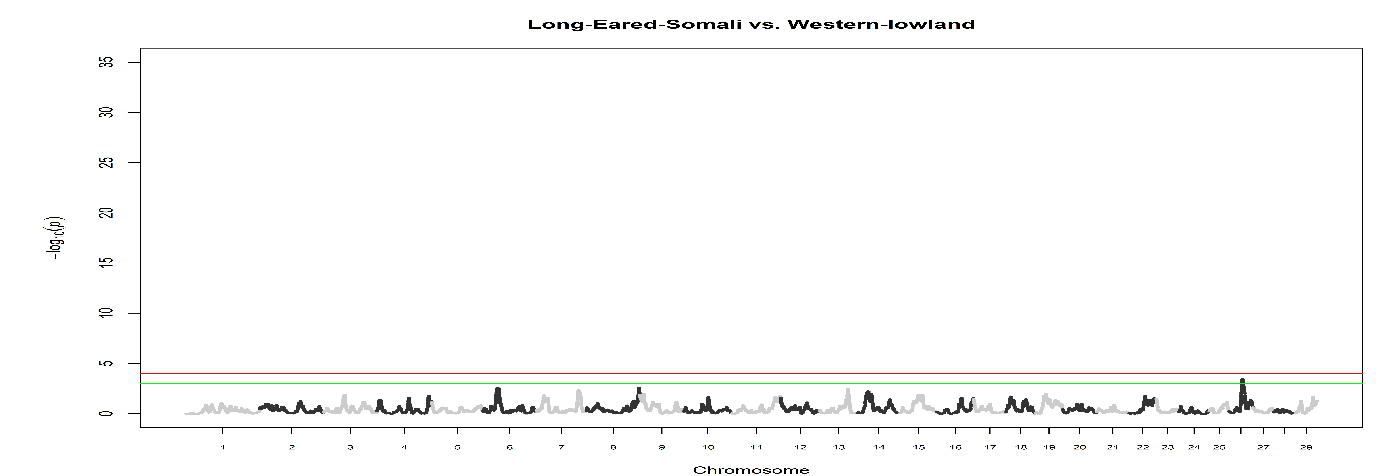


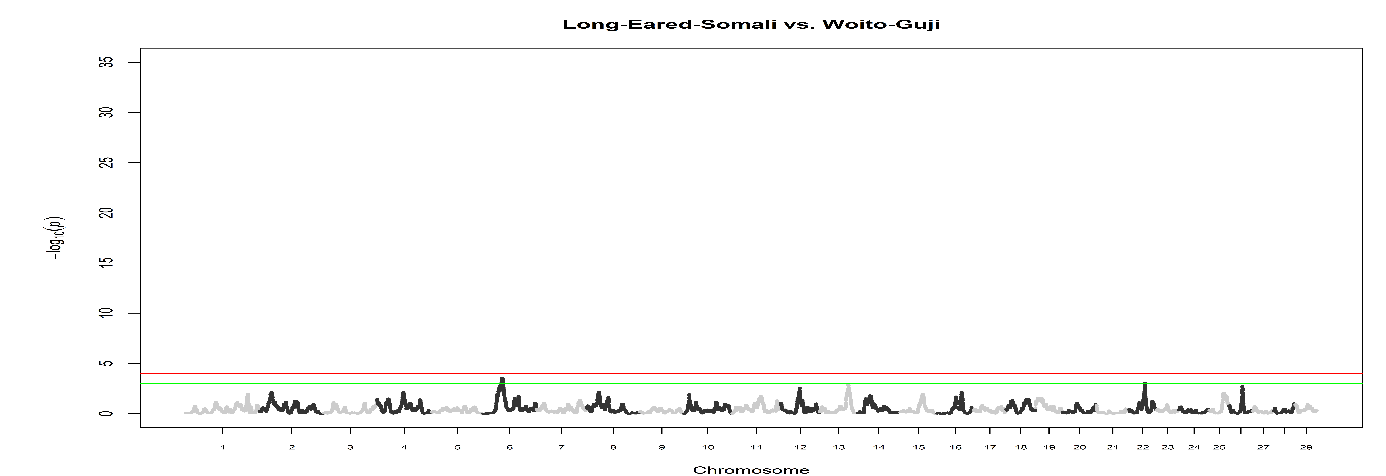


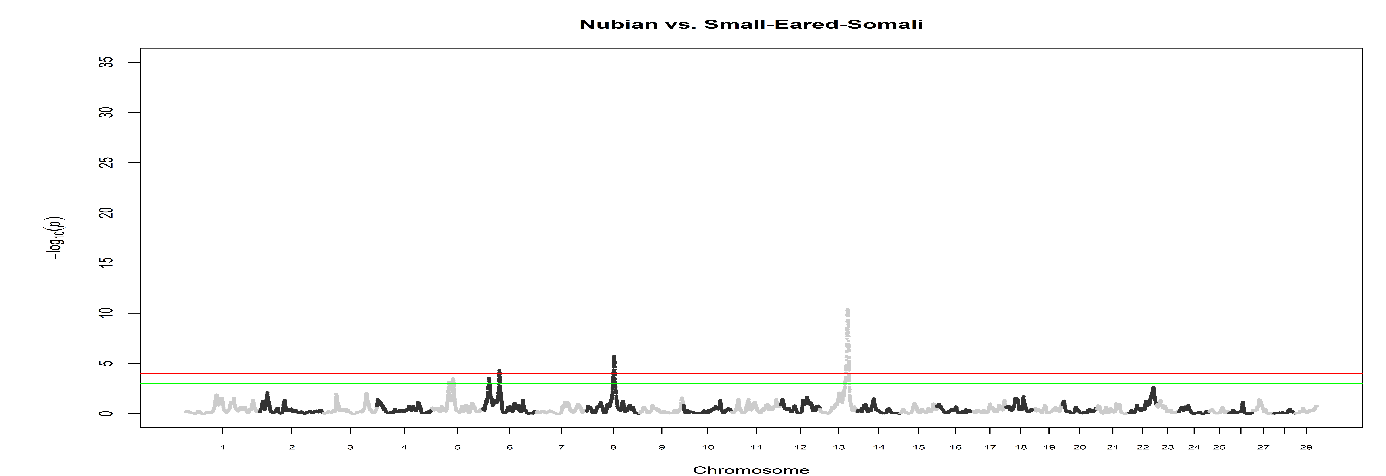


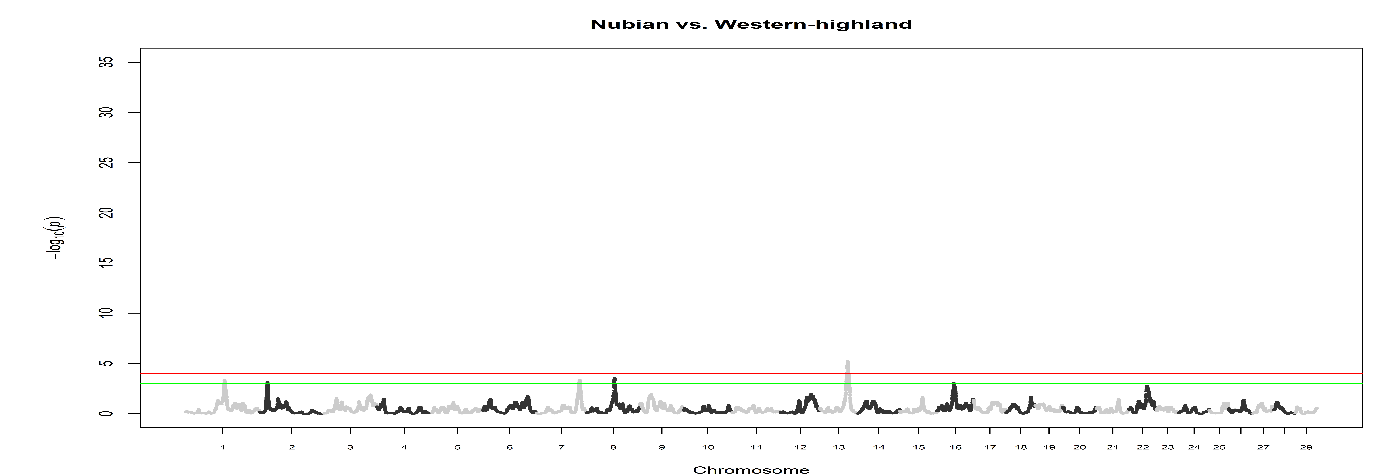


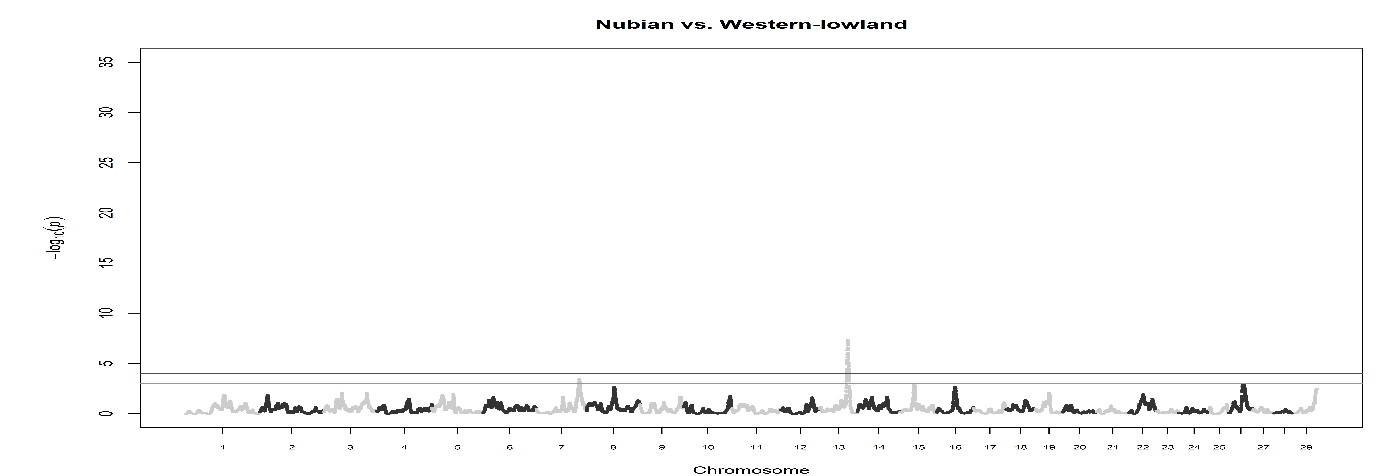


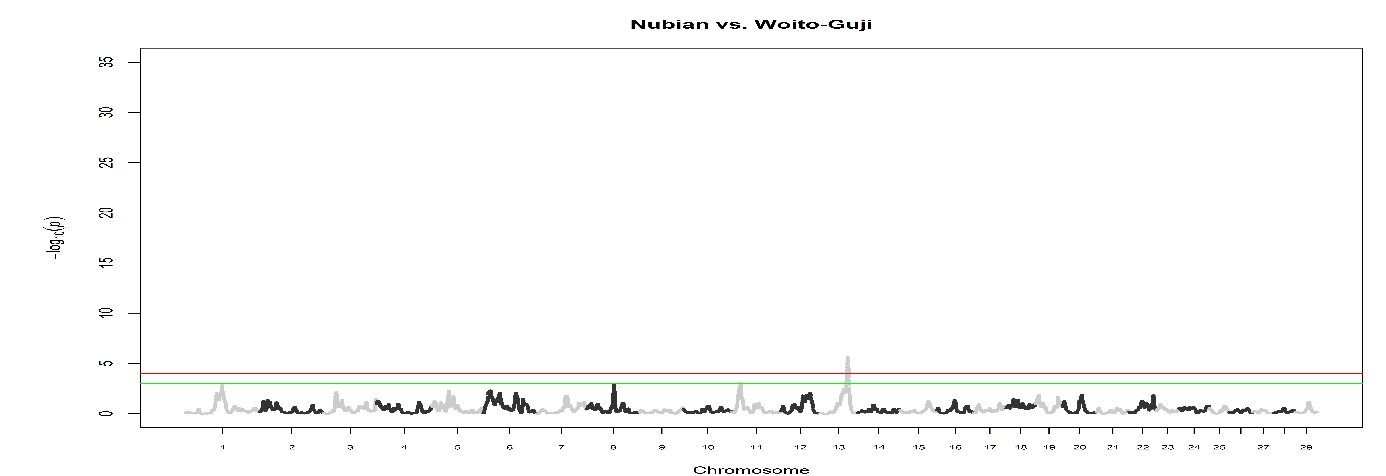


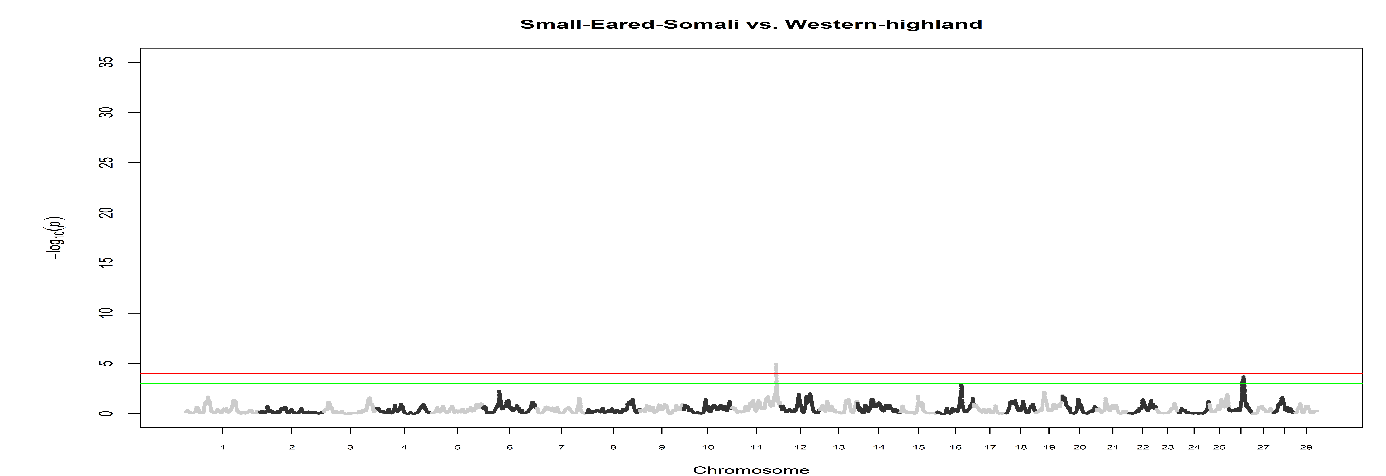


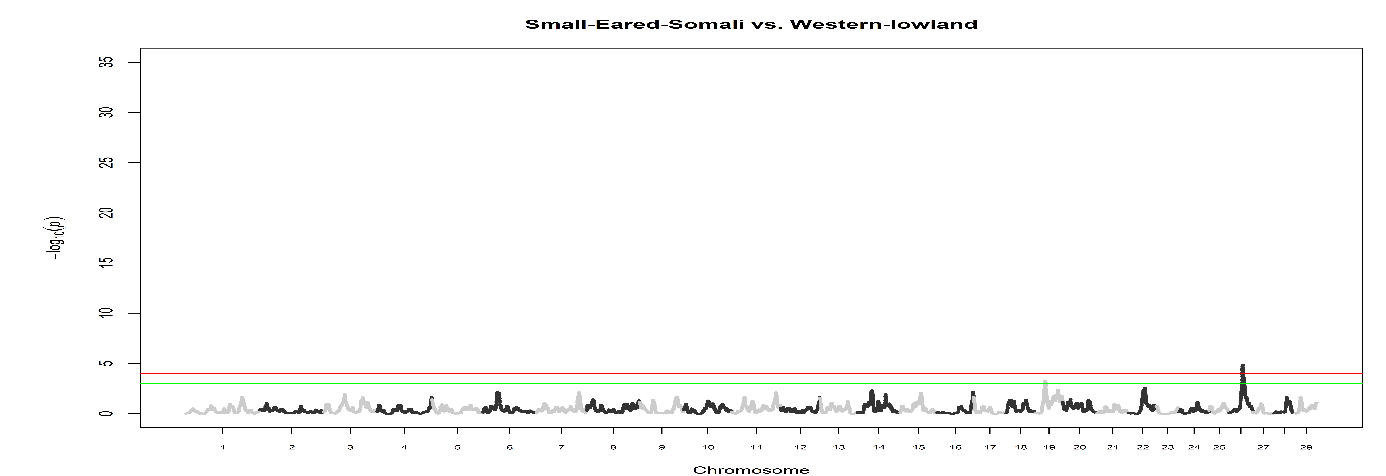


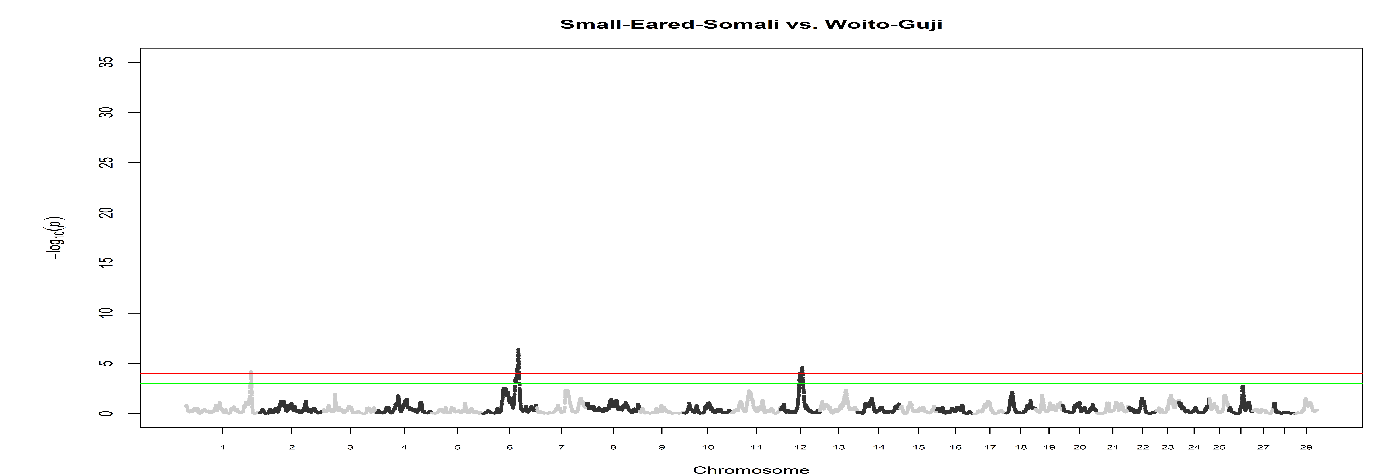


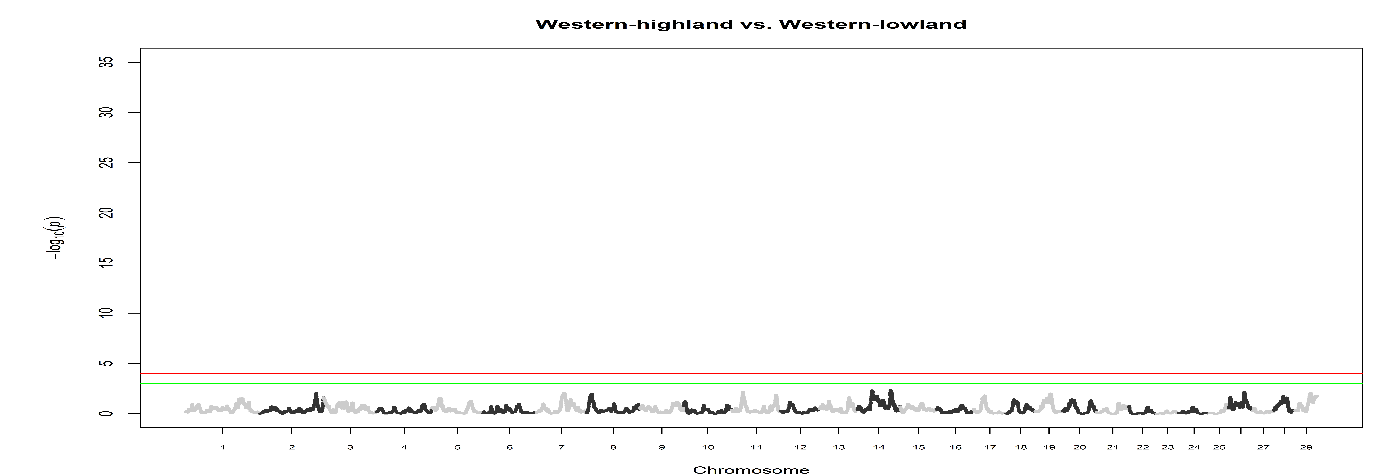


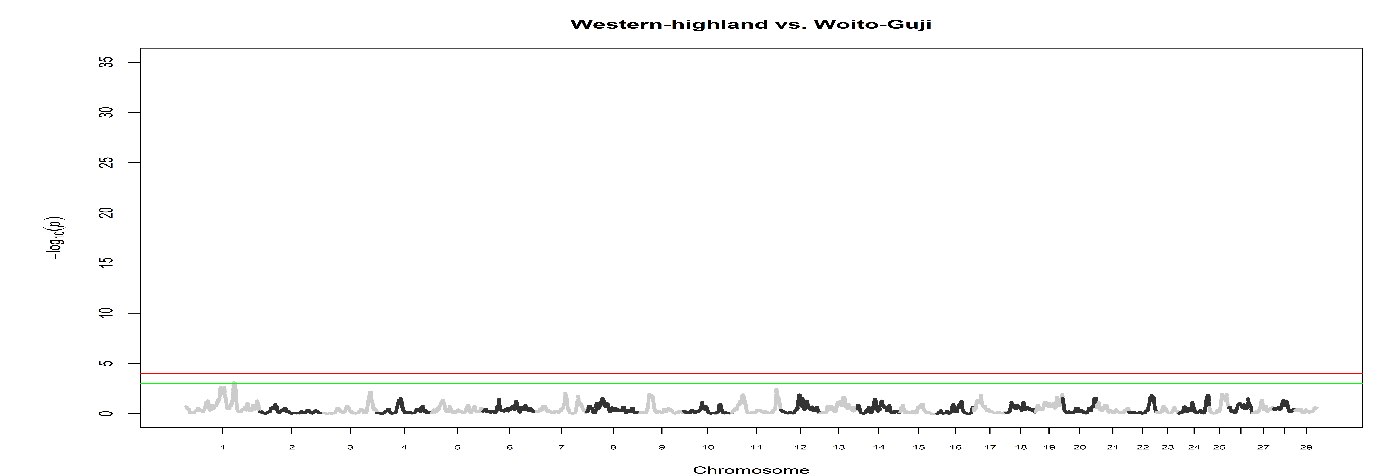


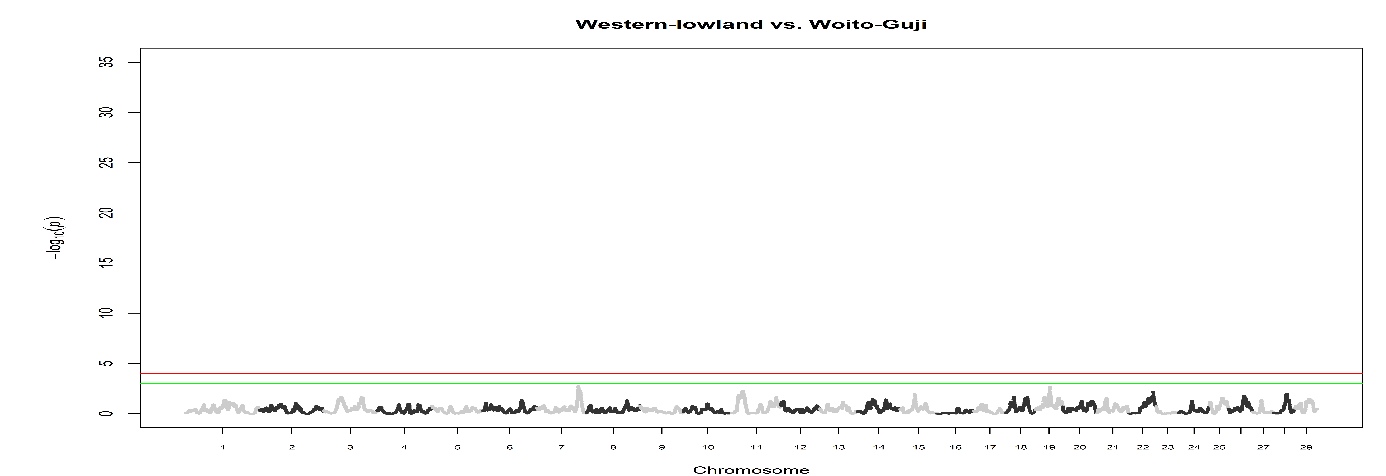

Supplement: Supplementary file 2 — Fig S3 [file EVA-14-1716-s001.docx]
