# Supplementary material for: Ethiopian indigenous goats offer insights into past and recent demographic dynamics and local adaptation in sub‐Saharan African goats
Source: Evol Appl. 2021 Jun 15;14(7):1716–31. doi: 10.1111/eva.13118 (PMC8287980; doi:10.1111/eva.13118)
Supplement: Supplementary file 4 — Tables S1‐S2 [file EVA-14-1716-s002.docx]

Supplementary Table S1 The candidate regions and genes identified by hapFLK and XP-EHH in Arsi-Bale and Nubian goats

| Population | Chromosome | Region (bp) | Position of the top SNP (bp) | Gene closest to the top SNP | Genes |
| --- | --- | --- | --- | --- | --- |
| Arsi-Bale regions | 6 | 8,533,828-17,357,213 | 14,116,486 | ENSCHIG00000007265  (lincRNA) | TRAM1L1, NDST4, UGT8, ARSJ, ANK2, NEUROG2, ALPK1, TIFA, AP1AR, PITX2, ENPEP, ELOVL6, LRIT3, RRH, PLA2G12A, CASP6, MCUB, SEC24B, COL25A1, ETNPPL |
|  | 12 | 53,982,423 – 62,977,761 | 57,727,964 | FRY | USP12, RPL21, RASL11A, LNX2, POLR1D, GSX1, PDX1, CDX2, FLT3, PAN3, FLT1, SLC46A3, MTUS2, SLC7A1, UBL3, KATNAL1, HMGB1, USPL1, ALOX5AP, MEDAG, TEX26, HSPH1, B3GLCT, RXFP2, FRY, ZAR1L, BRCA2, N4BP2L1,, PDS5B, KL, STARD13, RFC3, NBEA, MAB21L1, DCLK1, SPART, CCNA1, SERTM1, RFXAP, SMAD9, EXOSC8, ALG5, SUPT20H, POSTN, TRPC4 |
| Nubian regions | 8 | 54,717,960-63,857,851 | 59,231,221 | UNC13B | TLE1, PHF24, DNAJB5, VCP, FANCG, PIGO, STOML2, FAM214B, UNC13B, RUSC2, FAM166B, TESK1, CD72, SIT1, CCDC107, ARHGEF39, CA9, TLN1, CREB3, GBA2, RGP1, MSMP, NPR2, HINT2, FAM221B, TMEM8B, OR13J1, SPAAR, OR2S2, RECK, CCIN, GNE, RNF38, MELK, PAX5, ZCCHC7, GRHPR, ZBTB5, POLR1E, FBXO10, TLE4, FRMPD1, TRMT10B, EXOSC3, DCAF10, IGFBPL1, TDRD7, TMOD1, TSTD2, NCBP1, XPA, FOXE1, TRMO, HEMGN, NANS, TRIM14, TBC1D2, GABBR2, ANKS6, GALNT12, COL15A1, TGFBR1 |
|  | 13 | 49,975,695-67,760,577 | 62,821,046 | CHMP4B | PMEPA1, PCK1, CTCFL, RBM38, RAE1, SPO11, BMP7, TFAP2C, RTF2, GCNT7, CASS4, CSTF1, AURKA, SIRPB2, NSFL1C, SDCBP2, SNPH, RAD21, C20orf202, TMEM74B, PSMF1, RSPO4, ANGPT4, FAM110A, SLC52A3, SCRT2, SRXN1, TCF15, TBC1D20, RBCK1, TRIB3, NRSN2, SOX12, ZCCHC3, C20orf96, DEFB129, DEFB127, DEFB126, DEFB116, DEFB119, DEFB124, REM1, HM13, ID1, TPX2, MYLK2, FOXS1, DUSP15, TTLL9, PDRG1, XKR7, CCM2L, HCK, TM9SF4, PLAGL2, POFUT1, KIF3B, ASXL1, NOL4L, DNMT3B, EFCAB8, SUN5, BPIFB2, BPIFB6, BPIFB3, BPIFB4, BPIFA3, BPIFA1, BPIFB1, CDK5RAP1, SNTA1, NECAB3, ACTL10, E2F1, PXMP4, ZNF341, CHMP4B, RALY, ASIP, AHCY, ITCH, DYNLRB1, MAP1LC3A, PIGU, TP53INP2, NCOA6, GGT7, ACSS2, GSS, MYH7B, TRPC4AP, EDEM2, PROCR, MMP24, EIF6, FAM83C, UQCC1, GDF5OS,, GDF5, CEP250, ERGIC3, SPAG4, CPNE1, RBM12, ROMO1, PHF20, SCAND1, CNBD2, EPB41L1, AAR2, DLGAP4, MYL9, TGIF2, SLA2, NDRG3, DSN1, SOGA1, TLDC2, SAMHD1, RBL1, MROH8, RPN2, GHRH, MANBAL, SRC, NNAT, BLCAP, CTNNBL1, VSTM2L, TTI1, RPRD1B, TGM2, KIAA1755, BPI, LBP, RALGAPB, ADIG, SLC32A1, ACTR5, HAO1, ADRA1D, SMOX, RNF24, PANK2, MAVS, AP5S1, CENPB, SPEF1, C20orf27, HSPA12B, SIGLEC1, ADAM33, GFRA4, ATRN, C20orf194, SLC4A11, ITPA, DDRGK1, LZTS3, FASTKD5, UBOX5, AVP, OXT, PTPRA, VPS16, PCED1A, TMEM239, C20orf141, CPXM1, EBF4, IDH3B, NOP56, TMC2, SNRPB, TGM6, STK35, PDYN, GINS1, MYT1, NPBWR2, OPRL1, LKAAEAR1, RGS19, TCEA2, SOX18, PRPF6, SAMD10, UCKL1, ZNF512B, TPD52L2, ABHD16B, ZBTB46, SLC2A4RG, ARFRP1, TNFRSF6B, STMN3, GMEB2, FNDC11, SRMS, EEF1A2, KCNQ2, CHRNA4, ARFGAP1, BIRC7, YTHDF1, BHLHE23, SLC17A9, GID8, DIDO9, TCFL5, OGFR, NTSR1, SLCO4A1, GATA5, RBBP8NL, CABLES2, RPS21, LAMA5, ADRM1, OSBPL2, HRH3, MTG2, SS18L1, PSMA7, TAF4, CDH4, CDH26, FAM217B, PPP1R3D, SYCP2, PHACTR3, EDN3, ZNF831, TUBB1, CTSZ, NPEPL1, STX16, APCDD1L, VAPB, RAB22A, C20orf85, PPP1R16B, FAM83D, DHX35 |

Supplementary Table S2 Functions of genes identified in candidate selection signature regions in Arsi-Bale and Nubian goat populations

| **Population** | **Candidate gene(s)** | **Function** | **Reference** |
| --- | --- | --- | --- |
| Arsi-Bale | *BRCA2*, *RAD5A*, *PDS5B*, *RAD51* | DNA repair processes enhancing genome integrity and stability | Prakash et al. (2015); Couturier et al. (2016); Chudasama et al. (2018) |
|  | *CDX2*, *SMC3* | Regulation of transcription, intestinal homeostasis and inflammation | Coskun et al. (2011); Dorsett (2011) |
|  | *FLT3/CD135* | Mediates cell survival, cell proliferation, and differentiation of hematopoietic progenitor cells, Cytokine receptors | Kazi and Rönnstrand (2019) |
|  | *CDX2*, *HMGB1*, *ETNPPL1*, *DCLK1*, *STARD13*/*CCNA1*, *NBEA*, *SMAD9* | Female reproduction | Brinkhof et al. (2015); Sun et al. (2015); Shen et al. (2017); Kfir et al. (2018) |
|  | *FLT1*, *PITX2*, *Neurog2* | Embryogenesis | Minocha et al. (2017); Marques et al. (2018) |
|  | *FLT1*, *SLC7A1*, *CDX2* | Sperm maturation | Chu et al. (2015) |
|  | *SMAD9* | Wool traits, epithelium development | Seroussi et al. (2017) |
|  | *KATNAI1* | Fiber diameter |  |
|  | *FRY* | Coat pigmentation |  |
|  | *RXFP2* | Horn phenotype | Montgomery et al. (1996); Wiedemar and Drogemuller (2015); Seroussi et al. (2017); |
|  | *NBEA* | Adaptation to feed stress, body fat mass, feeding behavior, body mass index | Olszewski et al. (2012); Seroussi et al. (2017) |
|  | *ANK2*, *ELOVL6*, *FLT1* | Intramuscular fatty acid composition | Ramayo-Caldas et al. (2014) |
|  | *MTUS2*, *ELOVL6*, *POSTN*, *PITX2* | Skeletal muscle development, growth, differentiation and characteristics | Campbell et al. (2012); Ovilo et al. (2014); |
|  | *ALOX5AP* | Climate adaptation | Lv et al. (2014); Seroussi et al. (2017) |
| Nubian | *PXMP4*, *PIGU*, *ZNF341*, *NCOA6*, *ACSS2*, *E2F1* | Synthesis of bovine milk fatty acids/milk fat composition | Knutsen et al. (2018); Olsen et al. (2017) |
|  | *PROCR*, *AURKA*, *FOXS1*, *CD72*, *AVP* | Adult mammary gland development | Bach et al. (2017) |
|  | *CTCFL*, *SPO11*, *RBM38*, *PMEPA1* | Fertility associated phenotypes | Jabbari et al. (2018) |
|  | *CTNNBL1*, *NNAT*, *BLCAP*, *CPNE1*, *SPAG4* | Calving traits e.g. calving ease | Frischknecht et al. (2017) |
|  | *MYLK2*, *MYL9*, *MYH7B*, *TNFRSF6B*, | Muscle fiber development | Ye et al. (2017) |
|  | *GNE*, *RNF38*, *TRIM14*, *NANS* | Ear lobe colour in chicken | Hollmann et al. (2017); Nie et al. (2016). |
|  | *PCK1*, *CTCFL*, *SPO11*, *BMP7* | Environmental stress, control of cytokine production, delayed apoptosis |  |
|  | *PLAGL2*, *SRXN1* | Associated with oxidative stress responding regulator | Mwacharo et al. (2017) |
|  | *FAM166B*, *WDR32*, *TLE4*, *NNAT* | Related to growth and lipid deposition | Tao et al. (2017) |
|  | *ZBTB46*, *ARFRP1*, *STMN3*, *GMEB2*, *C20orf195*, *SRMS*, *EEF1A2* | Important economic traits in sheep and genetic basis of adaptation to different ecological environments | Liu et al. (2016) |
|  | *CEP250* | Cell growth and proliferation, rib eye area |  |
|  | *EPB41L1* | Backfat thickness, growth regulation |  |
|  | *PROCR* | Regulate other genes functioning towards bone growth |  |
|  | *ERGK3* | Related to cell growth and proliferation, growth and meat quality traits of Brazilian Nelore cattle | Mudadu et al. (2016) |
|  | *TDRD7* | Mitochondrial homeostasis |  |
|  | *NNAT* | Imprinted brain development |  |
|  | *RAD21* | Mitosis and chromatin organization function |  |
|  | *SOX18* | Transcription factor activity | Hao et al. (2016) |
|  | *HCK*, *PROCR*, *CTSZ* | Classical innate immune inflammatory responses and fever induction |  |
|  | *PDYN* | Closely interconnected with multiple immune-related proteins | Fang et al. (2016) |
|  | *SLC4A11* | Candidate for bovine mastitis |  |
|  | *BMP7*, *PCK1* | Associated with diabetes and obesity (9, 10, Hart et al. 2007) | Gularte-Merida et al. (2015) |
|  | *VAPB* | Encodes secondary signaling cascades and transport proteins |  |
|  | *VSTM2L* | Hot carcass weight | Espigolan et al. (2015) |
|  | *BLCAP*, *NNAT*, *CTNNBL1*, *TGM2* | Basal metabolism |  |
|  | *SRXN1*, *PLAGL2* | Transcripts associated with oxidative stress | Henry et al. (2015) |
|  | *VCP* | Elevated during energy deficit |  |
|  | *HRH3* | G-protein coupled receptor |  |
|  | *PDYN* | Increase appetite |  |
|  | *GDF5*, *UQCC* | Contain polymorphisms that are associated with stature in humans (55), body size determination in European Bos Taurus (56) | Sorbolini et al. (2015) |
|  | *MYL9* | Muscle biology |  |
|  | *ADIG* | Lipid and carbohydrate metabolism |  |
|  | *PROCR* | Immune and acute inflammatory responses |  |
|  | *GHRH* | Release of growth hormone |  |
|  | *ACSS2* | Role in lipid metabolism |  |
|  | *VCP*/*r97* | Implicated in endoplasmic reticulum stress | Nayak et al. (2014) |
|  | *XPA* | Response to ionizing radiation stress |  |
|  | *EDN3* | Possible molecular marker for dark skin colour in poultry; Association between EDN3 and melanoblast proliferation has been previously reported (39-41) | Han et al. (2014) |
|  | *IGFBPL1* | Associated with milk production performance in cattle through IGFs (40) |  |
|  | *FANCG*, *XPA* | DNA repair, genome stability and integrity |  |
|  | *PLUNC* | PLUNC proteins are structural homologous to innate defense molecules LBP and BPI |  |
|  | *CTSZ* | Immune response | Han et al. (2014); Wheeler et al. (2007) |
|  | *CTCFL*, *TGFBR1* | Spermatogonial stem cell development | Guo et al. (2017) |
|  | *BPIFA1*, *BPIFA3*, *BPIFB4*, *BPIFB6*, *BPIFB1* | Antimicrobial defense, innate defense proteins | Kuntova et al. (2018); De Smet et al. (2018) |
|  | *RALY*, *AHCY*, *EDEM2*, *EIF6*, *UQCC*, *MYH7B*, *FAM83C*, *NCOA5*, *GSS*, *PIGU*, *MMP24*, *GGT7* | Skin colour variation | Liu et al. (2015) |
|  | *MELK*, *GNE* | Linked to climate associated selection pressure | Mastrangelo et al. (2017) |
|  | *PAX5* | Associated with coat colour |  |

**References**

Bach, K., Pensa, S., Grzelak, M., Hadfield, J., Adams, DJ., Marioni, JC. & Khaled W.T. (2017). Differentiation dynamics of mammary epithelial cells revealed by single-cell RNA sequencing. *Nature Communications* 8, 2128.

Brinkhof, B., van Tol, H.T.A., Koerkamp, M.J.A.G., Riemers F.M., Ijzer, S.G., Mashayekhi, K. et al. (2015). A mRNA landscape of bovine embryos after standard and MAPK-inhibited culture conditions: a comparative analysis. *BMC Genomics* 16, 277.

Campbell, A.L., Eng, D., Gross, M.K. & Kioussi, C. (2012). Prediction of gene network models in limb muscle precursors. *Gene* 509, 16-23.

Chu, C., Zheng, G., Hu, S., Zhang, J., Xie, S., Ma, W. et al. (2015). Epididymal region-specific miRNA expression and DNA methylation and their roles in controlling gene expression in rats. *PLoS ONE* 10, e0124450..

Chudasama, P., Mughal, S.S., Sanders M.A., Hübschmann, D., Chung, I., Deeg, K.I., Wong, S-H., Rabe, S. et.al (2018). Integrative genomic and transcriptomic analysis of leiomyosarcoma. *Nature Communications*, 9, 144

Coskun, M. (2014). The role of CDX2 in inflammatory bowel disease. *Danish Medical Journal*, 61, B4820.

Couturier, A.M., Fleury, H., Patenaude, A-M., Bentley, V.L., Rodrigue, A. et al. (2016). Roles of APRIN (PDS5B) in homologous recombination and in ovarian cancer prediction. *Nucleic Acids Research* 44, 10879-10897.

Dorsett, D. (2011). Cohesin: genomic insights into controlling gene transcription and development. *Current Opinion in Genetics and Development.* 21, 199–206.

Espigolan, R., Baldi, F., Boligon, A.A., Souza, F.R.P., Fernandes Júnior, G.A., Gordo, D.G.M., Venturini, GC. et al. (2015). Associations between single nucleotide polymorphisms and carcass traits in Nellore cattle using high-density panels. *Genetics and Molecular Research* 14, 11133-11144.

Fang, L., Hou, Y., An, J., Li, B., Song, M., Wang, X., Sørensen, P., Dong, Y., Liu C. et al. (2016). Genome-wide transcriptional and post-transcriptional regulation of innate immune and defense responses of bovine mammary gland to *Staphylococcus aureus*. *Frontiers in Cellular and Infection Microbiology* 6, 193.

Frischknecht, M., Bapst, B., Seefried, F.R., Signer-Hasler, H., Garrick, D., Stricker, C., Intergenomics, C., Fries, R., Russ, I., Solkner, J. et al. (2017. Genome-wide association studies of fertility and calving traits in Brown Swiss cattle using imputed whole-genome sequences. *BMC Genomics* 18, 910.

Gularte-Mérida, R., Farber, C.R., Verdugo, R.A., Islas-Trejo, A., Famula, TR., Warden, C.H. & Medrano J.F. (2015). Overlapping mouse subcongenic strains successfully separate two linked body fat QTL on distal MMU 2. *BMC Genomics* 16, 16.

Guo, J., Grow, E.J., Yi, C., Mlcochova, H., Maher, G.J., Lindskog, C., Murphy, P.J., Wike, C.L., Carrell, D.T., Goriely, A. et al. (2017). Chromatin and single-cell RNA-Seq profiling reveal dynamic signaling and metabolic transitions during human spermatogonial stem cell development. *Cell Stem Cell* 21, 533-546

Han, R., Yang, P., Tian, Y., Wang, D., Zhang, Z., Wang, L., Li, Z., Jiang, R. & Kang, X. (2014). Identification and functional characterization of copy number variations in diverse chicken breeds. *BMC Genomics* 15, 934.

Hao, C., Gely-Pernot, A., Kervarrec, C., Boudjema, M., Becker, E., Khil, P. et al. (2016). Exposure to the widely used herbicide atrazine results in deregulation of global tissue-specific RNA transcription in the third generation and is associated with a global decrease of histone trimethylation in mice*. Nucleic Acids Research* 44, 9784-9802.

Henry, F.E., Sugino, K., Tozer, A., Branco, T. & Sternson, S.M. (2015). Cell Type-Specific Transcriptomics of hypothalamic energy-sensing neuron responses to weight-loss. *Elife* 4, e09800

Hollmann, A.K., Bleyer, M., Tipold, A., Neßler, J.N., Wemheuer, W.E., Schütz, E. & Brenig, B. (2017). A genome-wide association study reveals a locus for bilateral iridal hypopigmentation in Holstein Friesian cattle. *BMC Genetics* 18, 30.

Jabbari, K., Heger, P., Sharma, R. & Wiehe, T. (2018). The diverging routes of BORIS and CTCF: an interactomic and phylogenomic analysis. *Life* (Basel) 8, 4.

Kazi, J.U.& Rönnstrand L. (2019). FMS-like tyrosine kinase 3/FLT3: From basic science to clinical applications. *Physiological Reviews* 99, 1433-1466.

Kfir, S., Basavaraja, R., Wigoda, N., Ben-Dor, S., Orr, I. & Meidan, R. (2018). Genomic profiling of bovine corpus luteum maturation. *PLoS One* 13, e0194456.

Knutsen, T.M., Olsen, H.G., Tafintseva, V., Svendsen, M., Kohler, A., Kent, MP. & Lien S. (2018). Unravelling genetic variation underlying *de novo*-synthesis of bovine milk fatty acids. *Scientific Reports* 8, 2179.

Liu, Z., Ji, Z., Wang, G., Chao, T., Hou, L. & Wang J. (2016). Genome-wide analysis reveals signatures of selection for important traits in domestic sheep from different ecoregions. *BMC Genomics* 17, 863.

Lv, F.-H., Agha, S., Kantanen, J., Colli, L., Stucki, S., Kijas, J.W., Joost, S., Li, M.-H. & Ajmone Marsan, P. 2014. Adaptations to climate-mediated selective pressures in sheep. *Molecular Biology Evolution* 31, 3324-3343.

Marques, J.S., Teixeira, V., Jacinto, A. & Tavares AT. (2018). Identification of novel hemangioblast genes in the early chick embryo. *Cells* 7, 9.

Mastrangelo, S., Tolone, M., Sardina, M.T., Sottile, G., Sutera, A.M., Di Gerlando, R. & Portolano, B. (2017). Genome-wide scan for runs of homozygosity identifies potential candidate genes associated with local adaptation in Valle del Belice sheep. *Genetics Selection Evolution* 49, 84.

Minocha, S., Valloton, D., Arsenijevic, Y., Cardinaux, J.-R., Guidi, R., Hornung, J.-P. & Lebrand, C. (2017). Nkx2.1 Regulates the Generation of Telencephalic Astrocytes During Embryonic Development**.** *Scientific Reports* 7, 43093.

Mudadu, M.A., Porto-Neto, L.R., Mokry, F.B., Tizioto, P.C., Oliveira, P.S.N., Tulio, R.R., Nassu, RT., Niciura, S.C.M. et al. (2016). Genomic structure and marker-derived gene networks for growth and meat quality traits of Brazilian Nelore beef cattle. *BMC Genomics* 17, 235.

Mwacharo, J.M., Kim, E.-S., Elbeltagy, A.R., Aboul-Naga, A.M., Rischkowsky, B. & Rothschild, M.F. (2017). Genomic footprints of dryland stress adaptation in Egyptian fat-tail sheep and their divergence from East African and western Asia cohorts. *Scientific Reports* 7, 17647.

Nayak, R.R., Bernal, W.E., Lee, J.W., Kearns, M.J. & Cheung, V.G. (2014). Stress-induced changes in gene interactions in human cells. *Nucleic Acids Research* 42, 1757-1771.

Olsen, H.G., Knusten TM., Kohler A., Svendsen M., Gidsskehaug L., Grove H., Nome T. *et al*. (2017). Genome-wide association mapping for milk fat composition and fine mapping of a QTL for de novo synthesis of milk fatty acids on bovine chromosome 13. *Genetics Selection Evolution* 49, 20.

Ovilo, C., Benítez, R., Fernández, A., Núñez, Y., Ayuso, M., Fernández, A.I. et al. (2014). Longissimus Dorsi Transcriptome Analysis of Purebred and Crossbred Iberian Pigs Differing in Muscle Characteristics *BMC Genomics* 15, 413.

Prakash, R., Zhang Y., Feng, W., & Jasin, M. (2015). Homologous Recombination and Human Health: The Roles of BRCA1, BRCA2, and Associated Proteins. *Cold Spring Harbor Perspectives in Biology*. 7, a016600.

Ramayo-Caldas, Y., Ballester, M., Fortes, M.R.S., Esteve-Codina, A., Castello, A., Noguera, J.L. et al. (2014). From SNP co-association to RNA co-expression: Novel insights into gene networks for intramuscular fatty acid composition in porcine*. BMC Genomics* 15, 232.

Seroussi, E., Rosov, A., Shirak, A., Lam, A. & Gootwine, E. (2017). Unveiling genomic regions that underlie differences between Afec-Assaf sheep and its parental Awassi breed**.** *Genetics Selection Evolution* 49, 19.

Shen, M., Sun, H., Qu, L., Ma, M., Dou, T., Lu, J, Guo, J., Hu, Y., Wang, X., Li, Y. et al. (2017). Genetic architecture and candidate genes identified for follicle number in chicken. *Scientific Reports* 7, 16412.

Sorbolini, S., Marras, G., Gaspa, G., Dimauro, C., Cellesi, M., Valentini, A. & Macciotta NPP. (2015). Detection of selection signatures in Piemontese and Marchigiana cattle, two breeds with similar production aptitudes but different selection histories*. Genetics Selection Evolution* 47, 52.

Sun, C., Ku, J., Yi, G., Yuan, J., Duan, Z., Qu, L., Xu, G.et al. (2015). Promising loci and genes for yolk and ovary weight in chickens revealed by a genome-wide association study. *PloS One* 10, e0137145.

Tao, X., Liang, Y., Yang, X., Pang, J., Zhong, Z., Chen, X., Yang, Y., Zeng, K., Kang, R. et al. (2017). Transcriptomic profiling in muscle and adipose tissue identifies genes related to growth and lipid deposition. *PLoS One* 12, e0184120.

Wheeler, T.T., Hood, K.A., Maqbool, N.J., McEwan, J.C., Bingle, C.D. & Zhao, S. (2007). Expansion of the Bactericidal/Permeability Increasing-like (BPI-like) protein locus in cattle. *BMC Genomics* 8, 75.

Ye, M., Ye, F., He, L., Luo, B., Yang, F., Cui, C., Zhao, X., Yin, H., Li, D., Xu, H., Wang, Y. & Zhu, Q. (2017). Transcriptomic analysis of chicken myozenin 3 regulation reveals its potential role in cell proliferation. *PLoS One* 12, e0189476
