# Supplementary material for: Ethiopian indigenous goats offer insights into past and recent demographic dynamics and local adaptation in sub‐Saharan African goats
Source: Evol Appl. 2021 Jun 15;14(7):1716–31. doi: 10.1111/eva.13118 (PMC8287980; doi:10.1111/eva.13118)
Supplement: Supplementary file 5 [file EVA-14-1716-s004.odt]

A screenshot of a cell phone

Description automatically generatedA screenshot of a cell phone

Description automatically generatedA screenshot of a cell phone

Description automatically generatedA screenshot of a cell phone

Description automatically generatedA screenshot of a cell phone

Description automatically generatedA picture containing table

Description automatically generatedA screenshot of a cell phone

Description automatically generatedA screenshot of a cell phone

Description automatically generatedA screenshot of a social media post

Description automatically generatedA screenshot of a cell phone

Description automatically generatedA screenshot of a cell phone

Description automatically generated

A screenshot of a cell phone

Description automatically generatedA picture containing table

Description automatically generatedA screenshot of a cell phone

Description automatically generatedA screenshot of a cell phone

Description automatically generatedA picture containing table

Description automatically generatedA screenshot of a cell phone

Description automatically generatedA screenshot of a cell phone

Description automatically generatedA screenshot of a cell phone

Description automatically generatedA screenshot of a cell phone

Description automatically generatedA screenshot of a cell phone

Description automatically generatedA screenshot of a cell phone

Description automatically generatedA screenshot of a cell phone

Description automatically generated
